# Busting contraception myths and misconceptions among youth in Kwale County, Kenya: results of a digital health randomised control trial

**DOI:** 10.1136/bmjopen-2020-047426

**Published:** 2022-01-06

**Authors:** Peter Gichangi, Lianne Gonsalves, Jefferson Mwaisaka, Mary Thiongo, Ndema Habib, Michael Waithaka, Tigest Tamrat, Alfred Agwanda, Hellen Sidha, Marleen Temmerman, Lale Say

**Affiliations:** 1 Administration, Technical University of Mombasa, Mombasa, Kenya; 2 4-PSRI, International Centre for Reproductive Health Kenya, Mombasa, Kenya; 3 Department of Sexual and Reproductive Health and Research including UNDP/UNFPA/UNICEF/WHO/World Bank Special Programme of Research, Development and Research Training in Human Reproduction (HRP), World Health Organization, Geneve, Switzerland; 4 4-PSRI, University of Nairobi, Nairobi, Nairobi, Kenya; 5 4-PSRI, National Council for Population and Development, Nairobi, Kenya; 6 4-PSRI, Aga Khan University - Kenya, Nairobi, Kenya; 7 4-PSRI, International Centre for Reproductive Health, Ghent, Oost-Vlaanderen, Belgium

**Keywords:** reproductive medicine, public health, sexual medicine, clinical trials

## Abstract

**Objectives:**

The objective of this randomised controlled trial in Kenya was to assess the effect of delivering sexual and reproductive health (SRH) information via text message to young people on their ability to reject contraception-related myths and misconceptions.

**Design and setting:**

A three-arm, unblinded randomised controlled trial with a ratio of 1:1:1 in Kwale County, Kenya.

**Participants and interventions:**

A total of 740 youth aged 18–24 years were randomised. Intervention arm participants could access informational SRH text messages on-demand. Contact arm participants received once weekly texts instructing them to study on an SRH topic on their own. Control arm participants received standard care. The intervention period was 7 weeks.

**Primary outcome:**

We assessed change myths believed at baseline and endline using an index of 10 contraception-related myths. We assessed change across arms using difference of difference analysis.

**Results:**

Across arms, <5% of participants did not have any formal education, <10% were living alone, about 50% were single and >80% had never given birth. Between baseline and endline, there was a statistically significant drop in the average absolute number of myths and misconceptions believed by intervention arm (11.1%, 95% CI 17.1% to 5.2%), contact arm (14.4%, 95% CI 20.5% to 8.4%) and control arm (11.3%, 95% CI 17.4% to 5.2%) participants. However, we observed no statistically significant difference in the magnitude of change across arms.

**Conclusions:**

We are unable to conclusively state that the text message intervention was better than text message ‘contact’ or no intervention at all. Digital health likely has potential for improving SRH-related outcomes when used as part of multifaceted interventions. Additional studies with physical and geographical separation of different arms is warranted.

**Trial registration number:**

ISRCTN85156148.

**Strengths and limitations of this study**

- This study included two digital intervention arms, meaning that it would be possible to determine whether changes in outcomes were due to sexual and reproductive health (SRH) content delivered by phone, or participants being ‘nudged’ by phone to think about (and learn about) their SRH.
- The study intentionally did not power sample size around SRH behavioural outcomes, building on previous research that light-touch digital interventions alone may not be enough to see behavioural change in such a complex area of health—instead the primary outcome focuses on SRH knowledge.
- A key limitation is that the study’s individual-level randomisation of young people living near each other is likely to have resulted in contamination between arms.

## Introduction

There is a high unmet need for sexual and reproductive health (SRH) information and services, for both married and unmarried youth worldwide. Data from 61 low-income and middle-income countries show that 33 million women aged 15–24 have an unmet need for contraception.^1^ In Kenya, the 2014 Demographic and Health Survey found that modern contraceptive use among all adolescents age 15–19 years is low (9.3%) compared with all women aged 15–49 years (39.1%).^2^ Partly as a result, the number of adolescents aged 15–19 years who were pregnant or mothers has stagnated at 18% between 2008 and 2014.^2 3^

Sexually active young people face a variety of obstacles to access and use modern contraceptives. They may encounter financial, cultural, social, legal barriers, fear of side effects (eg, infertility and adverse reactions) or cultural norms that restrict their access to contraception services in health facilities.^4–7^ Additionally, contraception myths and misconceptions can negatively affect access to and use of SRH services.^8 9^ Misinformation and myths/misconceptions are often learnt from social networks.^10 11^ In this paper, we describe myths and misconceptions as those being communal or widespread beliefs about effects of contraceptives, which are distinct from individuals’ experiences with contraception-related side effects.^12^

The proliferation of mobile phone technology, and its popularity and ownership with young people in particular,^13 14^ provides an innovative way to educate young people on contraception and their health more broadly. There are indications that health promotion campaigns among adolescents and young people through text messaging may contribute to improved SRH knowledge, behaviours and outcomes.^15^ However, there is less rigorous research and documentation of SRH mobile phone interventions for adolescents and young people in developing countries.^16^ In Kenya in particular, an estimated 93% of households already owned a mobile phone by 2011.^17^

Mobile phone-based digital health interventions have been successfully used in HIV programmes,^18 19^ postabortion care^20^ and to address chronic disease conditions.^21^ Providing broader SRH content, including contraception information, via mobile phones to young people would appear to be a natural strategy to reach them,^22^ increase their contraception knowledge^23^ and improve correct contraception use.^24^ After all, when it comes to ‘sensitive’ SRH content, mobile phones can privately deliver information without stigma or judgement. However, the evidence that digital health interventions can improve youth SRH-related outcomes, including contraception knowledge and uptake, is yet to be significantly established.^25–27^

To address this gap, the WHO’s Department of Sexual and Reproductive Health and Research partnered with research partners in Peru and Kenya to develop the Adolescent/Youth Reproductive Mobile Access and Delivery Initiative for Love and Life Outcomes (ARMADILLO) Study. The ARMADILLO intervention used short message service (SMS, also known as ‘text message’) to deliver SRH information on-demand via a numbers-based menu. The content was developed in the study’s formative stage around several SRH ‘domains’ of interest to policy-makers and young people alike.^28^ The intervention was evaluated using a three-arm randomised controlled trial (RCT). This paper reports on the Kenya study’s primary outcome: are young people with access to the ARMADILLO intervention better able to reject contraception myths and misconceptions as compared with periodic SMS encouraging self-learning or usual care (no intervention).

## Methods

### Study design

This was a three-arm RCT (1:1:1 allocation) involving youth age 18–24 years. The trial ran for 7 weeks, with assessments at baseline and endline. The study methods have been described elsewhere in full,^29^ but are described briefly below.

### Participants and setting

The study was conducted in Kwale County, one of the six counties in the Coastal region of Kenya. The study area consisted of select enumeration areas (EAs) in six Kwale County sublocations which border each other: Ngombeni, Kitivo, Simkumbe, Mkoyo, Gombato and Ukunda. Eligibility criteria was as follows: youth (male and female) aged 18–24; literate; had their own mobile phone at the time of recruitment and reported regular use; reported current use of text messaging.

The Kenya National Bureau of Statistics provided a list of EAs for the six sub-locations. From this list, we randomly selected 21 EAs to be mapped. During the mapping (October 2017), data collectors visited all households and enumerated anyone in the home who was age-eligible to participate. Then, we randomly selected one eligible participant from each household. Starting in February 2018, trained data collectors returned to households and attempted to recruit the selected youth. After consenting to participate, participants completed a baseline survey. Both baseline and endline surveys were implemented by trained data collectors, who entered participants’ responses into a webform on a tablet. For a few sensitive questions relating to previous contraception use and sexual behaviour, participants entered their responses onto the tablet themselves. Participants were then remotely randomised into one of three arms. Intervention and contact arm participants received their first message the following day.

### Interventions

The interventions were categorised as per WHO classification of digital health interventions.^30^ Arm 1 (intervention arm) was an on-demand information service to clients (WHO Classification 1.6). Participants received access to one domain of ARMADILLO content (eg, ‘puberty and anatomy’ or ‘pregnancy prevention’) each week and could request any ‘subdomain’ that interested them from the menu (eg, ‘menstruation’ or ‘physical changes’ for the puberty and anatomy domain or ‘implants’ or ‘male condoms’ for the pregnancy prevention domain) for free for the entire week (see online supplemental figure 1). Arm 2, (contact arm) employed targeted client communication (WHO Classification 1.1). Participants received the same system-initiated contacts as arm 1 participants but without access to the ARMADILLO content itself. Instead, a once-weekly SMS alerted them to an SRH domain for that week (eg, relationships, pregnancy, HIV) and encouraged them to learn on their own (see online supplemental figure 2). At the end of the week, participants in both arms were provided with a single SMS-based quiz question on that domain’s content. If the participant answered (correctly or not), they received a small amount of airtime (1USD). Intervention and contact messages were available in either Swahili or English, per the participant’s preference. All SMS costs were reverse-billed to the study, so intervention and contact arm participants incurred no costs from their participation. Those randomised to arm 3 (control arm) received standard of care (no messages).

10.1136/bmjopen-2020-047426.supp1 Supplementary data

**Figure 1:**
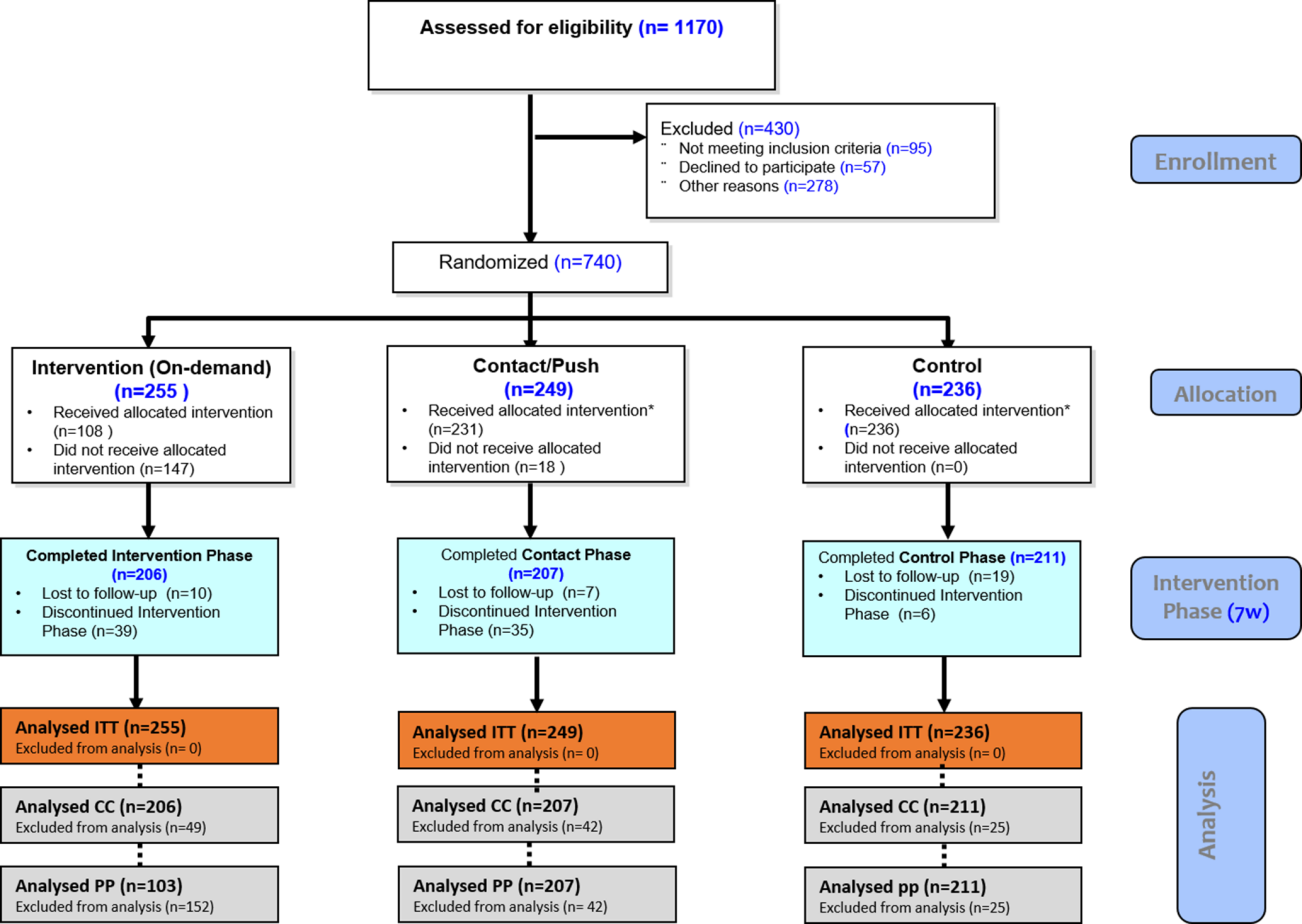
ARMADILLO Kenya’s Consolidated Standards of Reporting Trials diagram. ARMADILLO, Adolescent/Youth Reproductive Mobile Access and Delivery Initiative for Love and Life Outcomes; CC, complete case; PP, per protocol.

**Figure 2:**
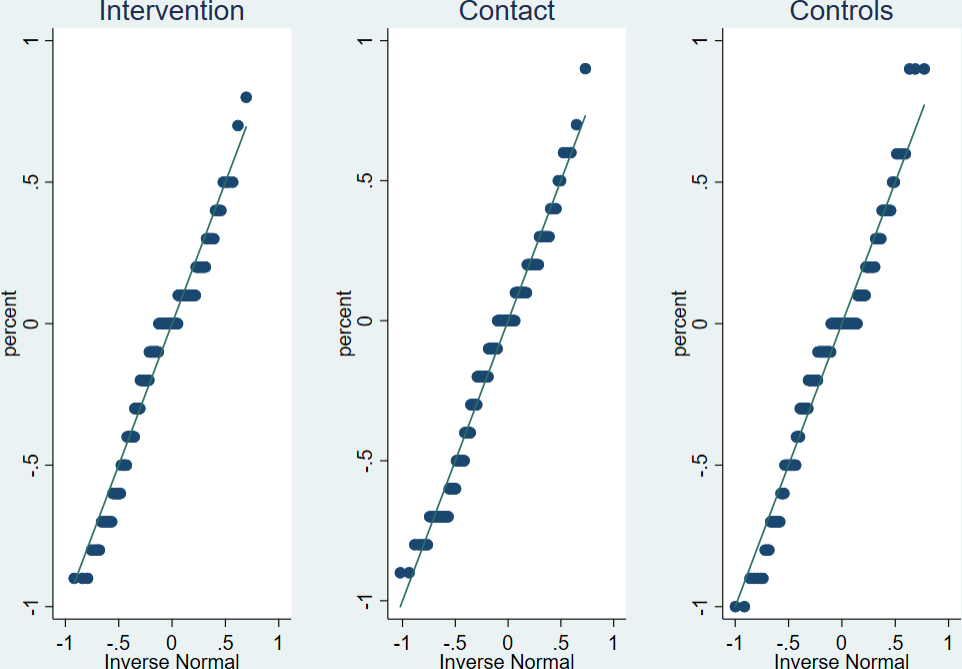
Checking the normality of the absolute changes in the myths believed.

### Assessments and outcome

The primary outcome was assessed using an index developed by the research team of 10 contraception myths and misconceptions (box 1). These were identified based on literature review and a series of focus group discussions with young people prior to the start of the RCT. In these sessions, young people used individual activities and group discussion centred around short vignettes of young couples thinking about starting contraception to describe local concerns around contraception use.^31^ At baseline and endline, RCT participants were asked to state how much they agreed or disagreed with a given statement based on a four-point Likert scale.

Box 1 **Myths and misconceptions**

- Health—People who use contraceptives end up with health problems.
- Body shape—Hormonal contraceptives are fattening.
- Infertility—(1) After a woman uses contraceptive methods, it is difficult to get pregnant, and (2) use of a contraceptive injection can make a woman permanently infertile.
- Harm—Contraceptives can harm a woman’s womb.
- Sex drive—Contraceptives reduce women’s sexual urges.
- Cancer—Contraceptives can cause cancer.
- Malformations—Contraceptives can give you deformed babies.
- Social constructs—(1) Birth control should be a female concern and (2) women who use family planning/birth-spacing may become promiscuous.

### Sample size

The sample size was calculated such that it provided 80% power to detect a 10% change in mean number of myths believed from baseline to endline, assuming that baseline level of belief was 55%, type 1 error at 5% using two-sided Z test with continuity correction and unpooled variance and accounting for a dropout rate of up to 20%. The sample size accounted for Bonferroni correction due to three-arm pairwise comparisons. Based on the aforementioned, a minimum number of participants to be sampled was 705, split evenly across intervention, contact and control arms (1:1:1).

### Randomisation procedures

Participants were individually randomised to either intervention, contact or control group using a ratio of 1:1:1 as per computer-generated randomisation schedule developed using Node.js and docker. All the study participants had equal probability of being assigned to either arm. Allocation took place after the participant had completed the baseline survey. ARMADILLO was an open-label trial; however, neither the technological partner nor the research team had any control of arm assignments.

### Data analysis

The 10 items of the primary outcome were dichotomised from the original Likert scale (strongly agree, agree, disagree and strongly disagree) as follows: (1) agree and strongly agree (participant believed the myth—bad) were recoded as agree and coded as 1; (2) disagree and strongly disagree (participant rejected the myth—good) were recoded as disagree and coded as 0. A participant score for the 10 questions was generated with a total maximum score of 10, corresponding to the number of myths/misconceptions that the participant believed. The average number of myths/misconceptions believed by participants in each arm was computed. There were no missing values for the 10 items across all arms. The study participants responded to all the 10 primary outcome questions in the assessment.

The baseline factors were described using proportions. To ensure that oversampling in certain arms had no effect on the randomisation, we performed $\chi^{2}$ tests on demographic characteristics to confirm that there were no baseline differences between arms. To assess attrition bias (a systematic error caused by unequal loss of participants from the trial between the baseline and the endline), we used Fisher’s exact $\chi^{2}$ tests for the sociodemographic variables to test whether participants lost to follow-up differed across the trial arms (online supplemental table 1) as well as if they differed from those who responded as a function of study group (online supplemental table 2).

**Table 1:** Baseline characteristics of the participants, by study arm (N=740)

Characteristic	Intervention (I)	Contact (P)	Control (C)	Total
	N=255, n (%)	N=249, n (%)	N=236, n (%)	N=740, n (%)
Age of the participant				
18–19 years	62 (24.3)	55 (22.09)	53 (22.5)	170 (23.0)
20–24 years	193 (75.7)	194 (77.91)	183 (77.5)	570 (77.0)
Sex				
Male	134 (52.6)	133 (53.41)	126 (53.4)	393 (53.1)
Female	121 (47.5)	116 (46.59)	110 (46.6)	347 (46.9)
Education level				
Never gone to school	9 (3.5)	7 (2.81)	12 (5.1)	28 (3.8)
Primary school	92 (36.1)	97 (38.96)	80 (33.9)	269 (36.4)
Secondary school	117 (45.9)	118 (47.39)	119 (50.4)	354 (47.8)
Postsecondary education	37 (14.5)	27 (10.84)	25 (10.6)	89 (12.1)
Sublocation				
Ngombeni	34 (13.3)	38 (15.26)	43 (18.2)	115 (15.5)
Kitivo	9 (3.5)	9 (3.61)	9 (3.8)	27 (3.7)
Simkumbe	20 (7.8)	17 (6.83)	21 (8.9)	58 (7.8)
Mkoyo	8 (3.1)	8 (3.21)	8 (3.4)	24 (3.2)
Gombato	17 (6.7)	11 (4.42)	9 (3.8)	37 (5.0)
Ukunda	167 (65.5)	166 (66.67)	146 (61.9)	479 (64.7)
Person currently living with				
Living alone	24 (9.4)	21 (8.43)	21 (8.9)	66 (8.9)
Living with others	231 (90.6)	228 (91.57)	215 (91.1)	674 (91.1)
Current relationship status				
Single	128 (50.2)	121 (48.59)	118 (50.0)	367 (49.6)
Friends with benefits/dating/cohabiting/engaged	109 (42.8)	104 (41.77)	92 (39.0)	305 (41.2)
Married	18 (7.1)	24 (9.64)	26 (11.0)	68 (9.2)
Parity				
None	224 (87.8)	211 (84.7)	191 (80.9)	626 (84.6)
One child	24 (9.4)	28 (11.2)	35 (14.8)	87 (11.8)
2+ children	7 (2.8)	10 (4.0)	10 (4.2)	27 (3.7)
First birth age				
Never given birth	224 (87.8)	211 (84.7)	191 (80.9)	626 (84.6)
≤19 years (adolescents)	18 (7.1)	22 (8.8)	23 (9.8)	63 (8.5)
≥20 years (young women)	13 (5.1)	16 (6.4)	18 (7.6)	47 (6.4)

**Table 2:** Intervention effects for dichotomous outcomes—complete-case analysis

Myth	Control (n=211)			Intervention (n=206)			Contact (n=207)		
	*Baseline	*Endline	Diff.	*Baseline	*Endline	Diff.	*Baseline	*Endline	Diff.
Population based analysis									
Hormonal contraceptives are fattening	139 (65.9%)	117 (55.5%)	−10.4%	144 (69.9%)	135 (65.5%)	−4.4%	142 (68.6%)	127 (61.4%)	−7.2%
Contraceptives can harm a woman’s womb	135 (64%)	114 (54%)	−10%	125 (60.7%)	106 (51.5%)	−9.2%	144 (69.6%)	112 (54.1%)	−15.5%
People who use contraceptives end up with health problems	133 (63%)	103 (48.8%)	−14.2%	120 (58.3%)	105 (51%)	−7.3%	135 (65.2%)	100 (48.3%)	−16.9%
Contraceptives can cause cancer	117 (55.5%)	88 (41.7%)	−13.8%	112 (54.4%)	90 (43.7%)	−10.7%	123 (59.4%)	86 (41.6%)	−17.8%
Use of a contraceptive injection can make a woman permanently infertile	108 (51.2%)	82 (38.9%)	−12.3%	102 (49.5%)	73 (35.4%)	−14.1%	123 (59.4%)	77 (37.2%)	−22.2%
Contraceptives reduce women’s sexual urges	101 (47.9%)	65 (30.8%)	−17.1%	83 (40.3%)	57 (27.7%)	−12.6%	107 (51.7%)	76 (36.7%)	−15%
Contraceptives can give you deformed babies	98 (46.5%)	69 (32.7%)	−13.8%	92 (44.7%)	53 (25.7%)	−19%	106 (51.2%)	71 (34.3%)	−16.9%
After a woman uses contraceptive methods, it is difficult to get pregnant	92 (43.6%)	81 (38.4%)	−5.2%	101 (49%)	69 (33.5%)	−15.5%	102 (49.3%)	80 (38.7%)	−10.6%
Birth control should be a female concern	59 (28%)	41 (19.4%)	−8.6%	46 (22.3%)	32 (15.5%)	−6.8%	59 (28.5%)	35 (16.9%)	−11.6%
Women who use family planning/birth-spacing may become promiscuous	109 (51.7%)	92 (43.6%)	−8.1%	108 (52.4%)	84 (40.8%)	−11.6%	111 (53.6%)	89 (43%)	−10.6%
Subject specific analysis									
Average # myths believed, per participant (SE)	**5.17**	**4.04**		**5.01**	**3.90**		**5.57**	**4.12**	
Average absolute change in myths believed	−**1.13**			−**1.11**			−**1.44**		
CI of the diff.	**(−1.59 to –0.67**)			**(−1.54 to –0.68**)			**(−1.91 to –0.98**)		
Percentage absolute change in myths believed	−**21.9**			−**22.2**			−**25.9**		
Test of diff. in the mean of the absolute change in myths believed	Control (n=211)			Intervention (n=206)			Contact (n=207)		
ANOVA test F statistics	**0.66**								
P value	**0.5181**								

n*—Those who believed the myth (had wrong answer).

Numbers in bold are for variables analysed under subject specific analysis

ANOVA, analysis of variance.

First, we present proportions of those who believed in the myths at baseline and endline for all arms and the percentage change in the myths believed between the two periods. To obtain the average number of myths believed per participant, we computed (using the sum of the dichotomised 10-item response) the number of myths believed for each participant at baseline and at endline. Then for each participant, we computed the average number of myths believed (expressed as a percentage) at the baseline and at endline (by dividing the sum of myths believed by 10 and multiplying by 100). Next, for each participant, his or her absolute change in the average myths believed between the endline and baseline was computed (endline minus baseline).

Normality of the absolute changes in the myths believed was tested using the quantile-quantile plots. As the distribution of the absolute changes in the myths believed was normally distributed, analysis of variance (ANOVA) test of equality of group means was used to test the between group differences in the means of the absolute myths change. We estimated the difference-in-differences (DID) of the average number of myths believed by participants in a given arm to evaluate the effect of the ARMADILLO intervention to dispel myths and misconceptions about contraception. DID tells us whether the expected mean change in the number of myths and misconceptions believed from baseline to endline was different in the groups compared. DID is calculated by subtracting the average of the outcome in the control or contact arm from the average of the outcome in the intervention arm (${\hat{d}}_{1}$), where the outcome is the change in percentage number of myths believed by each individual between the endline and baseline. DID was also used to assess changes in the average proportion of myths believed per participant between the control and the contact arm (${\hat{d}}_{2}$).

$$\begin{array}{ll} {\hat{d}}_{1}= & \left\{{\left[Mean\left(Y_{i}\left(Endline\right)\right)-Mean\left(Y_{i}\left(Baseline\right)\right)\right]}_{Intervention}\right\}\\ & \left\{{\left[Mean\left(Y_{i}\left(Endline\right)\right)-Mean\left(Y_{i}\left(Baseline\right)\right)\right]}_{j}\right\} \end{array}$$

$$\begin{array}{ll} {\hat{d}}_{2}= & \left\{{\left[Mean\left(Y_{i}\left(Endline\right)\right)-Mean\left(Y_{i}\left(Baseline\right)\right)\right]}_{Contact}\right\}-\\ & \left\{{\left[Mean\left(Y_{i}\left(Endline\right)\right)-Mean\left(Y_{i}\left(Baseline\right)\right)\right]}_{Control}\right\} \end{array}$$

Where *i* refer to the ith individual in the trial arm; while *j*=contact or control

All analyses were based on complete-case (CC) dataset while analyses based on per-protocol (PP) dataset were used for sensitivity analysis. Participants were included in the analysis provided that they had completed both baseline and endline surveys. In this case, the ITT analysis was equivalent to the CC.

Participants were included in the PP analysis provided that they had completed baseline and endline surveys, and that the intervention system could confirm that they had (1) received the ARMADILLO message domain associated with the primary outcome (pregnancy prevention); and (2) requested at least one message from this domain. Results with a type I error of p<0.05 in two-sided tests were considered statistically significant. Where Bonferroni correction was applied for the pairwise comparisons of the three study arms, p<0.017 were considered statistically significant. Analyses were performed using Stata V.15, and all were conducted in accordance with a prespecified statistical analysis plan.

### Patients and public involvement statement

ARMADILLO’s population of ‘young people’ were involved in the study from its initial, formative stage,^28 32^ which included message content development. They and the broader community continued engagement through this trial through the ARMADILLO community advisory board (CAB) consisting of representatives from the Ministries of Health, Education and Social Services; youth-led organisations; area chiefs; healthcare workers providing SRH services to adolescents; and young people themselves. CAB members provided technical and field support throughout the data collection period. Additionally, young people identified from the study area were trained as data collectors and hired to enumerate young people in the area as well as recruit and implement baseline and endline surveys with study participants. Young data collectors’ input also shaped recruitment hours and strategies. A dissemination involving local and national stakeholders took place in July 2019—selected young data collectors participated in the dissemination meeting and shared their feedback.

## Results

A total of 740 men and women aged 18–24 years were randomised into intervention, contact and control arms (figure 1).

In the intervention period which lasted 7 weeks, 116 of the 740 (16%) study participants dropped out over the course of the 7-week intervention period (intervention arm—49 (19%); contact arm—42 (17%); control arm—25 (11%)). Among participants in the intervention arm, 206 (81%) completed both the baseline and the endline assessments (making them eligible for CC analysis) while 103 (40%) were eligible for PP analysis. Among the contact group, 207 (83%) received the push messages and were included in both the CC and PP analysis. In the control group, 211 (89.4%) completed both the baseline and the endline assessments. Baseline characteristics for the study sample are shown in table 1. Across all arms, 53% of the participants were male, 48% had a secondary education or higher, 65% were from Ukunda sublocation, 91% lived with others and 85% did not have children. There were no significant baseline differences between the intervention, contact and control groups.

Concerning attrition bias, the analysis revealed that participants who dropped out in each arm were similar to each other (online supplemental table 1). However, there was a significant association between dropping out of the study and the number of children the participant had at the time of the study in the control and the contact groups’ participants, online supplemental table 1. The analysis assessing attrition bias also revealed that there was no significant difference in the sociodemographics between the participants who were lost to follow-up and those who finished the 7 weeks of the intervention and took the endline survey, online supplemental table 2.

The results of the CC analysis examining the myths and misconceptions believed are displayed in table 2. The myths are ordered by those which were believed by the most number of participants, across groups, with the most salient myth at the top. The results show that at baseline, study participants in all arms believed around half of the myths related to contraception on average. At the end of the 7-week intervention period, the average number of myths and misconceptions believed per participant had significantly decreased for all the three groups (p<0.0001). The average absolute decrease in the myths believed was 11.1% among the intervention group (mean −11.1%; 95% CI −17.1% to −5.2%); 14.4% among the contact group (mean −14.4%; 95% CI −20.5% to −8.4%) and 11.3% among the control group (mean −11.3%; 95% CI −17.4% to −5.2%). From the normality test shown in figure 2, the absolute change in all the three groups were normally distributed. The ANOVA test of equality-of-populations means showed that there was no significant difference in the group medians (p=0.5181).

As presented in the DID analysis in table 3, there was no statistically significant differences between the baseline to endline decrease observed across the three arms (p>0.017-Bonferroni corrected significance level).

**Table 3:** Difference in difference analysis

Outcome	Percentage point differences (95% CI)	P value
Contraception myths and misconceptions index score (endline – baseline assessment)		
Arm 1: Intervention (Mean∆, 95% CI)	−11.1% (−17.1% to −5.2%)	<0.001
Arm 2: Contact (Mean∆, 95% CI)	−14.4% (−20.5% to −8.4%)	<0.001
Arm 3: Control (Mean∆, 95% CI)	−11.3% (−17.4% to −5.2%)	<0.001
Mean (∆ Intervention) − Mean (∆ Control)	0.2% (−8.3% to 8.7%)	0.961
Mean (∆ Contact) − Mean (∆ Control)	−3.1% (−11.7% to 5.4%)	0.475
Mean (∆ Intervention) − Mean (∆ Contact)	3.3% (−5.1% to 11.8%)	0.440

∆ refers to the subject-specific change in the outcome from baseline to endline. 95% CI refers to the 95% CI. A generalised linear model using a normal distribution and identity link was used to compare scores.

Effective sample size used for the analysis was 206 participants for the intervention group; 207 participants for the contact group and 211 participants for the control group.

Sensitivity analysis including only participants who met PP inclusion criteria did not alter the findings from CC analysis. As the results of the CC analysis, and PP analysis did not differ with respect to statistical significance within groups or between groups, only those for the CC analysis are reported (PP analysis is attached as online supplemental table 3).

Finally, table 4 shows estimates of a possible source of contamination between study arms. Of the 23% of the participants in the intervention group who shared the messages with others, 13%, 5% and 4% shared the messages with friends, partners and multiple contacts, respectively. Among the 27% contact group participants who shared the messages with others, 15%, 8%, 1% and 3% shared the messages with friends, partners, siblings and multiple contacts, respectively.

**Table 4:** Study contamination

		Shared the messages	Shared the messages with:				
			Siblings	Friends	Partner	Parents	Multiple contacts
Intervention (N=206)	n	47	0	26	10	2	9
	%	22.8	0.0	12.6	4.9	1.0	4.4
Contact (N=207)	n	55	2	30	16	0	7
	%	26.6	1.0	14.5	7.7	0.0	3.4
Total (N=413)	n	102	2	56	26	2	16
	%	24.7	0.5%	13.6%	6.3%	0.5%	3.9%

## Discussion

Our findings suggest that provision of SRH content via SMS is potentially useful in dealing with contraception-related myths and misconceptions among youth. Across arms, the study demonstrated between a 11% and 14% reduction in the average number of myths/misconception statements believed over the study period. However, we did not observe a significant difference in the magnitude of reduction between the arms. Therefore, despite the significant decrease in myths-believed that we observed between baseline and endline, we are unable to conclusively state that the ARMADILLO intervention was better than SMS ‘contact’ or no intervention at all.

One possible reason for not seeing a significant effect of the ARMADILLO intervention versus no intervention is that correcting false information is difficult. Studies aimed at correcting misinformation about vaccines, for example, have shown that even when attempts to correct invalid information do not entrench the original misinformation, they can frequently fail because people cannot successfully update their memories, and still fall back on information they know is not correct.^33–36^ A 7-week digital health intervention, dedicated to SRH broadly, may not have been enough to dispel deeply entrenched concerns about contraception. Myths and misconceptions around contraception are also particularly tricky given that misconception about contraception generally (eg, that contraception use can lead to infertility) may be partially rooted in individual experiences of real side effects (eg, the possible delay of a return to fertility following use of injectable contraception).^12^

Alternatively, we may have seen no difference between the arms because the intervention was truly not better than SMS-prompted self-learning and/or no intervention at all. Several other RCTs that have attempted to tie adolescent-targeted digital health interventions to SRH knowledge, acceptability or behavioural outcomes have resulted in similarly inconclusive findings,^26 37–40^ indicating that digital interventions on their own may not be enough to encourage behavioural change. However, while the above reasons would explain the lack of difference between arms, they do not explain why participants in all arms believed significantly fewer myths at endline than they did at baseline.

Here, contamination between arms may be to blame. RCT of digital health client communication interventions are difficult, especially when the intervention content can be easily shared between neighbours and across communities. To the best of our knowledge, there were no other health campaigns or interventions aimed at dispelling the myths and misconceptions in the area during the study period. However, about a quarter of the intervention and contact arm participants reported sharing information with study participants and other members of the community. Any effect of participants sharing information was amplified by our participants being randomised at the individual level. This resulted in participants in different arms living in close proximity, often in neighbouring households. ARMADILLO intervention arm participants may therefore have received the messages and shared them with their friends/neighbours, some of whom were ARMADILLO contact and control arm participants.

Addressing/dispelling myths and misconceptions among youth is particularly important for future contraception use. By nature of originating in social networks as well as their likelihood to ‘stick’ indefinitely, myths and misconceptions among youth should be dispelled early to prevent their becoming engrained.^6 41^ Indeed, our study found that two of the three most commonly-believed myths and misconceptions among youth age 18–24 years (people who use contraceptives end up with health problems, and contraceptives can harm a woman’s womb) were also the top myths for youth and adult women aged 15–49 years in Kenya, Nigeria and Senegal.^9^

Other studies have reported successfully addressing myths and misconceptions with dedicated community and communication interventions,^42^ involving a variety of opinion leaders and channels.^9 42^ Following a 4-year intervention using radio, religious leaders and community health volunteers, for example, Kenya’s Tupange study reported a 15% decrease in the number of women who believed myths and misconceptions statements between baseline and endline.^42^

Digital interventions are an additional channel which can be included in this mix. There is a wealth of opportunity to engage with young people en masse not only through SMS and voice channels, but also by widely used messaging and social media platforms like WhatsApp, Facebook and Facebook Messenger, the latter of which don’t have the bulk telecom-related costs of SMS and voice interventions. The popularity of the ARMADILLO interventions among its users—one silver lining of the contamination between arms—indicate that such interventions can be considered as one tool in a multipronged approach targeting young people with correct SRH information.^25 43–45^ However, evidence continues to be needed on adolescent-targeted client communication interventions in general.

This study is not without limitations. The lack of differences between the intervention and the other arms could very well be due to our adoption of individually randomised rather than cluster randomised study design. A cluster design was considered; however, the accessibility and make-up of the study area did not allow for homogeneous clusters to be created (Ukunda, eg, is unique in Kwale County for its population density). While unfortunate for the results of the trial, it demonstrates that the messages were as popular as the research team hoped and provides some positive insight as to the dissemination which might take place if a similar intervention is implemented outside of a research setting. The study is strengthened by its choice of primary outcome: we intentionally avoided powering the study around SRH behavioural outcomes, building on learnings from previous studies that light-touch digital interventions alone may not be enough to see behavioural change in such a complex area of health.

In conclusion, creative and consistent interventions are needed to address deeply rooted myths and misconceptions young people have about contraception and mitigate one important driver of anxiety around contraception use in Kenya. These can include digital interventions. However, while the ARMADILLO study saw some promising results, we cannot conclusively say that digital interventions alone are sufficient to affect change in SRH-related outcomes. Additional research (using alternative designs) will be required to identify the specific value of digital targeted client communication programmes in addressing young people’s contraception myths/misconceptions and improving SRH knowledge overall.

## Supplementary Material

Reviewer comments

Author's manuscript

## Data Availability

Data are available on reasonable request. De-identified data related to this article can be obtained by reasonable request to the corresponding author (ORCID: 0000-0001-9636-165X).

## References

[1] MacQuarrie KLD. Unmet need for family planning among young women: levels and trends. Rockville, Maryland, USA: ICF International, 2014.

[2] Kenya National Bureau of Statistics. Kenya demographic and health survey 2014. Rockville, Maryland: The DHS Program, ICF International, 2015.

[3] Kenya National Bureau of Statistics (KNBS). Kenya demographic and health survey 2008-09. Calverton, Maryland: KNBS and ICF Macro, 2010.

[4] Chandra-Mouli V, McCarraher DR, Phillips SJ, et al. Contraception for adolescents in low and middle income countries: needs, barriers, and access. Reprod Health 2014;11. 10.1186/1742-4755-11-1 PMC3882494 24383405

[5] Denno DM, Hoopes AJ, Chandra-Mouli V. Effective strategies to provide adolescent sexual and reproductive health services and to increase demand and community support. J Adolesc Health 2015;56:S22–41. 10.1016/j.jadohealth.2014.09.012 25528977

[6] Speizer IS, Calhoun LM, Guilkey DK. Reaching urban female adolescents at key points of sexual and reproductive health transitions: evidence from a longitudinal study from Kenya. Afr J Reprod Health 2018;22:47–59. 10.29063/ajrh2018/v22i1.5 29777642 PMC6639007

[7] Parker JJ, Veldhuis CB, Hughes TL, et al. Barriers to contraceptive use among adolescents in two semi-rural Nicaraguan communities. Int J Adolesc Med Health 2019;32. 10.1515/ijamh-2017-0228. [Epub ahead of print: 02 Apr 2019]. 30939115

[8] AAAJO M. Myths, misinformation, and communication about family planning and contraceptive use in Nigeria. Open Access Journal of Contraception 2011;2:95–105.

[9] Gueye A, Speizer IS, Corroon M, et al. Belief in family planning myths at the individual and community levels and modern contraceptive use in urban Africa. Int Perspect Sex Reprod Health 2015;41:191–9. 10.1363/intsexrephea.41.4.0191 26871727 PMC4858446

[10] Paz Soldan VA. How family planning ideas are spread within social groups in rural Malawi. Stud Fam Plann 2004;35:275–90. 10.1111/j.0039-3665.2004.00031.x 15628785

[11] Yee L, Simon M. The role of the social network in contraceptive decision-making among young, African American and Latina women. J Adolesc Health 2010;47:374–80. 10.1016/j.jadohealth.2010.03.014 20864007 PMC2945601

[12] PATH. Countering myths and misperceptions about contraceptives. Seattle, United States: PATH, 2015.

[13] Hightow-Weidman LB, Muessig KE, Bauermeister J, et al. Youth, technology, and HIV: recent advances and future directions. Curr HIV/AIDS Rep 2015;12:500–15. 10.1007/s11904-015-0280-x 26385582 PMC4643403

[14] World Bank. The little data book on information and communication technology 2014. Washington DC: World Bank, 2014.

[15] L'Engle KL, Mangone ER, Parcesepe AM, et al. Mobile phone interventions for adolescent sexual and reproductive health: a systematic review. Pediatrics 2016;138. 10.1542/peds.2016-0884. [Epub ahead of print: 23 08 2016]. 27553221

[16] Ippoliti NB, L'Engle K. Meet us on the phone: mobile phone programs for adolescent sexual and reproductive health in low-to-middle income countries. Reprod Health 2017;14:11. 10.1186/s12978-016-0276-z 28095855 PMC5240300

[17] Demombynes GT A. Kenya’s Mobile Revolution and the Promise of Mobile Savings. World Bank Group, 2012.

[18] Kalichman SC, Cherry C, Kalichman MO, et al. Mobile health intervention to reduce HIV transmission: a randomized trial of behaviorally enhanced HIV treatment as prevention (B-TasP). J Acquir Immune Defic Syndr 2018;78:34–42. 10.1097/QAI.0000000000001637 29406429 PMC5889341

[19] Widman L, Nesi J, Kamke K, et al. Technology-Based interventions to reduce sexually transmitted infections and unintended pregnancy among youth. J Adolesc Health 2018;62:651–60. 10.1016/j.jadohealth.2018.02.007 29784112 PMC5966833

[20] Smith C, Ngo TD, Gold J, et al. Effect of a mobile phone-based intervention on post-abortion contraception: a randomized controlled trial in Cambodia. Bull World Health Organ 2015;93:842–50. 10.2471/BLT.15.160267 26668436 PMC4669734

[21] Anderberg P, Barnestein-Fonseca P, Guzman-Parra J, et al. The effects of the digital platform support monitoring and reminder technology for mild dementia (SMART4MD) for people with mild cognitive impairment and their informal carers: protocol for a pilot randomized controlled trial. JMIR Res Protoc 2019;8:e13711. 10.2196/13711 31228177 PMC6611150

[22] Vahdat HL, L'Engle KL, Plourde KF, et al. There are some questions you may not ask in a clinic: providing contraception information to young people in Kenya using SMS. Int J Gynaecol Obstet 2013;123 Suppl 1:e2–6. 10.1016/j.ijgo.2013.07.009 24012514

[23] Rokicki S, Fink G. Assessing the reach and effectiveness of mHealth: evidence from a reproductive health program for adolescent girls in Ghana. BMC Public Health 2017;17:969. 10.1186/s12889-017-4939-7 29262823 PMC5738156

[24] Castaño PM, Bynum JY, Andrés R, et al. Effect of daily text messages on oral contraceptive continuation: a randomized controlled trial. Obstet Gynecol 2012;119:14–20. 10.1097/AOG.0b013e31823d4167 22143257

[25] Garofalo R, Kuhns LM, Hotton A, et al. A randomized controlled trial of personalized text message reminders to promote medication adherence among HIV-positive adolescents and young adults. AIDS Behav 2016;20:1049–59. 10.1007/s10461-015-1192-x 26362167 PMC4788595

[26] Lim MSC, Hocking JS, Aitken CK, et al. Impact of text and email messaging on the sexual health of young people: a randomised controlled trial. J Epidemiol Community Health 2012;66:69–74. 10.1136/jech.2009.100396 21415232

[27] World Health Organization. Who guideline: recommendations on digital interventions for health system strengthening web supplement 2: summary of findings and grade tables, Report No.: WHO/RHR/19.7. Geneva: WHO, 2019. 31162915

[28] Gonsalves L, L'Engle KL, Tamrat T, et al. Adolescent/Youth reproductive mobile access and delivery initiative for love and life outcomes (armadillo) study: formative protocol for mHealth platform development and piloting. Reprod Health 2015;12:67. 10.1186/s12978-015-0059-y 26248769 PMC4527108

[29] Gonsalves L, Hindin MJ, Bayer A, et al. Protocol of an open, three-arm, individually randomized trial assessing the effect of delivering sexual and reproductive health information to young people (aged 13–24) in Kenya and Peru via mobile phones: adolescent/youth reproductive mobile access and delivery initiative for love and life outcomes (armadillo) study stage 2. Reprod Health 2018;15. 10.1186/s12978-018-0568-6 PMC6042417 29996854

[30] World Health Organization. Classification of digital health interventions v1.0. Geneva, Switzerland: World Health Organization, 2018.

[31] Mwaisaka J, Gonsalves L, Thiongo M, et al. Exploring contraception myths and misconceptions among young men and women in Kwale County, Kenya. BMC Public Health 2020;20:1694. 10.1186/s12889-020-09849-1 33176738 PMC7661170

[32] Mwaisaka JG, Lianne S, Gichangi P. “What’s that on your phone?” The aftermath of parents finding sexual and reproductive health messages on their children’s phone in coastal Kenya. Journal of Health and Social Sciences 2018;3:147–56.

[33] Chan M-PS, Jones CR, Hall Jamieson K, et al. Debunking: a meta-analysis of the psychological efficacy of messages Countering misinformation. Psychol Sci 2017;28:1531–46. 10.1177/0956797617714579 28895452 PMC5673564

[34] Lewandowsky S, Ecker UKH, Seifert CM, et al. Misinformation and its correction: continued influence and successful Debiasing. Psychol Sci Public Interest 2012;13:106–31. 10.1177/1529100612451018 26173286

[35] Pluviano S, Watt C, Della Sala S. Misinformation lingers in memory: failure of three pro-vaccination strategies. PLoS One 2017;12:e0181640. 10.1371/journal.pone.0181640 28749996 PMC5547702

[36] Seifert CM. The continued influence of misinformation in memory: what makes a correction effective? In: Psychology of learning and motivation. 41. Academic Press, 2002: 265–92.

[37] Free C, McCarthy O, French RS, et al. Can text messages increase safer sex behaviours in young people? intervention development and pilot randomised controlled trial. Health Technol Assess 2016;20:1–82. 10.3310/hta20570 PMC4983705 27483185

[38] Perry RCW, Kayekjian KC, Braun RA, et al. Adolescents' perspectives on the use of a text messaging service for preventive sexual health promotion. J Adolesc Health 2012;51:220–5. 10.1016/j.jadohealth.2011.11.012 22921131

[39] McCarthy O, Ahamed I, Kulaeva F, et al. A randomized controlled trial of an intervention delivered by mobile phone APP instant messaging to increase the acceptability of effective contraception among young people in Tajikistan. Reprod Health 2018;15:28. 10.1186/s12978-018-0473-z 29433506 PMC5809875

[40] McCarthy OL, Aliaga C, Torrico Palacios ME, et al. An intervention delivered by mobile phone instant messaging to increase acceptability and use of effective contraception among young women in Bolivia: randomized controlled trial. J Med Internet Res 2020;22:e14073. 10.2196/14073 32568092 PMC7338928

[41] da Silva-Filho AL, Lira J, Rocha ALL, et al. Barriers and myths that limit the use of intrauterine contraception in nulliparous women: a survey of Brazilian gynaecologists. Postgrad Med J 2017;93:376–81. 10.1136/postgradmedj-2016-134247 27780879

[42] Muthamia M, Owino K, Nyachae P, et al. The Tupange project in Kenya: a multifaceted approach to increasing use of long-acting reversible contraceptives. Glob Health Sci Pract 2016;4 Suppl 2:S44–59. 10.9745/GHSP-D-15-00306 27540124 PMC4990161

[43] L'Engle K, Plourde KF, Zan T. Evidence-Based adaptation and scale-up of a mobile phone health information service. Mhealth 2017;3:11. 10.21037/mhealth.2017.02.06 28567408 PMC5427190

[44] Lee S, Begley CE, Morgan R, et al. Addition of mHealth (mobile health) for family planning support in Kenya: disparities in access to mobile phones and associations with contraceptive knowledge and use. Int Health 2019;11:463–71. 10.1093/inthealth/ihy092 30576546

[45] McCarthy OL, Zghayyer H, Stavridis A, et al. A randomized controlled trial of an intervention delivered by mobile phone text message to increase the acceptability of effective contraception among young women in Palestine. Trials 2019;20:228. 10.1186/s13063-019-3297-4 31014358 PMC6477750

