# Supplementary material for: Busting contraception myths and misconceptions among youth in Kwale County, Kenya: results of a digital health randomised control trial
Source: BMJ Open. 2022 Jan 6;12(1):e047426. doi: 10.1136/bmjopen-2020-047426 (PMC8739061; doi:10.1136/bmjopen-2020-047426)
Supplement: Supplementary data [file bmjopen-2020-047426supp001.pdf]

Supplementary materials

Suppl. table 1: Assessing attrition bias across the study arms

Characteristic	Control (C)	Intervention (I)	Contact (P)	Assessing the baseline differences for drop-outs		
				C vs I	I vs P	C vs P
	N=25, n (%)	N=49, n (%)	N=42, n (%)	p-value	p-value	p-value
**Age of the participant**						
18 – 19	5 (20.0)	14 (28.57)	13 (30.95)	0.58	0.82	0.24
20 – 24	20 (80.0)	35 (71.43)	29 (69.05)			
**Sex**						
Male	12 (48.0)	24 (48.98)	21 (50.0)	1.00	1.00	1.00
Female	13 (52.0)	25 (51.02)	21 (50.0)			
**Highest level of school attended**						
Never gone to school	2 (8.0)	1 (2.04)	0 (0)	0.41	0.68	0.42
Primary school	8 (32.0)	21 (42.86)	16 (38.1)			
Secondary school	12 (48.0)	24 (48.98)	21 (50)			
Post-secondary education	3 (12.0)	3 (6.12)	5 (11.9)			
**Sub-location**						
Ngombeni	2 (8.0)	9 (18.37)	10 (23.81)	0.70	0.69	0.31
Kitivo	1 (4.0)	0 (0)	0 (0)			
Simkumbe	1 (4.0)	2 (4.08)	3 (7.14)			
Mkoyo	1 (4.0)	3 (6.12)	2 (4.76)			
Gombato	1 (4.0)	2 (4.08)	4 (9.52)			
Ukunda	19 (76.0)	33 (67.35)	23 (54.76)			
**Person currently living with**						
Living alone	1 (4.0)	4 (8.16)	3 (7.14)	0.66	1.00	1.00
Living with others	24 (96.0)	45 (91.84)	39 (92.86)			

Gichangi P, *et al. BMJ Open* 2022; 12:e047426. doi: 10.1136/bmjopen-2020-047426

**Current relationship status**						
Single	12 (48.0)	28 (57.14)	23 (54.76)	0.36	0.69	0.83
Friends with benefits / Dating / Cohabiting / Engaged	9 (36.0)	18 (36.73)	14 (33.33)			
Married	4 (16.0)	3 (6.12)	5 (11.9)			
**Number of children the participant have**						
None	16 (64.0)	41 (83.67)	37 (88.1)	0.14	0.87	**0.03****
1 child	7 (28.0)	7 (14.29)	5 (11.9)			
2+ children	2 (8.0)	1 (2.04)	0 (0)			
**First birth age**						
Never given birth	16 (64.0)	41 (83.67)	37 (88.1)	0.42	0.90	0.15
<=19 years (Adolescents)	5 (20.0)	5 (10.2)	3 (7.14)			
>=20 years (Young women)	2 (8.0)	3 (6.12)	2 (4.76)			
Missing	2 (8.0)	0 (0)	0 (0)			

Different rates of loss to follow up in the exposure groups, or losses of different types of participants, whether at similar or different frequencies may change the characteristics of the groups, irrespective of the exposure or intervention. This analysis was done to confirm if there were systematic differences among the people who were lost to follow up across the different study arms.

Gichangi P, *et al. BMJ Open* 2022; 12:e047426. doi: 10.1136/bmjopen-2020-047426

Suppl. table 2: Assessing attrition bias (drop-outs vs those who took the endline assessment)

Characteristic	Intervention (I)			Contact (P)			Control (C)		
	Drop-outs	Endline participants		Drop-outs	Endline participants		Drop-outs	Endline participants	
	N=49, n (%)	N=206, n (%)	Fisher's test p-value	N=42, n (%)	N=207, n (%)	Fisher's test p-value	N=25, n (%)	N=211, n (%)	Fisher's test p-value
**Age of the participant**									
18 – 19 years (Adolescents)	14 (28.57)	48 (23.3)	0.46	13 (30.95)	42 (20.3)	0.15	5 (20.0)	48 (22.8)	0.49
20 – 24 years (Young women)	35 (71.43)	158 (76.7)		29 (69.05)	165 (79.7)		20 (80.0)	163 (77.2)	
**Sex**									
Male	24 (48.98)	110 (53.4)	0.63	21 (50.0)	112 (54.1)	0.74	12 (48.0)	114 (54.0)	0.67
Female	25 (51.02)	96 (46.6)		21 (50.0)	95 (45.90		13 (52.0)	97 (46.0)	
**Highest level of school attended**									
Never gone to school	1 (2.04)	8 (3.9)	0.23	0 (0)	7 (3.4)	0.83	2 (8.0)	10 (4.7)	0.85
Primary school	21 (42.86)	71 (34.5)		16 (38.1)	81 (39.1)		8 (32.0)	72 (34.1)	
Secondary school	24 (48.98)	93 (45.2)		21 (50)	97 (46.9)		12 (48.0)	107 (50.7)	
Post-secondary education	3 (6.12)	34 (16.5)		5 (11.9)	22 (10.6)		3 (12.0)	22 (10.4)	
**Sublocation**									
Ngombeni	9 (18.37)	25 (12.1)	0.26	10 (23.81)	28 (13.5)	0.1	2 (8.0)	41 (19.4)	0.55
Kitivo	0 (0)	9 (4.4)		0 (0)	9 (4.4)		1 (4.0)	8 (3.8)	
Simkumbe	2 (4.08)	18 (8.7)		3 (7.14)	14 (6.8)		1 (4.0)	20 (9.5)	
Mkoyo	3 (6.12)	5 (2.4)		2 (4.76)	6 (2.9)		1 (4.0)	7 (3.3)	
Gombato	2 (4.08)	15 (7.3)		4 (9.52)	7 (3.4)		1 (4.0)	8 (3.8)	
Ukunda	33 (67.35)	134 (65.1)		23 (54.76)	143 (69.1)		19 (76.0)	127 (60.2)	
**Person currently living with**									
Living alone	4 (8.16)	20 (9.7)	1	3 (7.14)	18 (8.7)	1	1 (4.0)	20 (9.5)	0.71
Living with others	45 (91.84)	186 (90.3)		39 (92.86)	189 (91.3)		24 (96.0)	191 (90.5)	
Missing									
**Current relationship status**									
Single	28 (57.14)	100 (48.5)	0.58	23 (54.76)	98 (47.3)	0.41	12 (48.0)	106 (50.2)	0.68

Friends with benefits / Dating / Cohabiting / Engaged	18 (36.73)	15 (7.3)		14 (33.33)	19 (9.2)		9 (36.0)	22 (10.4)	
Married	3 (6.12)	91 (44.20		5 (11.9)	90 (43.5)		4 (16.0)	83 (39.3)	
**Number of children the participant have**									
None	41 (83.7)	183 (88.8)	0.4	37 (88.1)	174 (84.1)	0.46	16 (64.0)	175 (82.9)	0.06
1 child	7 (14.29)	17 (8.3)		5 (11.9)	23 (11.1)		7 (28.0)	28 (13.3)	
2+ children	1 (2.04)	6 (2.9)		0 (0)	10 (4.8)		2 (8.0)	8 (3.8)	
**First birth age**									
Never given birth	41 (83.67)	183 (88.8)	0.5	37 (88.1)	174 (84.1)	0.94	16 (64.0)	175 (82.9)	0.08
<=19 years (Adolescents)	5 (10.2)	13 (6.3)		3 (7.14)	19 (9.2)		5 (20.0)	18 (8.5)	
>=20 years (Young women)	3 (6.12)	10 (4.9)		2 (4.76)	14 (6.8)		2 (8.0)	16 (7.6)	
Missing	0 (0)	0		0 (0)	0		2 (8.0)	2 (1.0)	

Systematic difference between people who leave the study and those who continue can introduce bias in the study results, this is attrition bias. Suppl. table 2 shows the assessment of attrition bias which was achieved using fishers chi-square tests for categorical variables. This assessment was done to test whether participants lost to follow up differed from those who responded as a function of study group.

Suppl. table 3: Per Protocol analysis

Myth	Intervention (I) (n=103)			Contact (P) (n=207)			Control (C) (n=211)		
	Baseline	Endline	Diff.	Baseline	Endline	Diff.	Baseline	Endline	Diff.
**Population based analysis**									
Hormonal contraceptives are fattening	72 (69.9%)	71 (68.9%)	-1.00%	142 (68.6%)	127 (61.4%)	-7.20%	139 (65.9%)	117 (55.5%)	-10.40%
Contraceptives can harm a woman's womb	62 (60.2%)	49 (47.6%)	-12.60%	144 (69.6%)	112 (54.1%)	-15.50%	135 (64%)	114 (54%)	-10%
People who use contraceptives end up with health problems	60 (58.3%)	52 (50.5%)	-7.80%	135 (65.2%)	100 (48.3%)	-16.90%	133 (63%)	103 (48.8%)	-14.20%
Contraceptives can cause cancer	59 (57.3%)	47 (45.6%)	-11.70%	123 (59.4%)	86 (41.6%)	-17.80%	117 (55.5%)	88 (41.7%)	-13.80%
Use of a contraceptive injection can make a woman permanently infertile	47 (45.6%)	35 (34%)	-11.60%	123 (59.4%)	77 (37.2%)	-22.20%	108 (51.2%)	82 (38.9%)	-12.30%
Contraceptives reduce women's sexual urges	36 (35%)	28 (27.2%)	-7.80%	107 (51.7%)	76 (36.7%)	-15%	101 (47.9%)	65 (30.8%)	-17.10%
Contraceptives can give you deformed babies	44 (42.7%)	26 (25.2%)	-17.50%	106 (51.2%)	71 (34.3%)	-16.90%	98 (46.5%)	69 (32.7%)	-13.80%
After a woman uses contraceptive methods, it is difficult to get pregnant	47 (45.6%)	32 (31.1%)	-14.50%	102 (49.3%)	80 (38.7%)	-10.60%	92 (43.6%)	81 (38.4%)	-5.20%
Birth control should be a female concern	20 (19.4%)	16 (15.5%)	-3.90%	59 (28.5%)	35 (16.9%)	-11.60%	59 (28%)	41 (19.4%)	-8.60%
Women who use family planning / birth-spacing may become promiscuous	52 (50.5%)	42 (40.8%)	-9.70%	111 (53.6%)	89 (43%)	-10.60%	109 (51.7%)	92 (43.6%)	-8.10%
**Subject specific analysis**									
Average # myths believed, per participant (Std. Error)	***5.17***	***4.04***		***5.01***	***3.90***		***5.57***	***4.12***	
Average absolute change in myths believed	***-1.13***			***-0.98***			***-1.44***		
Conf. interval of the diff.	***[-1.59, -0.67]***			***[-1.57, -0.39]***			***[-1.91, -0.98]***		

Percentage absolute change in myths believed	**-21.9%**	**-20.2%**	**-25.9%**
Test of diff. in the mean of the absolute change in myths believed	Control (n=211)	Intervention (n=103)	Contact (n=207)
ANOVA test F statistics	***0.81***		
P-value	***0.4458***		

Suppl. Figure 1. The interventions each arm received

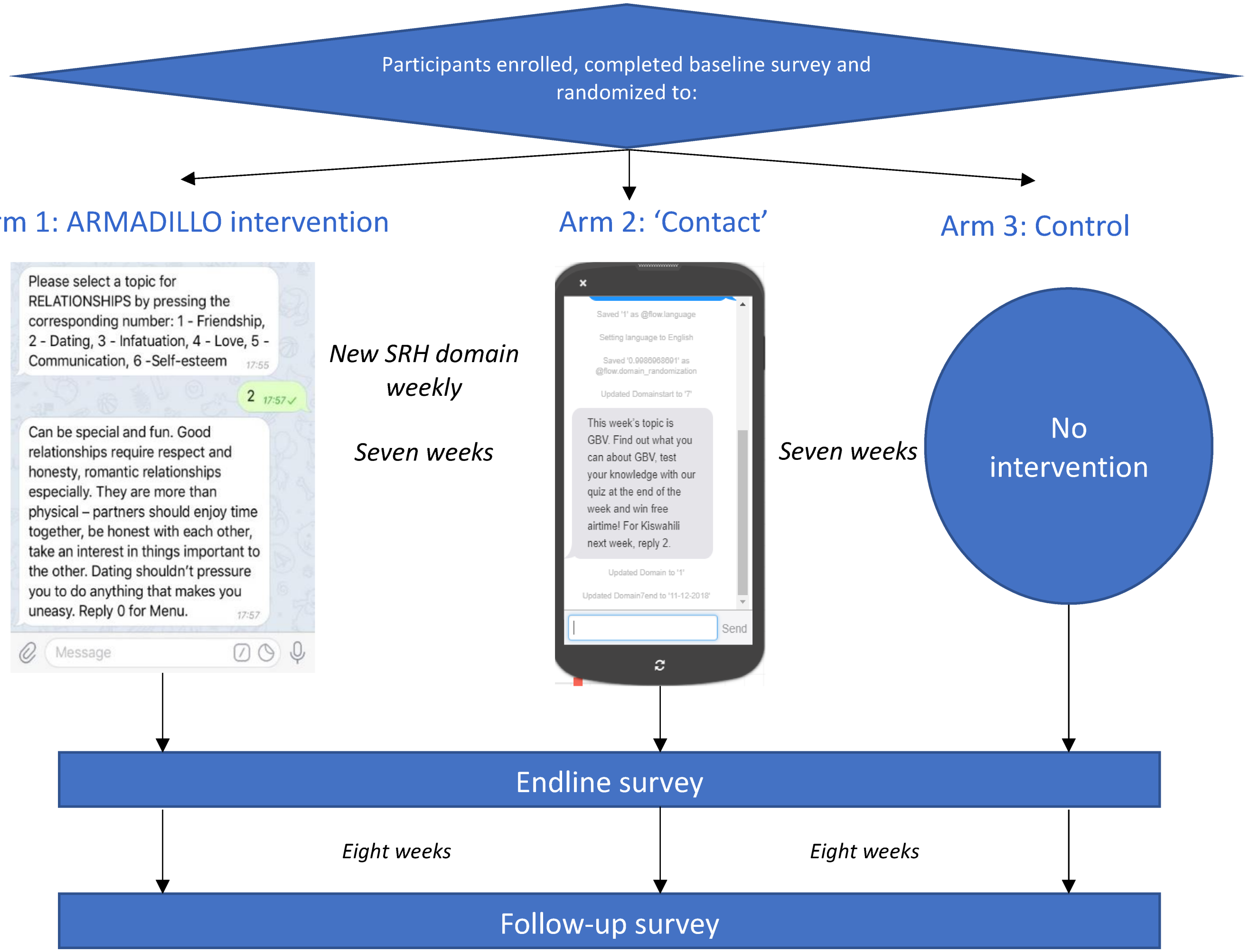

Suppl. Figure 2. The message domains which the intervention arms participants could request for more information and contacts arms participants studied on their own

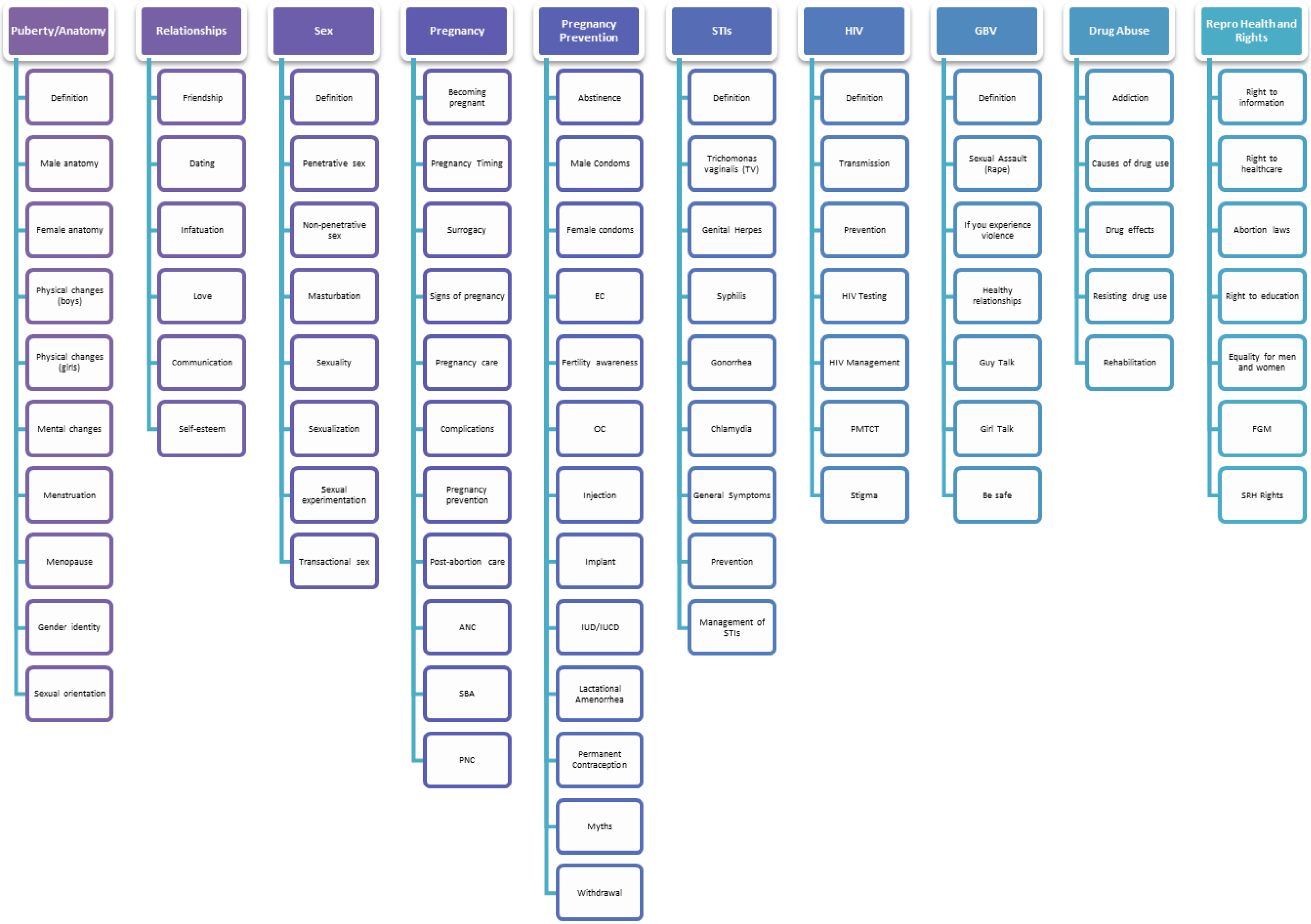

Gichangi P, *et al. BMJ Open* 2022; 12:e047426. doi: 10.1136/bmjopen-2020-047426
