# Supplementary material for: Busting contraception myths and misconceptions among youth in Kwale County, Kenya: results of a digital health randomised control trial
Source: BMJ Open. 2022 Jan 6;12(1):e047426. doi: 10.1136/bmjopen-2020-047426 (PMC8739061; doi:10.1136/bmjopen-2020-047426)
Supplement: Author's manuscript [file bmjopen-2020-047426.draft_revisions.pdf]

BMJ Open is committed to open peer review. As part of this commitment we make the peer review history of every article we publish publicly available.

When an article is published we post the peer reviewers' comments and the authors' responses online. We also post the versions of the paper that were used during peer review. These are the versions that the peer review comments apply to.

The versions of the paper that follow are the versions that were submitted during the peer review process. They are not the versions of record or the final published versions. They should not be cited or distributed as the published version of this manuscript.

BMJ Open is an open access journal and the full, final, typeset and author-corrected version of record of the manuscript is available on our site with no access controls, subscription charges or pay-per-view fees (http://bmjopen.bmj.com).

If you have any questions on BMJ Open's open peer review process please email info.bmjopen@bmj.com

BMJ Open

Bursting contraception myths and misconceptions among youth in Kwale County, Kenya: Results of a digital health randomized control trial

Journal:	*BMJ Open*
Manuscript ID	bmjopen-2020-047426
Article Type:	Original research
Date Submitted by the Author:	30-Nov-2020
Complete List of Authors:	Gichangi, Peter; Technical University of Mombasa, Administration; International Centre for Reproductive Health Kenya<br>Gonsalves, Lianne; World Health Organization, Department of Sexual and Reproductive Health and Research including UNDP/UNFPA/UNICEF/WHO/World Bank Special Programme of Research, Development and Research Training in Human Reproduction (HRP)<br>Mwaisaka, Jefferson; International Centre for Reproductive Health Kenya<br>Thiongo, Mary; International Centre for Reproductive Health Kenya<br>Habib, Ndema; World Health Organization, Department of Sexual and Reproductive Health and Research including UNDP/UNFPA/UNICEF/WHO/World Bank Special Programme of Research, Development and Research Training in Human Reproduction (HRP)<br>Waithaka, Michael; International Centre for Reproductive Health Kenya,<br>Tamrat, Tigest ; World Health Organization, Department of Sexual and Reproductive Health and Research including UNDP/UNFPA/UNICEF/WHO/World Bank Special Programme of Research, Development and Research Training in Human Reproduction (HRP)<br>Agwanda, Alfred ; University of Nairobi<br>Sidha, Hellen; National Council for Population and Development<br>Temmerman, Marleen; Aga Khan University - Kenya; International Centre for Reproductive Health<br>Say, Lale; World Health Organization, Department of Sexual and Reproductive Health and Research including UNDP/UNFPA/UNICEF/WHO/World Bank Special Programme of Research, Development and Research Training in Human Reproduction (HRP)
Keywords:	Reproductive medicine < GYNAECOLOGY, PUBLIC HEALTH, SEXUAL MEDICINE, Clinical trials < THERAPEUTICS

SCHOLARONE™
Manuscripts

I, the Submitting Author has the right to grant and does grant on behalf of all authors of the Work (as defined in the below author licence), an exclusive licence and/or a non-exclusive licence for contributions from authors who are: i) UK Crown employees; ii) where BMJ has agreed a CC-BY licence shall apply, and/or iii) in accordance with the terms applicable for US Federal Government officers or employees acting as part of their official duties; on a worldwide, perpetual, irrevocable, royalty-free basis to BMJ Publishing Group Ltd ("BMJ") its licensees and where the relevant Journal is co-owned by BMJ to the co-owners of the Journal, to publish the Work in this journal and any other BMJ products and to exploit all rights, as set out in our licence.

The Submitting Author accepts and understands that any supply made under these terms is made by BMJ to the Submitting Author unless you are acting as an employee on behalf of your employer or a postgraduate student of an affiliated institution which is paying any applicable article publishing charge ("APC") for Open Access articles. Where the Submitting Author wishes to make the Work available on an Open Access basis (and intends to pay the relevant APC), the terms of reuse of such Open Access shall be governed by a Creative Commons licence – details of these licences and which Creative Commons licence will apply to this Work are set out in our licence referred to above.

Other than as permitted in any relevant BMJ Author's Self Archiving Policies, I confirm this Work has not been accepted for publication elsewhere, is not being considered for publication elsewhere and does not duplicate material already published. I confirm all authors consent to publication of this Work and authorise the granting of this licence.

For peer review only

Bursting contraception myths and misconceptions among youth in Kwale County, Kenya: Results of a digital health randomized control trial

Gichangi Peter, PhD*[1,2,3], Gonsalves Lianne, PhD*[4,5,6], Mwaisaka Jefferson M. A[1,7], Thiongo Mary, MSC[1], Habib Ndema, PhD[4], Waithaka Michael, MSC[1], Tamrat Tigest, MPH[4], Agwanda Alfred, PhD[8], Sidha Hellen, MPH[9], Temmerman Marleen, PhD[1,3,10], Say Lale, MD[4].

***These authors have equal contribution**

[1]International Centre for Reproductive Health, Kenya; [2]Technical University of Mombasa; [3]Department of Public Health and Primary Care, Faculty of Medicine and Health Sciences, Ghent University, Belgium; [4]Department of Sexual and Reproductive Health and Research including UNDP/UNFPA/UNICEF/WHO/World Bank Special Programme of Research, Development and Research Training in Human Reproduction (HRP), World Health Organization, Geneva, Switzerland, [5]Swiss Tropical and Public Health Institute (Swiss TPH), Basel, Switzerland; [6]University of Basel, Basel, Switzerland [7]University of Ghana, College of Health Sciences, Accra Ghana; [8]University of Nairobi; [9]National Council for Population and Development; [10]Aga Khan University, Kenya

Corresponding author:

Prof Peter Gichangi, PhD
Address: P.O Box 2631-00202, Nairobi, Kenya,
email: gichangip@yahoo.com.
Phone: +254 722 521 946

Word Count (excluding abstract, tables, references)

3695

Abstract:

Objectives

The objective of this randomized controlled trial in Kenya was to assess the effect of delivering sexual and reproductive health (SRH) information via text message to young people on their ability to reject contraception-related myths and misconceptions.

Design and Setting

A three-arm, unblinded randomized controlled trial with a ratio of 1:1:1 in Kwale County, Kenya.

For peer review only

Participants and interventions

A total of 740 youth aged 18-24 years were randomized. Intervention arm participants could access ARMADILLO's SRH messages on-demand. Contact arm participants received once weekly texts instructing them to study on an SRH topic on their own. Control arm participants received standard care. The intervention period was 7 weeks.

Primary outcome

We assessed change myths believed at baseline and endline using an index of 10 contraception-related myths. We assessed change across arms using difference of difference analysis.

Results

Across arms, <5% of participants did not have any formal education, <10% were living alone, about 50% were single and >80% had never given birth. Between baseline and endline, the average numbers of myths and misconceptions believed decreased by 11.1%, 14.4% and 11.3% among intervention, contact and control arms respectively. However, we observed no statistically significant difference in the magnitude of change across arms.

Conclusions

Despite a significant decrease in myths-believed that we observed in the intervention arm, we are unable to conclusively state that the ARMADILLO intervention was better than SMS 'contact' or no intervention at all. Digital health likely has potential for improving SRH-related outcomes when used as part of multi-faceted interventions. Additional studies with physical and geographical separation of different arms is warranted.

Trial Registration

ISRCTN85156148

Strengths and limitations

- This study included two digital intervention arms, meaning that it would be possible to determine whether changes in outcomes were due to sexual and reproductive health content delivered by phone, or being 'nudged' by phone to think about (and learn about) their SRH.
- The study's intentionally did not power sample size around SRH behavioural outcomes, building on previous research that light-touch digital interventions alone may not be enough to see behaviour change in such a complex area of health – instead the primary outcome focuses on SRH knowledge.
- A key limitation is that the study's individual-level randomization of young people living near each other is likely to have resulted in contamination between arms.

For peer review only - http://bmjopen.bmj.com/site/about/guidelines.xhtml

Introduction

There is a high unmet need for sexual and reproductive health (SRH) information and services, for both married and unmarried youth worldwide. Data from 61 low and middle-income countries (LMICs) show that 33 million women aged 15–24 have an unmet need for contraception. [1] In Kenya, the 2014 Demographic and Health Survey found that modern contraceptive use among all adolescents age 15-19 years is low (9.3%) compared to all women aged 15-49 years (39.1%). [2] Partly as a result, the number of adolescents aged 15-19 years who were pregnant or mothers has stagnated at 18% between 2008 and 2014. [2, 3]

Sexually active young people face a variety of obstacles to access and use modern contraceptives. They may encounter financial, cultural, social, legal barriers, fear of side effects (e.g. infertility and adverse reactions) or cultural norms that restrict their access to contraception services in health facilities. [4-7] Additionally, contraception myths and misconceptions can negatively affect access to and use of SRH services. [8, 9] Misinformation, side effects concerns and myths/misconceptions are often learnt from social networks. [10, 11]

The proliferation of mobile phone technology, and its popularity and ownership with young people in particular, [12, 13] provides an innovative way to educate young people on contraception and their health more broadly. There are indications that health promotion campaigns among adolescents and young people through text messaging may contribute to improved sexual and reproductive health knowledge, behaviours and outcomes. [14] However, there is less rigorous research and documentation of SRH mobile phone interventions for adolescents and young people in developing countries. [15] In Kenya in particular, an estimated 93% of households already owned a mobile phone by 2011. [16]

For peer review only - http://bmjopen.bmj.com/site/about/guidelines.xhtml

Mobile phone-based digital health interventions have been successfully used in HIV programs [17, 18] and to address chronic disease conditions. [19] Providing broader SRH content, including contraception information, via mobile phones to young people would appear to be a natural strategy to reach them [20] and increase their contraception knowledge. [21] After all, when it comes to 'sensitive' SRH content, mobile phones can privately deliver information without stigma or judgment. However, the evidence that digital health interventions can improve youth SRH-related outcomes, including contraception knowledge and uptake, is yet to be significantly established. [22-24]

To address this gap, the World Health Organization's Department of Reproductive Health and Research partnered with research partners in Peru and Kenya to develop the Adolescent/Youth Reproductive Mobile Access and Delivery Initiative for Love and Life Outcomes (ARMADILLO) Study. The ARMADILLO intervention used short message service (SMS, also known as 'text message') to deliver SRH information on-demand via a number-based menu. The content was developed in the study's formative stage around several SRH 'domains' of interest to policymakers and young people alike. [25] The intervention was evaluated using a three-arm randomized controlled trial (RCT). This paper reports on the study's primary outcome: are young people with access to the ARMADILLO intervention better able to reject contraception myths and misconceptions as compared with periodic SMS encouraging self-learning or usual care (no intervention) for Kenya only.

For peer review only

Methods

Study Design

This was a three-arm randomized controlled trial (1:1:1 allocation) involving youth age 18-24 years. The trial ran for seven weeks, with assessments at baseline and endline. The study methods have been described elsewhere in full, [26] but are described briefly below.

For peer review only - http://bmjopen.bmj.com/site/about/guidelines.xhtml

Participants and Setting

The study was conducted in Kwale County, one of the six counties in the Coastal region of Kenya. The study area consisted of select enumeration areas (EAs) in six Kwale County sub-locations which border each other: Ngombeni, Kitivo, Simkumbe, Mkoyo, Gombato and Ukunda. Eligibility criteria was as follows: youth (male and female) aged 18-24; literate; had their own mobile phone at the time of recruitment and reported regular use; reported current use of text messaging.

The Kenya National Bureau of Statistics provided a list of EAs for the six sub-locations. From this list, we randomly selected 21 EAs to be mapped. During the mapping (October 2017), data collectors visited all households and enumerated anyone in the home who was age-eligible to participate. Then, we randomly selected one eligible participant from each household. Starting in February 2018, trained data collectors returned to households and attempted to recruit the selected youth. After consenting to participate, participants completed a baseline survey. Both baseline and endline surveys were implemented by trained data collectors, who entered participants' responses into a webform on a tablet. For a few sensitive questions relating to previous contraception use and sexual behaviour, participants entered their responses onto the tablet themselves. Participants were then remotely randomized into one of three arms. Intervention and contact arm participants received their first message the following day.

For peer review only

Interventions

The interventions were categorized as per WHO classification of digital health interventions. [27] Arm 1 (Intervention arm) was an *on-demand information service to clients (WHO Classification 1.6)*. Participants received access to one domain of ARMADILLO content (e.g. 'puberty and anatomy' or 'pregnancy prevention') each week and could request any 'sub-domain' that interested them from the menu (e.g. 'menstruation' or 'physical changes' for the puberty and anatomy domain or 'implants' or 'male condoms' for the pregnancy prevention domain) for free for the entire week (see Suppl Figure

For peer review only - http://bmjopen.bmj.com/site/about/guidelines.xhtml

1). Arm 2, (Contact arm) employed *targeted client communication (WHO Classification 1.1)*. Participants received the same system-initiated contacts as Arm 1 participants but without access to the ARMADILLO content itself. Instead, a once-weekly SMS alerted them to an SRH domain for that week (e.g. relationships, pregnancy, HIV) and encouraged them to learn on their own (see Suppl Figure 2). At the end of the week, participants in both arms were provided with a single SMS-based quiz question on that domain's content. If the participant answered (correctly or not), they received a small amount of airtime (1USD). Intervention and contact messages were available in either Swahili or English, per the participant's preference. All SMS costs were reverse-billed to the study, so intervention and contact arm participants incurred no costs from their participation. Those randomized to Arm 3 (Control arm) received standard of care (no messages).

Assessments and Outcome

The primary outcome was assessed using an index developed by the research team of 10 contraception myths and misconceptions (Box 1). These were identified based on literature review and a series of focus group discussions with young people prior to the start of the RCT. At baseline and endline, RCT participants were asked to state how much they agreed or disagreed with a given statement based on a four-point Likert scale.

Box 1. Myths and misconceptions

Health - People who use contraceptives end up with health problems;

Body shape - Hormonal contraceptives are fattening;

Infertility – (1) After a woman uses contraceptive methods, it is difficult to get pregnant, and (2) use of a contraceptive injection can make a woman permanently infertile;

Harm - Contraceptives can harm a woman's womb;

Sex drive - Contraceptives reduce women's sexual urges;

Cancer - Contraceptives can cause cancer;

For peer review only - http://bmjopen.bmj.com/site/about/guidelines.xhtml
For peer review only

Malformations - Contraceptives can give you deformed babies; and

Social constructs – (1) Birth control should be a female concern; and (2) women who use family planning/birth-spacing may become promiscuous.

Sample Size

The sample size was calculated such that it provided 80% power to detect a 10% change in mean number of myths believed from baseline to endline, assuming that baseline level of belief was 55%, Type 1 error at 5% using two-sided Z test with continuity correction and unpooled variance and accounting for a dropout rate of up to 20%. The sample size accounted for Bonferroni correction due to 3-arm pairwise comparisons. Based on the aforementioned, a minimum number of participants to be sampled was 705, split evenly across intervention, contact, and control arms (1:1:1).

Randomization procedures

Participants were individually randomized to either intervention, contact or control group using a ratio of 1:1:1 as per computer-generated randomization schedule developed using Node.js and docker. All the study participants had equal probability of being assigned to either arm. Allocation took place after the participant had completed the baseline survey. ARMADILLO was an open-label trial; however, neither the technological partner nor the research team had any control of arm assignments.

Data analysis

The 10 items of the primary outcome were dichotomized from the original Likert scale (Strongly agree, Agree, Disagree and Strongly disagree) as follows: 1) Agree and Strongly Agree [participant believed the myth – bad] were recoded as Agree and coded as 1; 2) Disagree and Strongly Disagree [participant rejected the myth – good] were recoded as Disagree and coded as 0. A participant score for the 10 questions was generated with a total maximum score of 10, corresponding to the number of myths/misconceptions that the participant believed. The average number of myths/misconceptions

For peer review only

believed by participants in each arm was computed. There were no missing values for the 10 items across all arms. The study participants responded to all the 10 primary outcome questions in the assessment.

The baseline factors were described using proportions. To ensure that oversampling in certain arms had no effect on the randomization, we performed χ^2 tests on demographic characteristics to confirm that there were no baseline differences between arms. To assess attrition bias (a systematic error caused by unequal loss of participants from the trial between the baseline and the endline), we used Fisher's exact χ^2 tests for the socio-demographic variables to test whether participants lost to follow up differed across the trial arms (Suppl Table 1) as well as if they differed from those who responded as a function of study group (Suppl Table 2).

First, we present proportions of those who believed in the myths at baseline and endline for all arms and the percentage change in the myths believed between the two periods. To obtain the average number of myths believed per participant, we computed *(using the sum of the dichotomized 10-item response)* the number of myths believed for each participant at baseline and at endline. Then for each participant, we computed the average number of myths believed (expressed as a percentage) at the baseline and at endline (by dividing the sum of myths believed by 10 and multiplying by 100). Next, for each participant, his or her absolute change in the average myths believed between the endline and baseline was computed (endline minus baseline).

Normality of the absolute changes in the myths believed was tested using the quantile-quantile plots. As the distribution of the absolute changes in the myths believed was normally distributed, Analysis of Variance (ANOVA) test of equality of group means was used to test the between group differences in the means of the absolute myths change. We estimated the difference-in-differences (DID) of the average number of myths believed by participants in a given arm to evaluate the effect of the

For peer review only - http://bmjopen.bmj.com/site/about/guidelines.xhtml

ARMADILLO intervention to dispel myths and misconceptions about contraception. DID tells us whether the expected mean change in the number of myths and misconceptions believed from baseline to endline was different in the groups compared. DID is calculated by subtracting the average of the outcome in the control or contact arm from the average of the outcome in the intervention arm ($\hat{d}_1$), where the outcome is the change in percentage number of myths believed by each individual between the endline and baseline. DID was also used to assess changes in the average proportion of myths believed per participant between the control and the contact arm ($\hat{d}_2$).

For peer review only

$$\hat{d}_1 = \{[Mean(Y_i(Endline)) - Mean(Y_i(Baseline))]_{Intervention}\}\{[Mean(Y_i(Endline)) - Mean(Y_i(Baseline))]_j\}$$

$$\hat{d}_2 = \{[Mean(Y_i(Endline)) - Mean(Y_i(Baseline))]_{Contact}\} - \{[Mean(Y_i(Endline)) - Mean(Y_i(Baseline))]_{Control}\}$$

$$Where\ \boldsymbol{i}\ refer\ to\ the\ ith\ individual\ in\ the\ trial\ arm;\ while\ \boldsymbol{j} = Contact\ or\ control$$

All analyses were based on Complete Case (CC) dataset while analyses based on per protocol (PP) dataset were used for sensitivity analysis. Participants were included in the analysis provided that they had completed both baseline and endline surveys. In this case, the ITT analysis was equivalent to the CC.

Participants were included in the per protocol analysis provided that they had completed baseline and endline surveys, *and* that the intervention system could confirm that they had 1) received the ARMADILLO message domain associated with the primary outcome (Pregnancy Prevention); and 2) requested at least one message from this domain. Results with a Type I error of $p<0.05$ in two-sided tests were considered statistically significant. Where Bonferroni correction was applied for the pairwise

For peer review only - http://bmjopen.bmj.com/site/about/guidelines.xhtml

comparisons of the three study arms, p<0.017 were considered statistically significant. Analyses were performed using Stata version 15.

Patients and public involvement statement

ARMADILLO's population of 'young people' were involved in the study from its initial, formative stage[25, 28], which included message content development. They and the broader community continued engagement through this trial through the ARMADILLO Community Advisory Board (CAB) consisting of representatives from the Ministries of Health, Education, and Social Services; youth-led organizations; area chiefs; health care workers providing SRH services to adolescents; and young people themselves. CAB members provided technical and field support throughout the data collection period. Additionally, young people identified from the study area were trained as data collectors and hired to enumerate young people in the area as well as recruit and implement baseline and endline surveys with study participants. Young data collectors' input also shaped recruitment hours and strategies. A dissemination involving local and national stakeholders took place in July 2019 – selected young data collectors participated in the dissemination meeting and shared their feedback.

Ethical considerations

The study was approved by the ethics committees at the Kenyatta National Hospital (KNH ERC P550/09/2014) and the World Health Organization (Protocol WHO A65892). This trial is registered with the ISRCTN Registry (ISRCTN85156148).

Results

A total of 740 men and women aged 18-24 years were randomized into intervention, contact and control arms (Figure 1).

Figure 1 ARMADILLO Peru's Consolidated Standards of Reporting Trials (CONSORT) diagram

For peer review only - http://bmjopen.bmj.com/site/about/guidelines.xhtml

In the intervention period which lasted 7 weeks, 116 of the 740 (16%) study participants dropped out over the course of the seven-week intervention period (Intervention arm – 49 (19%); Contact arm – 42 (17%); Control arm – 25 (11%)). Among participants in the intervention arm, 206 (81%) completed both the baseline and the endline assessments (making them eligible for CC analysis) while 103 (40%) were eligible for per protocol (PP) analysis. Among the contact group, 207 (83%) received the push messages and were included in both the CC and PP analysis. In the control group, 211 (89.4%) completed both the baseline and the endline assessments. Baseline characteristics for the study sample are shown in Table 1. Across all arms, 53% of the participants were male, 48% had a secondary education or higher, 65% were from Ukunda sub-location, 91% lived with others, and 85% did not have children. There were no significant baseline differences between the intervention, contact and control groups.

For peer review only

For peer review only - http://bmjopen.bmj.com/site/about/guidelines.xhtml

Table 1: Baseline characteristics of the participants, by study arm (N = 740)

Characteristic	Intervention (I)	Contact (P)	Control (C)	Total
	N=255, n (%)	N=249, n (%)	N=236, n (%)	N=740, n (%)
**Age of the participant**				
18 – 19 years	62 (24.3)	55 (22.09)	53 (22.5)	170 (23.0)
20 – 24 years	193 (75.7)	194 (77.91)	183 (77.5)	570 (77.0)
**Sex**				
Male	134 (52.6)	133 (53.41)	126 (53.4)	393 (53.1)
Female	121 (47.5)	116 (46.59)	110 (46.6)	347 (46.9)
**Education level**				
Never gone to school	9 (3.5)	7 (2.81)	12 (5.1)	28 (3.8)
Primary school	92 (36.1)	97 (38.96)	80 (33.9)	269 (36.4)
Secondary school	117 (45.9)	118 (47.39)	119 (50.4)	354 (47.8)
Post-secondary education	37 (14.5)	27 (10.84)	25 (10.6)	89 (12.1)
**Sub-location**				
Ngombeni	34 (13.3)	38 (15.26)	43 (18.2)	115 (15.5)
Kitivo	9 (3.5)	9 (3.61)	9 (3.8)	27 (3.7)
Simkumbe	20 (7.8)	17 (6.83)	21 (8.9)	58 (7.8)
Mkoyo	8 (3.1)	8 (3.21)	8 (3.4)	24 (3.2)
Gombato	17 (6.7)	11 (4.42)	9 (3.8)	37 (5.0)
Ukunda	167 (65.5)	166 (66.67)	146 (61.9)	479 (64.7)
**Person currently living with**				
Living alone	24 (9.4)	21 (8.43)	21 (8.9)	66 (8.9)
Living with others	231 (90.6)	228 (91.57)	215 (91.1)	674 (91.1)
**Current relationship status**				
Single	128 (50.2)	121 (48.59)	118 (50.0)	367 (49.6)
Friends with benefits/ Dating/Cohabiting/ Engaged	109 (42.8)	104 (41.77)	92 (39.0)	305 (41.2)
Married	18 (7.1)	24 (9.64)	26 (11.0)	68 (9.2)
**Parity**				
None	224 (87.8)	211 (84.7)	191 (80.9)	626 (84.6)
1 child	24 (9.4)	28 (11.2)	35 (14.8)	87 (11.8)
2+ children	7 (2.8)	10 (4.0)	10 (4.2)	27 (3.7)
**First birth age**				
Never given birth	224 (87.8)	211 (84.7)	191 (80.9)	626 (84.6)
<=19 years (Adolescents)	18 (7.1)	22 (8.8)	23 (9.8)	63 (8.5)
>=20 years (Young women)	13 (5.1)	16 (6.4)	18 (7.6)	47 (6.4)

For peer review only - http://bmjopen.bmj.com/site/about/guidelines.xhtml

Concerning attrition bias, the analysis revealed that participants who dropped out in each arm were similar to each other (Suppl Table 1). However, there was a significant association between dropping out of the study and the number of children the participant had at the time of the study in the control and the contact groups' participants, Suppl. table1. The analysis assessing attrition bias also revealed that there was no significant difference in the socio-demographics between the participants who were lost to follow up and those who finished the seven weeks of the intervention and took the endline survey, Suppl. table 2.

The results of the CC analysis examining the myths and misconceptions believed are displayed in Table 2. The myths are ordered by those which were believed by the most number of participants, across groups, with the most salient myth at the top. The results show that at baseline, study participants in all arms believed around half of the myths related to contraception on average. At the end of the seven-week intervention period, the average number of myths and misconceptions believed per participant had significantly decreased for all the three groups ($p<0.0001$). The average absolute decrease in the myths believed was 11.1% among the intervention group (mean -11.1%; 95% CI [-17.1%,-5.2%]); 14.4% among the contact group (mean -14.4%; 95% CI [-20.5%,-8.4%]) and 11.3% among the control group (mean -11.3%; 95% CI [-17.4%,-5.2%]). From the normality test shown in Figure 2, the absolute change in all the three groups were normally distributed. The ANOVA test of equality-of-populations means showed that there was no significant difference in the group medians ($p=0.5181$).

Figure 2: Checking the normality of the absolute changes in the myths believed

For peer review only - http://bmjopen.bmj.com/site/about/guidelines.xhtml

Table 2: Intervention Effects for Dichotomous Outcomes – Complete Case Analysis

Myth	Control (n=211) Baseline	Endline	Diff.	Intervention (n=206) Baseline	Endline	Diff.	Contact (n=207) Baseline	Endline	Diff.
**Population based analysis**									
Hormonal contraceptives are fattening	139 (65.9%)	117 (55.5%)	-10.4%	144 (69.9%)	135 (65.5%)	-4.4%	142 (68.6%)	127 (61.4%)	-7.2%
Contraceptives can harm a woman's womb	135 (64%)	114 (54%)	-10%	125 (60.7%)	106 (51.5%)	-9.2%	144 (69.6%)	112 (54.1%)	-15.5%
People who use contraceptives end up with health problems	133 (63%)	103 (48.8%)	-14.2%	120 (58.3%)	105 (51%)	-7.3%	135 (65.2%)	100 (48.3%)	-16.9%
Contraceptives can cause cancer	117 (55.5%)	88 (41.7%)	-13.8%	112 (54.4%)	90 (43.7%)	-10.7%	123 (59.4%)	86 (41.6%)	-17.8%
Use of a contraceptive injection can make a woman permanently infertile	108 (51.2%)	82 (38.9%)	-12.3%	102 (49.5%)	73 (35.4%)	-14.1%	123 (59.4%)	77 (37.2%)	-22.2%
Contraceptives reduce women's sexual urges	101 (47.9%)	65 (30.8%)	-17.1%	83 (40.3%)	57 (27.7%)	-12.6%	107 (51.7%)	76 (36.7%)	-15%
Contraceptives can give you deformed babies	98 (46.5%)	69 (32.7%)	-13.8%	92 (44.7%)	53 (25.7%)	-19%	106 (51.2%)	71 (34.3%)	-16.9%
After a woman uses contraceptive methods, it is difficult to get pregnant	92 (43.6%)	81 (38.4%)	-5.2%	101 (49%)	69 (33.5%)	-15.5%	102 (49.3%)	80 (38.7%)	-10.6%
Birth control should be a female concern	59 (28%)	41 (19.4%)	-8.6%	46 (22.3%)	32 (15.5%)	-6.8%	59 (28.5%)	35 (16.9%)	-11.6%
Women who use family planning / birth-spacing may become promiscuous	109 (51.7%)	92 (43.6%)	-8.1%	108 (52.4%)	84 (40.8%)	-11.6%	111 (53.6%)	89 (43%)	-10.6%
**Subject specific analysis**									
Average # myths believed, per participant (Std. Error)	***5.17***	***4.04***		***5.01***	***3.90***		***5.57***	***4.12***	
Average absolute change in myths believed	***-1.13***			***-1.11***			***-1.44***		
Conf. interval of the diff.	***[-1.59, -0.67]***			***[-1.54, -0.68]***			***[-1.91, -0.98]***		
Percentage absolute change in myths believed	***-21.9%***			***-22.2%***			***-25.9%***		
Test of diff. in the mean of the absolute change in myths believed	Control (n=211)			Intervention (n=206)			Contact (n=207)		
ANOVA test F statistics	***0.66***								
P-value	***0.5181***								

n* – Those who believed the myth (had wrong answer);

For peer review only - http://bmjopen.bmj.com/site/about/guidelines.xhtml

As presented in the difference-in-difference analysis in Table 3, there was no statistically significant differences between the baseline to endline decrease observed across the 3-arms (p>0.017-Bonferroni corrected significance level).

Table 3: Difference in difference analysis

Outcome	Percentage point differences (95%CI)	p-value
Contraception myths and misconceptions index score (endline – baseline assessment)		
Arm 1: Intervention (MeanΔ, 95%CI)	-11.1% (-17.1%,-5.2%)	<0.001
Arm 2: Contact (MeanΔ, 95%CI)	-14.4% (-20.5%,-8.4%)	<0.001
Arm 3: Control (MeanΔ, 95%CI)	-11.3% (-17.4%,-5.2%)	<0.001
Mean (Δ Intervention) - Mean (Δ Control)	0.2% (-8.3%, 8.7%)	0.961
Mean (Δ Contact) - Mean (Δ Control)	-3.1% (-11.7%, 5.4%)	0.475
Mean (Δ Intervention) - Mean (Δ Contact)	3.3% (-5.1%, 11.8%)	0.440

Notes: Δ refers to the subject-specific change in the outcome from baseline to endline. 95%CI refers to the 95% confidence interval. A generalized linear model (GLM) using a normal distribution and identity link was used to compare scores. Effective sample size used for the analysis was 206 participants for the intervention group; 207 participants for the contact group and 211 participants for the control group.

Sensitivity analysis including only participants who met PP inclusion criteria did not alter the findings from CC analysis. As the results of the CC analysis, and PP analysis did not differ with respect to statistical significance within groups or between groups, only those for the CC analysis are reported (PP analysis is attached as supplementary materials – Suppl. table 3).

Finally, Table 4 shows estimates of a possible source of contamination between study arms. Of the 23% of the participants in the intervention group who shared the messages with others, 13%, 5%, and 4% shared the messages with friends, partners, and multiple contacts, respectively. Among the 27% contact group participants who shared the messages with others, 15%, 8%, 1% and 3% shared the messages with friends, partners, siblings and multiple contacts, respectively.

For peer review only

Table 4: Study contamination

		Shared the messages	Shared the messages with: Siblings	Friends	Partner	Parents	multiple contacts
Intervention (N=206)	n	47	0	26	10	2	9
	%	22.8%	0.0%	12.6%	4.9%	1.0%	4.4%
Contact (N=207)	n	55	2	30	16	0	7
	%	26.6%	1.0%	14.5%	7.7%	0.0%	3.4%
Total (N=413)	n	102	2	56	26	2	16
	%	24.7%	0.5%	13.6%	6.3%	0.5%	3.9%

Discussion

Our findings suggest that provision of sexual and reproductive health content via SMS is potentially useful in dealing with contraception-related myths and misconceptions among youth. Across arms, the study demonstrated between a 11% and 14% reduction in the average number of myths/misconception statements believed over the study period. However, we did not observe a significant difference in the magnitude of reduction between the arms. Therefore, despite the significant decrease in myths-believed that we observed between baseline and endline, we are unable to conclusively state that the ARMADILLO intervention was better than SMS 'contact' or no intervention at all.

One possible reason for not seeing a significant effect of the ARMADILLO intervention versus no intervention is that correcting false information is difficult. Studies aimed at correcting misinformation about vaccines, for example, have shown that even when attempts to correct invalid information do not entrench the original misinformation, they can frequently fail because people cannot successfully update their memories, and still fall back on information they know is not correct. [29-32] A seven-week digital health intervention, dedicated to sexual and reproductive health broadly, may not have been enough to dispel deeply-entrenched concerns about contraception.

For peer review only - http://bmjopen.bmj.com/site/about/guidelines.xhtml

Alternatively, we may have seen no difference between the arms because the intervention was truly not better than SMS-prompted self-learning and/or no intervention at all. Several other RCTs that have attempted to tie adolescent-targeted digital health interventions to sexual and reproductive health (SRH) knowledge or behavioural outcomes have resulted in similarly inconclusive findings, indicating that digital interventions *on their own* may not be enough to encourage behaviour change. [23, 33, 34] However, while the above reasons would explain the lack of difference between arms, they do not explain why participants in all arms believed significantly fewer myths at endline than they did at baseline.

Here, contamination between arms may be to blame. Randomized controlled trials of digital health client communication interventions are difficult, especially when the intervention content can be easily shared between neighbours and across communities. To the best of our knowledge, there were no other health campaigns or interventions aimed at dispelling the myths and misconceptions in the area during the study period. However, about a quarter of the intervention and contact arm participants reported sharing information with study participants and other members of the community. Any effect of participants sharing information was amplified by our participants being randomized at the individual level. This resulted in participants in different arms living in close proximity, often in neighbouring households. ARMADILLO intervention arm participants may therefore have received the messages and shared them with their friends/neighbours, some of whom were ARMADILLO contact and control arm participants.

Addressing/dispelling myths and misconceptions among youth is particularly important for future contraception use. By nature of originating in social networks as well as their likelihood to "stick" indefinitely, myths and misconceptions among youth should be dispelled early to prevent their becoming engrained. [6, 35] Indeed, our study found that two of the three most commonly-believed

For peer review only - http://bmjopen.bmj.com/site/about/guidelines.xhtml

myths and misconceptions among youth age 18-24 years (people who use contraceptives end up with health problems, and contraceptives can harm a woman's womb) were also the top myths for youth and adult women aged 15-49 years in Kenya, Nigeria and Senegal. [9]

Other studies have reported successfully addressing myths and misconceptions with dedicated community and communication interventions, [36] involving a variety of opinion leaders and channels. [9, 36] Following a 4-year intervention using radio, religious leaders, and community health volunteers, for example, Kenya's Tupange study reported a 15% decrease in the number of women who believed myths and misconceptions statements between baseline and endline. [36]

Digital interventions are an additional channel which can be included in this mix. There is a wealth of opportunity to engage with young people en masse not only through SMS and voice channels, but also by widely-used messaging and social media platforms like WhatsApp, Facebook, and Facebook Messenger, the latter of which don't have the bulk telecom-related costs of SMS and voice interventions. The popularity of the ARMADILLO interventions among its users indicate that such interventions can be considered as one tool *in a multi-pronged approach* targeting young people with correct SRH information. [22, 37-39] However, evidence continues to be needed on adolescent-targeted client communication interventions in general.

This study is not without limitations. The lack of differences between the intervention and the other arms could very well be due to our adoption of individually-randomized rather than cluster-randomized study design. A cluster design was considered; however, the accessibility and make-up of the study area did not allow for homogenous clusters to be created (Ukunda, for example, is unique in Kwale County for its population density). While unfortunate for the results of the trial, it demonstrates that the messages were as popular as the research team hoped and provides some positive insight as to the dissemination which might take place if a similar intervention is implemented outside of a

For peer review only - http://bmjopen.bmj.com/site/about/guidelines.xhtml

research setting. The study is strengthened by its choice of primary outcome: we intentionally avoided powering the study around SRH behavioural outcomes, building on learnings from previous studies that light-touch digital interventions alone may not be enough to see behaviour change in such a complex area of health.

In conclusion, creative and consistent interventions are needed to address deeply-rooted myths and misconceptions young people have about contraception and mitigate one important driver of anxiety around contraception use in Kenya. These can include digital interventions. However, while the ARMADILLO study saw some promising results, we cannot conclusively say that digital interventions *alone* are sufficient to affect change in SRH-related outcomes. Additional research (using alternative designs) will be required to identify the specific value of digital targeted client communication programs in addressing young people's contraception myths/misconceptions and improving sexual and reproductive health knowledge overall.

Acknowledgments

The authors thank the youth in Kwale who participated in the study, the research assistants who collected data, and Winnie Wangari who coordinated and supervised data collection. The authors are grateful for feedback given by Carol Mukira and Nelly Ibeere.

Funding Statement

This work was funded by the UNDP/UNFPA/UNICEF/WHO/World Bank Special Programme of Research, Development and Research Training in Human Reproduction (HRP).

Data Availability Statement

Deidentified data related to this article can be obtained by request to the corresponding author.

For peer review only - http://bmjopen.bmj.com/site/about/guidelines.xhtml

Author Contributorship

P.G. and L.G. contributed equally to this paper (in study design, interpretation of data and writing of the manuscript). M.J, M.T, N.H, M.T and L.S. contributed to the design of the ARMADILLO study. M.J. contributed to data collection; M.W. and M.T. contributed to the analysis; P.G, L.G, M.J, M.T, N.H, H.S, A.A, M.T and L.S contributed to interpretation of data. P.G and L.G wrote the first draft of the paper, and all authors contributed to its revision; all authors approve of the submitted article. The manuscript represents the views of the named authors only.

References

1. MacQuarrie KLD. Unmet Need for Family Planning among Young Women: Levels and Trends. Rockville, Maryland, USA: ICF International; 2014.
2. Kenya National Bureau of Statistics. Kenya Demographic and Health Survey 2014. Rockville, Maryland: The DHS Program, ICF International; 2015.
3. Kenya National Bureau of Statistics (KNBS). Kenya Demographic and Health Survey 2008-09. Calverton, Maryland: KNBS and ICF Macro; 2010.
4. Chandra-Mouli VMM, D.R. ; Phillips, S.J.; Williamson, N.E.; Hainsworth, G. . Contraception for adolescents in low and middle income countries: needs, barriers, and access. Reproductive Health. 2014;11(1).
5. Denno DMH, Andrea J; Chandra-Mouli, Venkatraman. Effective Strategies to Provide Adolescent Sexual and Reproductive Health Services and to Increase Demand and Community Support. Journal of Adolescent Health. 2015;56(1):S22-S41.
6. Speizer IS, Calhoun LM, Guilkey DK. Reaching Urban Female Adolescents at Key Points of Sexual and Reproductive Health Transitions: Evidence from a Longitudinal Study from Kenya. African journal of reproductive health. 2018;22(1):47-59.
7. Parker JJ, Veldhuis CB, Hughes TL, Haider S. Barriers to contraceptive use among adolescents in two semi-rural Nicaraguan communities. International journal of adolescent medicine and health. 2019;32(5).
8. M AAAJO. Myths, misinformation, and communication about family planning and contraceptive use in Nigeria. Open Access Journal of Contraception. 2011;2:95-105.
9. Gueye A, Speizer IS, Corroon M, Okigbo CC. Belief in Family Planning Myths at the Individual and Community Levels and Modern Contraceptive Use in Urban Africa. International perspectives on sexual and reproductive health. 2015;41(4):191-9.
10. Paz Soldan VA. How family planning ideas are spread within social groups in rural Malawi. Studies in family planning. 2004;35(4):275-90.
11. Yee L, Simon M. The role of the social network in contraceptive decision-making among young, African American and Latina women. The Journal of adolescent health : official publication of the Society for Adolescent Medicine. 2010;47(4):374-80.
12. Hightow-Weidman LB, Muessig KE, Bauermeister J, Zhang C, LeGrand S. Youth, Technology, and HIV: Recent Advances and Future Directions. Curr HIV/AIDS Rep. 2015;12(4):500-15.
13. World Bank. The Little Data Book on Information and Communication Technology 2014. Washington DC: World Bank; 2014.
14. L'Engle KL, Mangone ER, Parcesepe AM, Agarwal S, Ippoliti NB. Mobile Phone Interventions for Adolescent Sexual and Reproductive Health: A Systematic Review. Pediatrics. 2016;138(3).
15. Ippoliti NB, L'Engle K. Meet us on the phone: mobile phone programs for adolescent sexual and reproductive health in low-to-middle income countries. Reprod Health. 2017;14(1):11.

For peer review only

For peer review only - http://bmjopen.bmj.com/site/about/guidelines.xhtml

16. Demombynes GT, Aaron. Kenya's Mobile Revolution and the Promise of Mobile Savings. World Bank Group; 2012.
17. Kalichman SC, Cherry C, Kalichman MO, Eaton LA, Kohler JJ, Montero C, et al. Mobile Health Intervention to Reduce HIV Transmission: A Randomized Trial of Behaviorally Enhanced HIV Treatment as Prevention (B-TasP). J Acquir Immune Defic Syndr. 2018;78(1):34-42.
18. Widman L, Nesi J, Kamke K, Choukas-Bradley S, Stewart JL. Technology-Based Interventions to Reduce Sexually Transmitted Infections and Unintended Pregnancy Among Youth. The Journal of adolescent health : official publication of the Society for Adolescent Medicine. 2018;62(6):651-60.
19. Anderberg P, Barnestein-Fonseca P, Guzman-Parra J, Garolera M, Quintana M, Mayoral-Cleries F, et al. The Effects of the Digital Platform Support Monitoring and Reminder Technology for Mild Dementia (SMART4MD) for People With Mild Cognitive Impairment and Their Informal Carers: Protocol for a Pilot Randomized Controlled Trial. JMIR research protocols. 2019;8(6):e13711.
20. Vahdat HL, L'Engle KL, Plourde KF, Magaria L, Olawo A. There are some questions you may not ask in a clinic: Providing contraception information to young people in Kenya using SMS. International Journal of Gynecology & Obstetrics. 2013;123(S1):e2-e6.
21. Rokicki S, Fink G. Assessing the reach and effectiveness of mHealth: evidence from a reproductive health program for adolescent girls in Ghana. BMC Public Health. 2017;17(1):969.
22. Garofalo R, Kuhns LM, Hotton A, Johnson A, Muldoon A, Rice D. A Randomized Controlled Trial of Personalized Text Message Reminders to Promote Medication Adherence Among HIV-Positive Adolescents and Young Adults. AIDS and behavior. 2016;20(5):1049-59.
23. Lim MSC, Hocking JS, Aitken CK, Fairley CK, Jordan L, Lewis JA, et al. Impact of text and email messaging on the sexual health of young people: a randomised controlled trial. J Epidemiol Community Health. 2012;66(1):69-74.
24. World Health Organization. WHO guideline: recommendations on digital interventions for health system strengthening Web Supplement 2: Summary of findings and GRADE tables. Geneva: WHO; 2019 2019. Report No.: WHO/RHR/19.7.
25. Gonsalves LLE, K.L.; Tamrat, T; Plourde, K.F.; Mangone, E.R.; Agarwal, S.; Say, L.; and Hindin, M.J. Adolescent/Youth Reproductive Mobile Access and Delivery Initiative for Love and Life Outcomes (ARMADILLO) Study: formative protocol for mHealth platform development and piloting. Reproductive Health. 2015;12(67).
26. Gonsalves LH, MJ; Bayer, A; Carcamo, CP; Gichangi, P; Habib, N; Mwaisaka, J; Say, L. Protocol of an open, three-arm, individually randomized trial assessing the effect of delivering sexual and reproductive health information to young people (aged 13–24) in Kenya and Peru via mobile phones: adolescent/youth reproductive mobile access and delivery initiative for love and life outcomes (ARMADILLO) study stage 2. Reproductive Health. 2018;15(126).
27. World Health Organization. Classification of digital health interventions v1.0. Geneva, Switzerland: World Health Organization; 2018.
28. Mwaisaka JG, Lianne; Say, Lale; Gichangi, Peter. “What’s that on your phone?” The aftermath of parents finding sexual and reproductive health messages on their children’s phone in coastal Kenya. Journal of Health and Social Sciences. 2018;3(2):147-56.
29. Chan M-pS, Jones CR, Hall Jamieson K, Albarracín D. Debunking: A Meta-Analysis of the Psychological Efficacy of Messages Countering Misinformation. Psychological Science. 2017;28(11):1531-46.
30. Lewandowsky S, Ecker UKH, Seifert CM, Schwarz N, Cook J. Misinformation and Its Correction: Continued Influence and Successful Debiasing. Psychological Science in the Public Interest. 2012;13(3):106-31.
31. Pluviano S, Watt C, Della Sala S. Misinformation lingers in memory: Failure of three pro-vaccination strategies. PloS one. 2017;12(7):e0181640.
32. Seifert CM. The continued influence of misinformation in memory: What makes a correction effective? Psychology of Learning and Motivation. 41: Academic Press; 2002. p. 265-92.
33. Free C, McCarthy O, French RS, Wellings K, Michie S, Roberts I, et al. Can text messages increase safer sex behaviours in young people? Intervention development and pilot randomised controlled trial. Health technology assessment (Winchester, England). 2016;20(57):1-82.

For peer review only - http://bmjopen.bmj.com/site/about/guidelines.xhtml

34. Perry RC, Kayekjian KC, Braun RA, Cantu M, Sheoran B, Chung PJ. Adolescents' perspectives on the use of a text messaging service for preventive sexual health promotion. The Journal of adolescent health : official publication of the Society for Adolescent Medicine. 2012;51(3):220-5.
35. da Silva-Filho AL, Lira J, Rocha ALL, Carneiro MM. Barriers and myths that limit the use of intrauterine contraception in nulliparous women: a survey of Brazilian gynaecologists. Postgraduate medical journal. 2017;93(1101):376-81.
36. Muthamia M, Owino K, Nyachae P, Kilonzo M, Kamau M, Otai J, et al. The Tupange Project in Kenya: A Multifaceted Approach to Increasing Use of Long-Acting Reversible Contraceptives. Glob Health Sci Pract. 2016;4 Suppl 2(Suppl 2):S44-S59.
37. L'Engle K, Plourde KF, Zan T. Evidence-based adaptation and scale-up of a mobile phone health information service. mHealth. 2017;3:11.
38. Lee S, Begley CE, Morgan R, Chan W, Kim S-Y. Addition of mHealth (mobile health) for family planning support in Kenya: disparities in access to mobile phones and associations with contraceptive knowledge and use. International Health. 2019;11(6):463-71.
39. McCarthy OL, Zghayyer H, Stavridis A, Adada S, Ahamed I, Leurent B, et al. A randomized controlled trial of an intervention delivered by mobile phone text message to increase the acceptability of effective contraception among young women in Palestine. Trials. 2019;20(1):228.

For peer review only

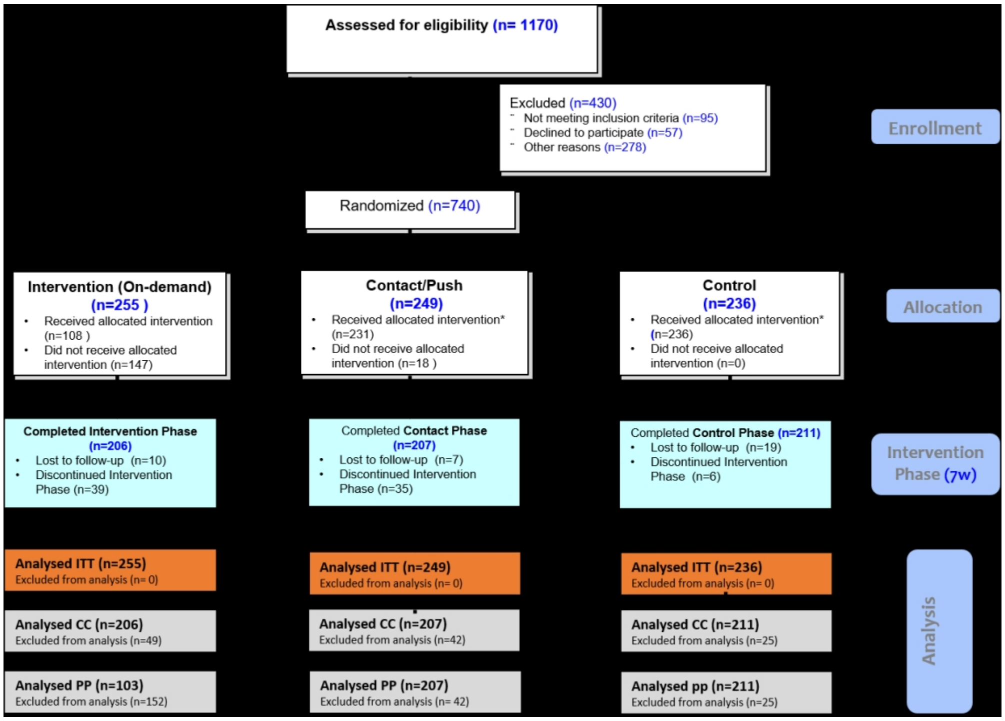

Figure 1. Consolidated Standards of Reporting Trials (CONSORT) diagram

233x166mm (150 x 150 DPI)

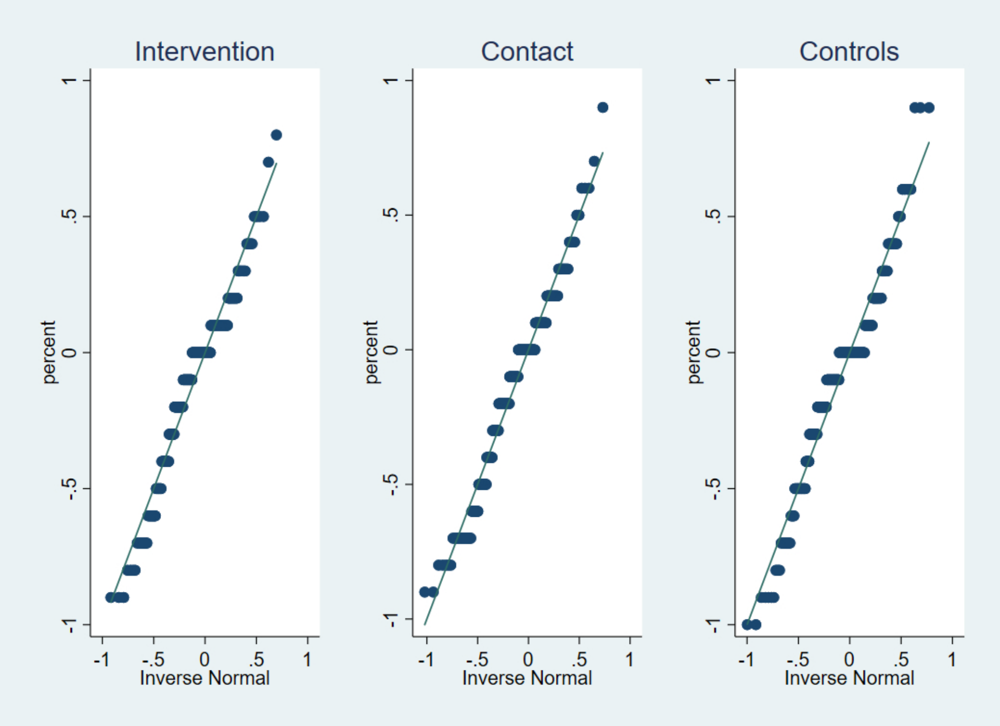

Figure 2: Checking the normality of the absolute changes in the myths believed

274x199mm (96 x 96 DPI)

Supplementary materials

Suppl. table 1: Assessing attrition bias across the study arms

Characteristic	Control (C)	Intervention (I)	Contact (P)	Assessing the baseline differences for drop-outs		
	N=25, n (%)	N=49, n (%)	N=42, n (%)	C vs I p-value	I vs P p-value	C vs P p-value
**Age of the participant**						
18 – 19	5 (20.0)	14 (28.57)	13 (30.95)	0.58	0.82	0.24
20 – 24	20 (80.0)	35 (71.43)	29 (69.05)			
**Sex**						
Male	12 (48.0)	24 (48.98)	21 (50.0)	1.00	1.00	1.00
Female	13 (52.0)	25 (51.02)	21 (50.0)			
**Highest level of school attended**						
Never gone to school	2 (8.0)	1 (2.04)	0 (0)	0.41	0.68	0.42
Primary school	8 (32.0)	21 (42.86)	16 (38.1)			
Secondary school	12 (48.0)	24 (48.98)	21 (50)			
Post-secondary education	3 (12.0)	3 (6.12)	5 (11.9)			
**Sub-location**						
Ngombeni	2 (8.0)	9 (18.37)	10 (23.81)	0.70	0.69	0.31
Kitivo	1 (4.0)	0 (0)	0 (0)			
Simkumbe	1 (4.0)	2 (4.08)	3 (7.14)			
Mkoyo	1 (4.0)	3 (6.12)	2 (4.76)			
Gombato	1 (4.0)	2 (4.08)	4 (9.52)			
Ukunda	19 (76.0)	33 (67.35)	23 (54.76)			
**Person currently living with**						
Living alone	1 (4.0)	4 (8.16)	3 (7.14)	0.66	1.00	1.00
Living with others	24 (96.0)	45 (91.84)	39 (92.86)			

For peer review only

**Current relationship status**						
Single	12 (48.0)	28 (57.14)	23 (54.76)	0.36	0.69	0.83
Friends with benefits / Dating / Cohabiting / Engaged	9 (36.0)	18 (36.73)	14 (33.33)			
Married	4 (16.0)	3 (6.12)	5 (11.9)			
**Number of children the participant have**						
None	16 (64.0)	41 (83.67)	37 (88.1)	0.14	0.87	**0.03****
1 child	7 (28.0)	7 (14.29)	5 (11.9)			
2+ children	2 (8.0)	1 (2.04)	0 (0)			
**First birth age**						
Never given birth	16 (64.0)	41 (83.67)	37 (88.1)	0.42	0.90	0.15
<=19 years (Adolescents)	5 (20.0)	5 (10.2)	3 (7.14)			
>=20 years (Young women)	2 (8.0)	3 (6.12)	2 (4.76)			
Missing	2 (8.0)	0 (0)	0 (0)			

Different rates of loss to follow up in the exposure groups, or losses of different types of participants, whether at similar or different frequencies may change the characteristics of the groups, irrespective of the exposure or intervention. This analysis was done to confirm if there were systematic differences among the people who were lost to follow up across the different study arms.

For peer review only - http://bmjopen.bmj.com/site/about/guidelines.xhtml

Suppl. table 2: Assessing attrition bias (drop-outs vs those who took the endline assessment)

Characteristic	Intervention (I)			Contact (P)			Control (C)		
	Drop-outs	Endline participants		Drop-outs	Endline participants		Drop-outs	Endline participants	
	N=49, n (%)	N=206, n (%)	Fisher's test p-value	N=42, n (%)	N=207, n (%)	Fisher's test p-value	N=25, n (%)	N=211, n (%)	Fisher's test p-value
**Age of the participant**									
18 – 19 years (Adolescents)	14 (28.57)	48 (23.3)	0.46	13 (30.95)	42 (20.3)	0.15	5 (20.0)	48 (22.8)	0.49
20 – 24 years (Young women)	35 (71.43)	158 (76.7)		29 (69.05)	165 (79.7)		20 (80.0)	163 (77.2)	
**Sex**									
Male	24 (48.98)	110 (53.4)	0.63	21 (50.0)	112 (54.1)	0.74	12 (48.0)	114 (54.0)	0.67
Female	25 (51.02)	96 (46.6)		21 (50.0)	95 (45.90		13 (52.0)	97 (46.0)	
**Highest level of school attended**									
Never gone to school	1 (2.04)	8 (3.9)	0.23	0 (0)	7 (3.4)	0.83	2 (8.0)	10 (4.7)	0.85
Primary school	21 (42.86)	71 (34.5)		16 (38.1)	81 (39.1)		8 (32.0)	72 (34.1)	
Secondary school	24 (48.98)	93 (45.2)		21 (50)	97 (46.9)		12 (48.0)	107 (50.7)	
Post-secondary education	3 (6.12)	34 (16.5)		5 (11.9)	22 (10.6)		3 (12.0)	22 (10.4)	
**Sublocation**									
Ngombeni	9 (18.37)	25 (12.1)	0.26	10 (23.81)	28 (13.5)	0.1	2 (8.0)	41 (19.4)	0.55
Kitivo	0 (0)	9 (4.4)		0 (0)	9 (4.4)		1 (4.0)	8 (3.8)	
Simkumbe	2 (4.08)	18 (8.7)		3 (7.14)	14 (6.8)		1 (4.0)	20 (9.5)	
Mkoyo	3 (6.12)	5 (2.4)		2 (4.76)	6 (2.9)		1 (4.0)	7 (3.3)	
Gombato	2 (4.08)	15 (7.3)		4 (9.52)	7 (3.4)		1 (4.0)	8 (3.8)	
Ukunda	33 (67.35)	134 (65.1)		23 (54.76)	143 (69.1)		19 (76.0)	127 (60.2)	
**Person currently living with**									
Living alone	4 (8.16)	20 (9.7)	1	3 (7.14)	18 (8.7)	1	1 (4.0)	20 (9.5)	0.71
Living with others	45 (91.84)	186 (90.3)		39 (92.86)	189 (91.3)		24 (96.0)	191 (90.5)	
Missing									
**Current relationship status**									
Single	28 (57.14)	100 (48.5)	0.58	23 (54.76)	98 (47.3)	0.41	12 (48.0)	106 (50.2)	0.68

For peer review only - http://bmjopen.bmj.com/site/about/guidelines.xhtml

Friends with benefits / Dating / Cohabiting / Engaged	18 (36.73)	15 (7.3)		14 (33.33)	19 (9.2)		9 (36.0)	22 (10.4)	
Married	3 (6.12)	91 (44.20		5 (11.9)	90 (43.5)		4 (16.0)	83 (39.3)	
**Number of children the participant have**									
None	41 (83.7)	183 (88.8)	0.4	37 (88.1)	174 (84.1)	0.46	16 (64.0)	175 (82.9)	0.06
1 child	7 (14.29)	17 (8.3)		5 (11.9)	23 (11.1)		7 (28.0)	28 (13.3)	
2+ children	1 (2.04)	6 (2.9)		0 (0)	10 (4.8)		2 (8.0)	8 (3.8)	
**First birth age**									
Never given birth	41 (83.67)	183 (88.8)	0.5	37 (88.1)	174 (84.1)	0.94	16 (64.0)	175 (82.9)	0.08
<=19 years (Adolescents)	5 (10.2)	13 (6.3)		3 (7.14)	19 (9.2)		5 (20.0)	18 (8.5)	
>=20 years (Young women)	3 (6.12)	10 (4.9)		2 (4.76)	14 (6.8)		2 (8.0)	16 (7.6)	
Missing	0 (0)	0		0 (0)	0		2 (8.0)	2 (1.0)	

Systematic difference between people who leave the study and those who continue can introduce bias in the study results, this is attrition bias. Suppl. table 2 shows the assessment of attrition bias which was achieved using fishers chi-square tests for categorical variables. This assessment was done to test whether participants lost to follow up differed from those who responded as a function of study group.

For peer review only

For peer review only - http://bmjopen.bmj.com/site/about/guidelines.xhtml

Suppl. table 3: Per Protocol analysis

Myth	Intervention (I) (n=103) Baseline	Endline	Diff.	Contact (P) (n=207) Baseline	Endline	Diff.	Control (C) (n=211) Baseline	Endline	Diff.
**Population based analysis**									
Hormonal contraceptives are fattening	72 (69.9%)	71 (68.9%)	-1.00%	142 (68.6%)	127 (61.4%)	-7.20%	139 (65.9%)	117 (55.5%)	-10.40%
Contraceptives can harm a woman's womb	62 (60.2%)	49 (47.6%)	-12.60%	144 (69.6%)	112 (54.1%)	-15.50%	135 (64%)	114 (54%)	-10%
People who use contraceptives end up with health problems	60 (58.3%)	52 (50.5%)	-7.80%	135 (65.2%)	100 (48.3%)	-16.90%	133 (63%)	103 (48.8%)	-14.20%
Contraceptives can cause cancer	59 (57.3%)	47 (45.6%)	-11.70%	123 (59.4%)	86 (41.6%)	-17.80%	117 (55.5%)	88 (41.7%)	-13.80%
Use of a contraceptive injection can make a woman permanently infertile	47 (45.6%)	35 (34%)	-11.60%	123 (59.4%)	77 (37.2%)	-22.20%	108 (51.2%)	82 (38.9%)	-12.30%
Contraceptives reduce women's sexual urges	36 (35%)	28 (27.2%)	-7.80%	107 (51.7%)	76 (36.7%)	-15%	101 (47.9%)	65 (30.8%)	-17.10%
Contraceptives can give you deformed babies	44 (42.7%)	26 (25.2%)	-17.50%	106 (51.2%)	71 (34.3%)	-16.90%	98 (46.5%)	69 (32.7%)	-13.80%
After a woman uses contraceptive methods, it is difficult to get pregnant	47 (45.6%)	32 (31.1%)	-14.50%	102 (49.3%)	80 (38.7%)	-10.60%	92 (43.6%)	81 (38.4%)	-5.20%
Birth control should be a female concern	20 (19.4%)	16 (15.5%)	-3.90%	59 (28.5%)	35 (16.9%)	-11.60%	59 (28%)	41 (19.4%)	-8.60%
Women who use family planning / birth-spacing may become promiscuous	52 (50.5%)	42 (40.8%)	-9.70%	111 (53.6%)	89 (43%)	-10.60%	109 (51.7%)	92 (43.6%)	-8.10%
**Subject specific analysis**									
Average # myths believed, per participant (Std. Error)	***5.17***	***4.04***		***5.01***	***3.90***		***5.57***	***4.12***	
Average absolute change in myths believed	***-1.13***			***-0.98***			***-1.44***		
Conf. interval of the diff.	***[-1.59, -0.67]***			***[-1.57, -0.39]***			***[-1.91, -0.98]***		

For peer review only - http://bmjopen.bmj.com/site/about/guidelines.xhtml

Percentage absolute change in myths believed	**-21.9%**	**-20.2%**	**-25.9%**
Test of diff. in the mean of the absolute change in myths believed	Control (n=211)	Intervention (n=103)	Contact (n=207)
ANOVA test F statistics	***0.81***		
P-value	***0.4458***		

For peer review only

For peer review only - http://bmjopen.bmj.com/site/about/guidelines.xhtml

Suppl. Figure 1. The interventions each arm received

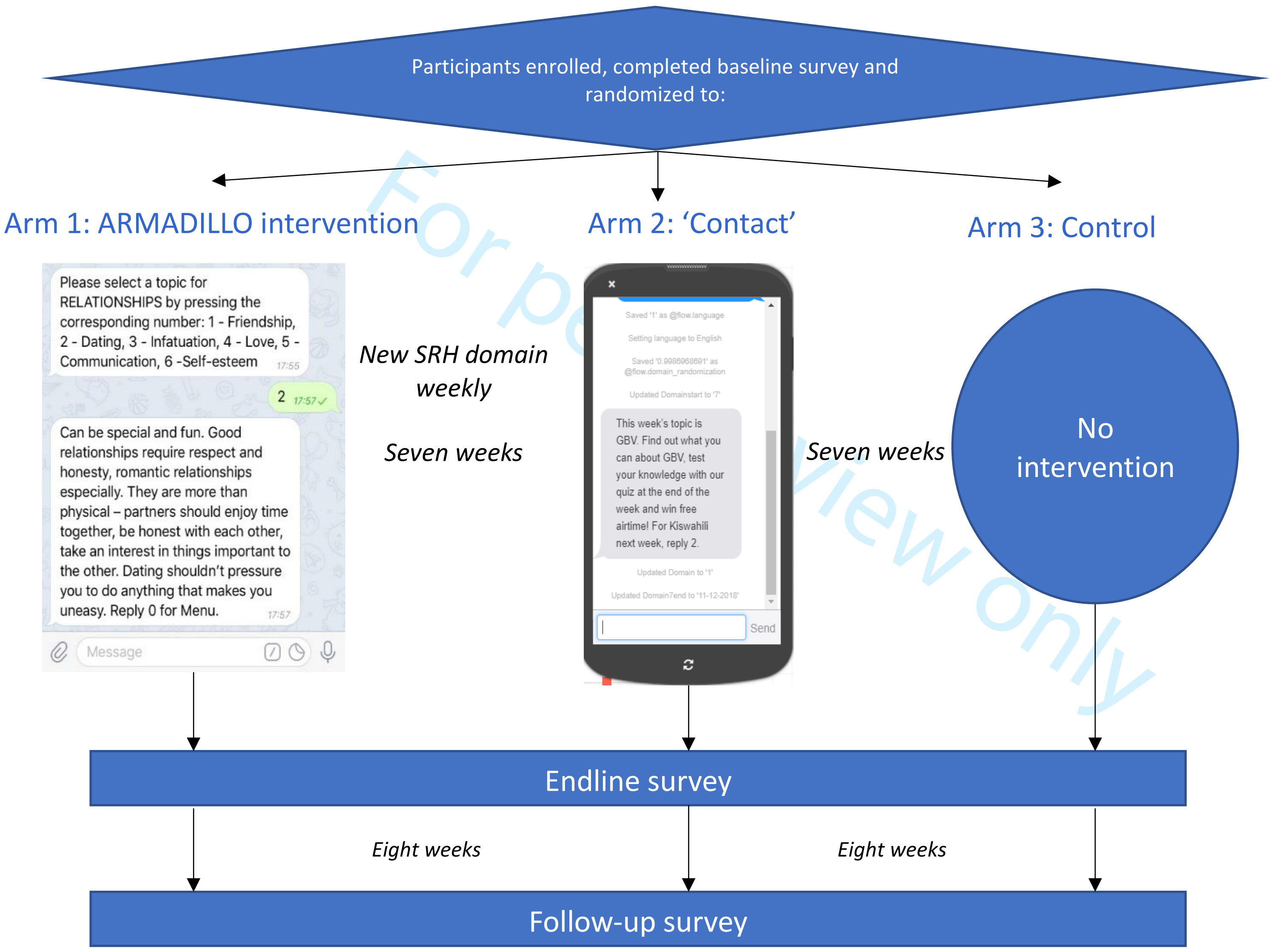

For peer review only - http://bmjopen.bmj.com/site/about/guidelines.xhtml

Suppl. Figure 2. The message domains which the intervention arms participants could request for more information and contacts arms participants studied on their own

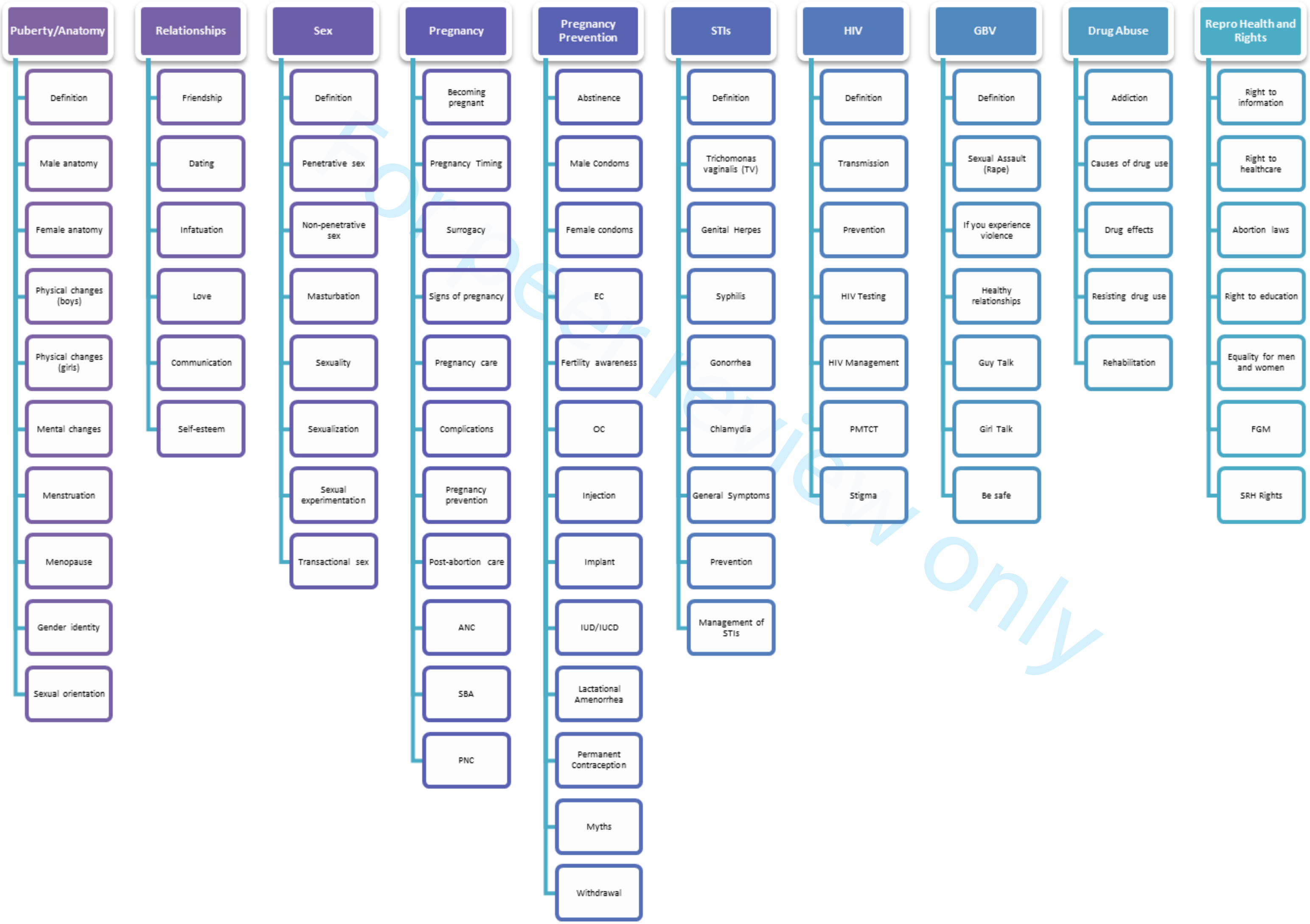

For peer review only - http://bmjopen.bmj.com/site/about/guidelines.xhtml

Adolescent/Youth Reproductive Mobile Access and Delivery Initiative for Love and Life Outcomes (ARMADILLO) protocol for research trial evaluating youth learning and information retention following delivery of SRH information via mobile phones.

human reproduction programme hrp research for impact UNDP·UNFPA·UNICEF·WHO·THE WORLD BANK

FOR WHO USE ONLY
Date received by WHO:
Thematic area:
☐ Single site proposal
☒ "Core" proposal (for multicentre study)
☐ Centre-specific proposal under multicentre study
Connect ID No:

Form II

HRP Research Project
General information

1. **Project title** Adolescent/Youth Reproductive Mobile Access and Delivery Initiative for Love and Life Outcomes (ARMADILLO) protocol for research trial evaluating youth learning and information retention following delivery of SRH information via mobile phones. **A-ID:**

If a continuation of an earlier project, provide name and ID of linked project: A65892 *core*, A65893, A65901

2. Principal Investigator - Kenya

Last name: Gichangi First name: Peter Title: Professor

Nationality: Kenya

Position: Country Director and CEO

Name of institution: International Centre for Reproductive Health – Kenya (ICRH-K) Web site: http://icrhk.org/

Head of institution: *same as above* Title:

Postal address: P.O.Box 91109 - 80103, Mombasa – Kenya

Telephone (office): +254 20 2179519/21 Mobile: +254 (0) 722 521946

E-mail: peter@icrhk.org; gichangip@yahoo.com

Are you employed > 50% at the institution named above : **YES** NO

Are you a previous or current HRP trainee or RCS grant recipient : YES **NO**

2. Principal Investigator - Peru:

Last name: Bayer First name: Angela Title: Dr

Position: Assistant Professor

Name of institution: Universidad Peruana Cayetano Heredia
web site: http://www.cayetano.edu.pe/cayetano/es/

Postal address: Av. Honorio Delgado 430, Urbanización Ingeniería, San Martin de Porres, Lima, Perú

For peer review only - http://bmjopen.bmj.com/site/about/guidelines.xhtml

Adolescent/Youth Reproductive Mobile Access and Delivery Initiative for Love and Life Outcomes (ARMADILLO) protocol for research trial evaluating youth learning and information retention following delivery of SRH information via mobile phones.

Telephone (office): Mobile: +51 (0)1-446-1845
E-mail: angelabayerx@gmail.com

Are you employed > 50% at the institution named above : **YES** NO

Are you a previous or current HRP trainee or RCS grant recipient : YES **NO**

3. Institution responsible for the research project :

Same as PIs Same as co-I Other:

4 Name of WHO RHR technical officer involved: Lianne Gonsalves

5 Key words

- Adolescents; digital health; youth; reproductive health; contraception

Research Plan

Purpose, aim and objectives:

There are 1.8 billion people between 10-24 years of age, 90% of whom live in low- and middle-income countries(1). Each year, 16 million girls between 15-19 years of age and two million girls under 15 years of age give birth. It is estimated that a lack of access to contraception leads to 7.4 million unintended pregnancies among adolescents, aged 10 to 19 (2, 3). Unintended pregnancies resulted in an estimated 3.2 million unsafe abortions worldwide in 2008, and complications related to pregnancy and childbirth are the leading cause of death for women ages 15-19 (3). There is a high unmet need for sexual and reproductive health (SRH) information and services, for both married and unmarried youth worldwide. However, financial, cultural, social, and legal considerations often impede youth access to the sexual and reproductive health resources that may be available in their communities(4).

While there are efforts in place to make facility-based services and health providers 'youth-friendly,' there is a need to strengthen the enabling environment for youth to actually seek care (5). This includes using channels outside of the facility to reach youth with high-quality SRH content and details about the SRH services that are available to them.

Many strategies have relied on creative uses of mass media to engage and educate youth, and the proliferation of mobile technology in recent years and its popularity with young people offers a particularly exciting modality with which to reach this age group. Of the nearly 6 billion mobile phone subscribers worldwide, approximately one-third are under the age of 30 (6). Given the proliferation of mobile phones, they offer the potential of discretely delivering tailored sexual and reproductive health content without stigma or judgment.

The World Health Organization's Department of Reproductive Health and Research partnered with research partners in Peru and Kenya to initiate the Adolescent/Youth Reproductive Mobile Access

For peer review only - http://bmjopen.bmj.com/site/about/guidelines.xhtml

Adolescent/Youth Reproductive Mobile Access and Delivery Initiative for Love and Life Outcomes (ARMADILLO) protocol for research trial evaluating youth learning and information retention following delivery of SRH information via mobile phones.

and Delivery Initiative for Love and Life Outcomes (ARMADILLO) Study. The goal of ARMADILLO is to develop and evaluate an on-demand system for youth to access and receive SRH information through short message service (SMS, also known as 'text message'). ARMADILLO is envisioned as a three- stage study (Figure 1): Stage1--developed and tested the message content that will form the ARMADILLO system (A65892 core); Stage 2 (this protocol) —assess the effect of ARMADILLO on knowledge for action, attitudes and self-efficacy using quantitative and qualitative methods; and Stage 3-- a coverage study.

Stage 1 Summary

In Stage 1, Kenya and Peru sites both underwent processes to develop and test the messages that would form the ARMADILLO architecture. In Kenya, data collection took place in Mtwapa a peri-urban town in Kilifi County, Kenya. Mtwapa was home to 48,625 people during the 2009 census(7). Mtwapa was selected based on a number of 'Drop-In Centres' implemented by the International Centre for Reproductive Health – Kenya (ICRHK, the Kenya site research implementer) - which serve as a combination of health and social centres for adolescents and youth – as well as the strong community ties and partnerships with youth organizations that ICRHK had in this area. In Peru, following strong recommendations from implementing partner Universidad Peruana Cayetano Heredia (UPCH), data collection for the formative phase actually took place in three sites in order to ensure buy-in from across Peru's distinct geopolitical regions: the coast (Lima), the jungle (Yurimaguas) and the highlands (Ayacucho).

Prior to the start of formative research activities, both researching partners developed draft messages, in close collaboration with local stakeholders. The exact make-up of stakeholder groups varied slightly across sites, however, both sites developed draft messages with a combination of regional/national level stakeholders in adolescent/youth sexual and reproductive health, as well as adolescent/youth involvement themselves.

In Kenya, the resulting messages were categorized by theme (e.g. pregnancy/drug abuse/relationships), while in Peru messages were grouped under overarching questions (e.g. 'Who am I?' 'Who takes care of me?'). The inclusion of youth as equal participants in the identifying of messages topics and content was critical, as youth often identified topics of interest that adult stakeholders had not considered or might not have thought important (for example, youth in Kenya were keen to explore the differences between 'infatuation' and 'love' as concepts).

In both sites, formative research activities began when the initially-developed messages were vetted for comprehension, relatability, and completeness through a series of Focus Group Discussions with young people aged 15-24 in Kenya and 13-24 in Peru. FGD participants were grouped to be homogenous in sex and age range (15-17 and 18-24 in Kenya, 13-17 and 18-24 in Peru). In all FGDs, youth were asked to provide insight into the following: young people's use of mobile phones; feedback on a series of the proposed ARMADILLO messages; perceived reactions if siblings, parents and/or significant others were to discover ARMADILLO messages on the phones of a user. In Kenya, following the completion of FGDs, a small survey of possible SRH outcomes was piloted among 250 young people as a way to plan for Stage 2.

Additionally in both sites, a series of FGDs were carried out with parents/caregivers of young people. Parents and caregivers were invited to share their thoughts on where young people learned about SRH issues; how parents handled discussing SRH issues with their children; thoughts on youth access to contraception; and perceived parent reactions if they were to discover their

For peer review only - http://bmjopen.bmj.com/site/about/guidelines.xhtml

child accessing ARMADILLO messages. In Peru, no survey of possible SRH outcomes was completed, as by the time data collection took place, outcomes for Stage 2 were already decided.

Brief summaries of the formative stage in each site are as follows:

Kenya:

A total of 12 FGDs with 95 young people aged 15-24 were conducted, as were an additional four FGDs with a total of 33 parents/caregivers of young people. Findings from these focus groups established that a majority of the youths aged 18-24 had their own personal mobile phones. Meanwhile, adolescents aged 15-17 had phones but mostly used when not in school and shared phones with their siblings and other relatives they lived with. Some even shared with their neighbors and friends especially when doing MPESA (mobile money transfer) transactions. 18-24 year olds reported not to be sharing their phones with anyone save for their confidants whom they trusted.

On getting information about ASRH, it was noted a large majority of young people are afraid to visit health facilities; some said they do not have anyone to turn to for such talks for fear of being labeled ill-mannered boy or girl. Those afraid to visit the health centers opt to look for information online. Youth identified online information as one of their only 'reliable' sources of information. SRH information from friends was reported not to be reliable as they equally lacked adequate knowledge to address their concerns. Parents were considered reliable by the youth in giving SRH information; however SRH discussions rarely happened as they were considered culturally inappropriate. Schools were also reliable in giving the SRH knowledge, though the completeness of information was often lacking, leaving young people with many unexplained concerns.

Therefore, when presented with the ARMADILLO platform, a majority of youth agreed they would use it as the information was deemed credible and had undergone due process. It's was also appreciated for being private and confidential, as young people otherwise lacked such places to obtain information about their SRH. It was also mentioned that young people would be happy with ARMADILLO providing information to SRH concerns in real time.

With regards to concerns about privacy, parents coming across ARMADILLO messages was a concern expressed by some young participants, though a majority didn't have any problem with their peers accessing their phone's contents. The general perception was that parents would react differently depending on the age of the one accessing the content: for example, it was reported that those below the age of 18 will be scolded by their parents if seen to be accessing SRH messages while those above 18 years will be considered matured and although their parents won't be pleased, nothing would be done to them.

By contrast, FGDs with parents and caregivers found them to be in full support of their youth accessing SRH information through text messaging. Parents and caregivers showed strong approval of the service and commended the messages in that they will provide them with a platform to continue with the SRH conversation which at times is difficult to start adding that they would give advice where necessary as well as encourage them to share the information with their younger siblings.

Stage one of the ARMADILLO study established that mobile phones are currently part of young people's lives and reinforced the fact that sexual and reproductive health messages delivered via

For peer review only - http://bmjopen.bmj.com/site/about/guidelines.xhtml

For peer review only

text messages from a trusted source will most likely be acceptable to young people owing to its confidential nature.

Peru

Stage 1 was carried out in three sites and two steps. The three sites were Lima, the capital city, Yurimaguas in the jungle region, and Ayacucho in the highlands region. For the first step, we worked with local expert committees to develop the potential domains and sub-domains for the ARMADILLO system. These committees included 32 13-17 year olds (22 females and 10 males) and 36 18-24 year olds (3 females and 13 males) and 23 adults from the health and education sectors. Together, these groups developed 7 domains and 25 sub-domains, as well as 2 cross-cutting themes for the ARMADILLO system. Based on these identified domains and sub-domains, the UPCH team developed SMS text messages for each sub-domain. As in Kenya, FGDs were used to validate the SMS text messages with youth. In this step, 104 13-17 year olds (54 females and 50 males) participated. They provided their individual evaluation about how much they liked or didn't like each text message and their detailed group feedback about each text message (overall content, tone, language).

Additionally, the team also asked adolescent participants about phone sharing and about their perspectives on people finding the ARMADILLO SMS text messages on their phone. About 1 in 4 participants reported sharing their phone with someone else, including their parents, siblings, partners and friends. When asked what they would think about these different groups of people finding their phone and the ARMADILLO messages, unlike youth in Kenya, Peruvian young people stated that it would be fine as long as it is clear that the messages are "public service messages." It should be clear that they are sent out to many people at once and from a respectable sources. They mentioned that these types of text messages are common in Peru, including for health issues. For example, there have been mass text messages for information of HPV vaccination for girls.

Stage 1 protocols A65893 and A65901 also contain national-level background about both sites, which can be referenced if one wishes to understand how Peru and Kenya were selected as study sites. This Stage 2 protocol builds from the Stage 1 foundation.

Figure 1

For peer review only

For peer review only - http://bmjopen.bmj.com/site/about/guidelines.xhtml

Adolescent/Youth Reproductive Mobile Access and Delivery Initiative for Love and Life Outcomes (ARMADILLO) protocol for research trial evaluating youth learning and information retention following delivery of SRH information via mobile phones.

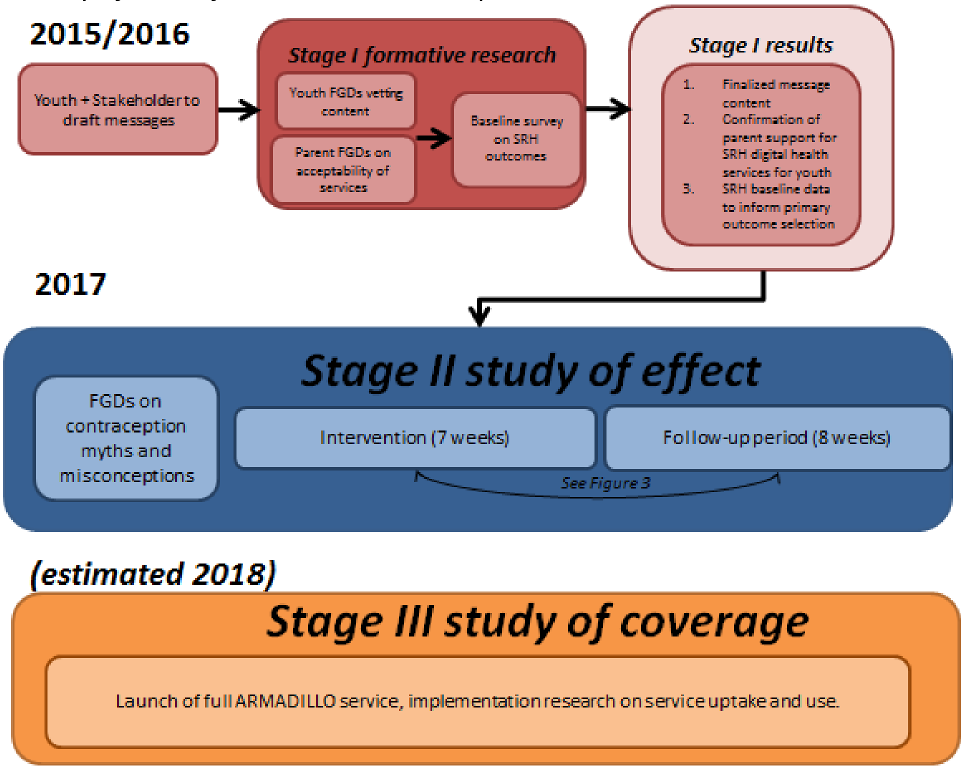

The ARMADILLO Intervention

In making decisions about their sexual and reproductive health, young people fall on a spectrum of contraceptive use patterns (8), which range from never having used contraception to being a current user of most effective contraceptive methods (long-acting reversible contraceptive methods).

Figure 2

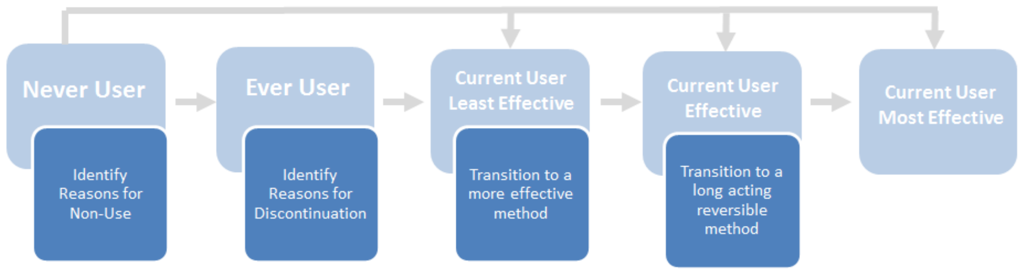

Model of Contraceptive Use Patterns

This spectrum (Figure 2, above), is fluid, with people likely moving through multiple categories throughout their youth and reproductive years in general. At each point of transition, different strategies may be required to support individuals, should they want to avoid a pregnancy. Access to clear and accurate information, in response to relevant perceptions and/or misconceptions at each transition, is a foundation of all strategies. ARMADILLO serves as a resource of validated SRH information for young people, using a mode of delivery which assures their privacy and comfort.

ARMADILLO consists of an automated, interactive, and on-demand SMS platform that will provide essential facts and address common misconceptions about a full range of SRH issues pertinent to

For peer review only - http://bmjopen.bmj.com/site/about/guidelines.xhtml

Adolescent/Youth Reproductive Mobile Access and Delivery Initiative for Love and Life Outcomes (ARMADILLO) protocol for research trial evaluating youth learning and information retention following delivery of SRH information via mobile phones.

youth, including puberty, relationships, sex, pregnancy, HIV and STIs, and contraception. The ARMADILLO system will be available to users at no charge.

Study Objectives:

This research protocol will use an individually randomized design to assess differential learning related to sexual reproductive health and rights compared to those who are asked to learn on their own.

Primary Objective: To determine whether youth given access to ARMADILLO's targeted SRH information through their mobile phones are more knowledgeable about contraception and better able to dispel contraception myths and misconceptions than those without access to ARMADILLO.

Secondary Objective: To determine whether youth given access to ARMADILLO's SRH information through their mobile phones retain information on SRHR longer and more accurately than those without access to ARMADILLO.

Survey of the field:

Programs using mobile phones have been used in many areas of health care and health promotion globally(9-13). Recent reviews of studies using mobile phones to promote behaviour change have demonstrated mixed to positive effects of such programs (14-17). Studies in the United States, Australia, and South Africa, for example, confirm that text messaging programs for reproductive health can lead to better sexual health and increased use of health services (18, 19). Text messaging programs are a commonly used channel, as texting is the most frequent form of mobile phone communication (20, 21), and is available on every mobile phone regardless of phone model or mobile network provider, with minimal costs per message.

There are numerous projects being implemented globally that leverage mobile technologies for improved youth SRH. Protocol A65893 for the ARMADILLO Study's formative phase describes in detail some of these projects. As the protocol also highlights, despite enthusiasm for the use of mobile phone technology to reach young people on SRH issues, there is very minimal data to provide insight on the impact of this strategy for increasing knowledge, affecting norms, promoting self-efficacy, and/or improving behaviour. The global health community has issued repeated calls for evidence, particularly around the impact of mobile phone approaches on achieving improved health of populations (22, 23).

The ARMADILLO platform's structure of on-demand access to SRH information delivered via SMS is but one strategy that capitalizes on young people's widespread use of mobile and electronic devices to improve information on and access to SRH services. However, ARMADILLO opts to work exclusively with SMS in order to maximize its scalable potential - SMS is a channel for communication available on all mobile phones.

Youth as a group appear to be especially enthusiastic adopters of mobile technology as demonstrated by their comfort using and communicating over various channels on feature and smart phones. Additionally the nature of mobile phone technology itself ensures a level of discretion and privacy that would otherwise not exist when reaching out to this age group on sensitive issues, such as their sexual and reproductive health. Mobile devices can also

For peer review only - http://bmjopen.bmj.com/site/about/guidelines.xhtml

theoretically transcend gender, marital status, and other demographic information which may otherwise serve as obstacles to accessing SRH information.

The ARMADILLO architecture was developed in each country setting using national and global guidelines around youth sexual and reproductive health, with message wording and content vetted by youth themselves during Stage 1 (Annex 2). This platform is meant to catalyse additional conversations and information- and service-seeking about SRH by providing essential, guidelines-based information which remains available for access at the moment it is needed (and which can be saved for future access).

Project description:

Study Design

The study uses an individually-randomized, open three-arm comparative design to assess the objectives above. After obtaining written informed consent, individuals randomized to the 'intervention' arm of the ARMADILLO trial will receive access to ARMADILLO content over the course of the intervention and will use their mobile phones to access this content on demand. Those randomized to the 'control' arm will receive no intervention. Arm 3 will differ by site: in Kenya, those randomized to this 'contact' arm will be alerted to various SRH domains (e.g. health topics, including Relationships, Pregnancy, STIs, etc) and be encouraged to learn on their own; in Peru, those randomized to the 'push' arm will receive key messages daily with the option to learn more if they wish.

We have elected to include three arms, rather than the traditional 'intervention' and 'control' arms in an attempt to address lingering questions around the efficacy of informational digital health interventions in different settings. Across all arms, selected SRH outcomes will be assessed via a survey at baseline (prior to the intervention start), at intervention end, and at eight weeks (two months) following the end of the intervention. All outcomes of interest are linked to domains and content from the ARMADILLO system. Control group participants will receive no messaging, only assessments at baseline, and times corresponding to the intervention end and follow-up for the other two groups.

Data collection methods

Prior to the start of the study, as part of a finalization of study tools, a number of Focus Group Discussions will be conducted in order to confirm young people's key myths and misconceptions around contraceptive use, common reasons for not using contraception, as well as build an understanding of where young people access contraception and why. Additionally, in order to streamline the generic study questionnaire (which will assess participant learnings at baseline, end-intervention, and follow-up) to each site as well as ensure comprehension, necessity, and relevance of items for the different age groups being assessed, both Kenya and Peru will conduct a small pilot of the tool among 25-30 youth.

The selected methodology for the effect assessment involves a combination of close-ended surveys and in-depth interviews conducted in person, as well as short, spot assessments via mobile phone. All in-person study activities will take place in a private room at a location and time that is convenient. These rooms allow for both visual and auditory privacy. A project staff member will explain the study activity and answer any questions prior to and/or following completion of the given study activity.

For peer review only - http://bmjopen.bmj.com/site/about/guidelines.xhtml

Adolescent/Youth Reproductive Mobile Access and Delivery Initiative for Love and Life Outcomes (ARMADILLO) protocol for research trial evaluating youth learning and information retention following delivery of SRH information via mobile phones.

In-person baseline assessment

All participants will complete a baseline, close-ended survey with questions linked to ARMADILLO content. The research team staff will then explain the participant's expected interaction with the three arms to which they might be randomized and answer any questions. Participants will be told that, should they be randomized to intervention or Arm 3, they can be expect their first message from the system the following day. After the participant leaves, the participant will be randomly assigned to one of the three arms.

Study arms implementation

Generally, messages pushed to phones incur no cost for the recipient. However, the study design requires participants in two of the three arms to send messages as well as receive them – either in response to quiz questions, or in requesting content from ARMADILLO. Message sending does incur charges for the sender (in this case, ARMADILLO study participants). Therefore, both intervention arm and 'arm 3' (the format of which varies per site) will be zero-rated, with any SMS charges reversed billed to the study. Participants, during the enrolment process, and periodically during the intervention, will be reminded that any interaction with the study is free. Participants will also be periodically reminded that they can opt out at any time by sending a 'STOP' command to a short code.

Despite findings from formative research (see summary of Stage 1 findings above), there is a lingering ethics concern that a person may choose to exclude themselves from a study because their access to messages has somehow compromised their comfort or security. As such, should a member of either SMS-receiving arms indicate via shortcode that they wish to be unenrolled (responding 'STOP' to any message received), they will be immediately and automatically unenrolled from the study. Additionally, in order to answer a need ethical/implementation question, they will be sent one follow up message stating:

'You are unsubscribed from ARMADILLO and will receive no further contact. If you wish, please tell us why you unsubscribed. Respond 1) don't like them 2) someone saw them 3) messages don't relate to me'

Intervention Arm

For those assigned to the intervention arm, rather than offering access to the full ARMADILLO architecture at once (the extensiveness of which would make immediate assessment of relevant outcomes difficult), they will instead be provided access to one new domain (SRH topic) every week (Day 1 of 7 of a given week) and assessed on domain-specific outcomes at the end (Day 7 of 7) of that week. Within this domain, they will have the ability to navigate freely across and between messages, accessing subdomains that interest them and ignoring those that don't. At the start of the next week (Day 1 of 7), access to the previous week's domain is closed – meaning that the user can no longer access the information in that domain, though any messages the user has already received remains in his/her phone unless deleted by the user. At the same time, the next domain is 'unlocked' and the user receives a message alerting them to this new access.

Progression through the intervention will be as follows:

- Start of Week 1: an SMS alerting intervention participants to a new domain is pushed to their phones (e.g. 'You've unlocked PREGNANCY. Want to learn more? Send 'ARMADILLO' to XXX – free and confidential! Stay tuned for a quiz at the end of the week to win free airtime!').

For peer review only - http://bmjopen.bmj.com/site/about/guidelines.xhtml

- Week 1, Days 1-7: participants can access all subdomain messages in either Swahili or English (Kenya) or Spanish (Peru) within this given domain, as many times as they would like, without charge.
- Week 1 (Day 7): a set of 3-5 close-ended SRH outcome-assessing questions are pushed via SMS to the participants' phones. Participants receive free airtime for any response (whether or not it is correct).
- Week 2, Day 1: access to Week 1 domain ends and intervention participants receive an SMS alerting them to the new domain they can now access.
- Weeks 2-7: The pattern described for Weeks 1 and 2 continues until participants have received one week of access to all domains. Access is not cumulative.

Control Arm

Some participants will be randomly allocated to the control arm. Control arm participants will receive no messages on their phone during the intervention period. Instead, they will only participate in baseline (described above), and end-intervention and follow-up assessments (described below).

Arm 3: 'Contact'(Kenya), 'Push' (Peru)

The third arm has been developed in close collaboration with each study site, and aims to address specific questions from countries and/or the digital health literature.

<u>Kenya</u>

In Kenya, the third arm will serve as a 'contact' arm, assessing whether changes in outcomes are attributable to the content of the intervention or rather the participant-contact nature of the intervention itself. As such, the inclusion of a 'contact' arm will match system-initiated contact with study participants without providing them access to ARMADILLO content. If the success of this digital health intervention is merely in participant contact (i.e. encouraging users to consider a relevant SRH topic) rather than the content of the ARMADILLO platform, we will find no differences in outcomes between intervention and contact arms and participants in both arms would likely have better outcomes than control participants.

Participants in Kenya's contact arm will not have access to ARMADILLO message content. They will, however, receive the same number of system-initiated contacts (pushed messages) that intervention arm receives: specifically, they will receive messages alerting them to a new SRH domain at the beginning of each week, and the domain-specific assessment at the end of the week. Contact participants will receive each domain as a 'topic of the week' and their weekly introduction SMS will encourage them to learn on their own that week; no additional information on that topic will be provided.

Progression through the contact arm will be as follows:

- Start of Week 1: an SMS alerting contact participants to a 'topic of the week' is pushed to their phone (e.g. 'This week's topic is PREGNANCY. Find out what you can about pregnancy, test your knowledge with our quiz at the end of the week and win free airtime!').
- Week 1, Day 7: There is no further interaction with the contact arm until the end of the week, when a set of 3-5 close-ended SRH outcome-assessing questions about that week's topic are pushed via SMS to the participants' phones. Participants receive free airtime for any response (whether or not it is correct).

For peer review only - http://bmjopen.bmj.com/site/about/guidelines.xhtml

Adolescent/Youth Reproductive Mobile Access and Delivery Initiative for Love and Life Outcomes (ARMADILLO) protocol for research trial evaluating youth learning and information retention following delivery of SRH information via mobile phones.

- Week 2, Day 1: access to Week 1 topic ends and contact participants receive an SMS alerting them to the new topic that they can now learn about.
- Weeks 2-7: The pattern described for Weeks 1 and 2 continues until participants have received one week of suggested topics.

Figure 3 shows the progression of all three arms in Kenya.

Figure 3

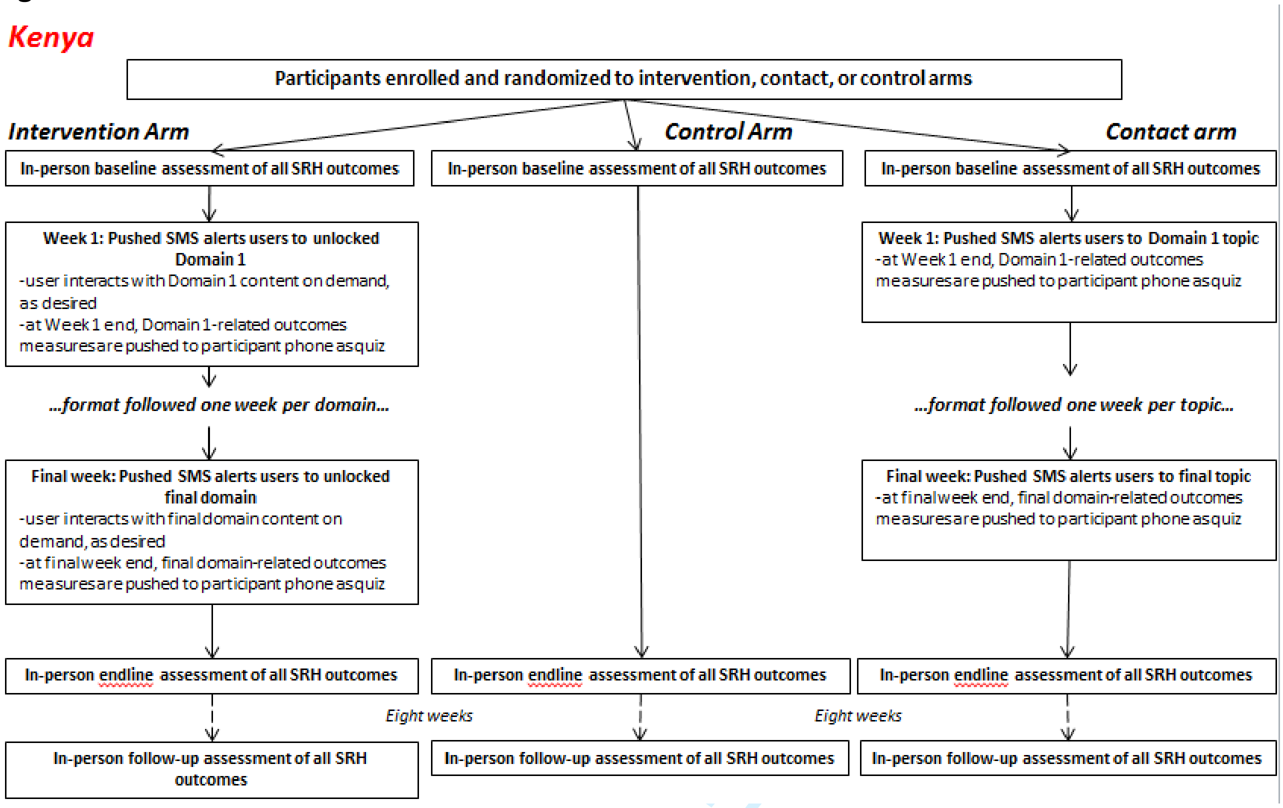

<u>Peru</u>

In Peru, push-message public health campaigns are the norm for a wide variety of sensitive (re: HPV vaccination) and non-sensitive (maternal health, nutrition, etc) health campaigns. Bi-directional interventions, such as ARMADILLO, do not exist; this will make the format of the ARMADILLO intervention arm a novelty for Peru. As such, Arm 3 for Peru will adopt the Peruvian 'push system' norm, answering a question important for adoption and scale up to national level in this context: is free, active engagement – while a novelty in this setting – better for engaging with young users than passive receipt and reading of messages (the norm in Peru)?

Progression through the push arm will emulate existing public health campaigns in Peru and will be as follows:

- Start of Week 1: an SMS alerting participants to a 'topic of the week' is pushed to their phone (e.g. 'This week's topic is PREGNANCY. Not interested? Respond XX to opt out this week').
- Week 1, Day 2-5: 1-2 SMS messages are at the same time daily (evening, when participants have left school, where phones are not generally allowed) to participants' phones, covering various subdomains from the week's topic
- Week 1, Day 6: At the end of the 1-2 SMS messages which are pushed to phones, participants have an option to 'responde XX para leer mas' [respond to read more]. If they respond, they will immediately receive an additional maximum of 4 messages.

For peer review only - http://bmjopen.bmj.com/site/about/guidelines.xhtml

Adolescent/Youth Reproductive Mobile Access and Delivery Initiative for Love and Life Outcomes (ARMADILLO) protocol for research trial evaluating youth learning and information retention following delivery of SRH information via mobile phones.

- Week 1, Day 7: Close-ended SRH outcome-assessing questions about that week's topic are pushed via SMS to the participants' phones. Participants receive free airtime for any response (whether or not it is correct).
- Week 2, Day 1: access to Week 1 topic ends and push participants receive an SMS alerting them to the new topic that they will now learn about.
- Weeks 2-7: The pattern described for Weeks 1 and 2 continues until participants have received one week of suggested topics.

Figure 4 shows the progression of all three arms in Peru.

Figure 4

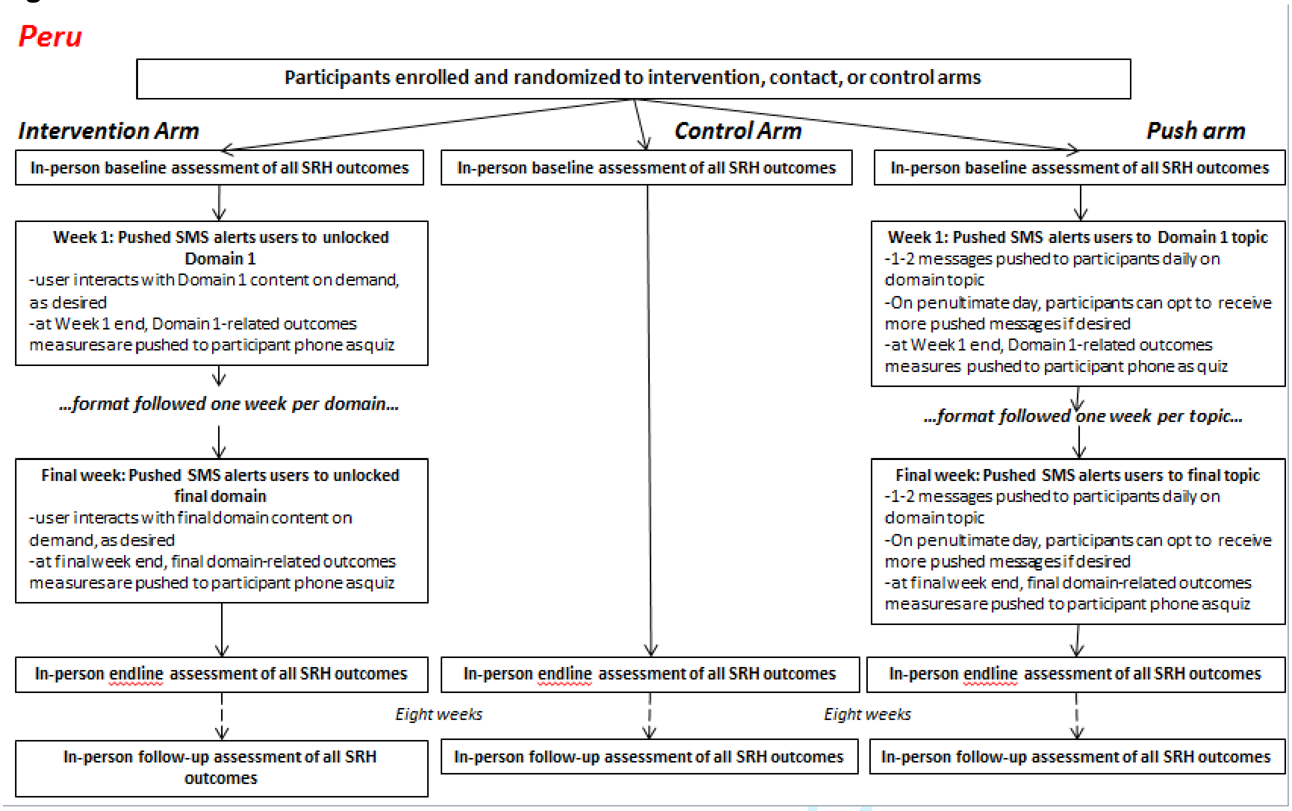

Arm 3 and intervention participants in both sites will progress through domains sequentially, based on numbers assigned to each domain (for example, a participant starting at Domain 1 would progress through Domains 2-7 in order in the following weeks). However, participants will be randomized as to which number domain they start with, in order to account for participant recall bias at the end-intervention assessment as a result of having seen one domain 7-8 weeks ago and another one week ago. For example, an intervention/Arm 3 participant may be randomly assigned to start at Domain 3, meaning they would cycle through Domains 4-7 in the following weeks before finishing with Domain 1 and 2. It should be noted that the free airtime intervention and Arm 3 participants receive – meant to subsidize perceived expense for participating in the system – will be credited to users by the study team and does not require negotiation with the mobile network operating companies (MNOs).

End-intervention assessment

Participants from all three arms will be asked to return for an in-person assessment of all outcomes at the end of the intervention. Participants will be able to fill out the end-line survey individually in a private space. Research team members will update participant contact

For peer review only - http://bmjopen.bmj.com/site/about/guidelines.xhtml

information if necessary and alert participants that they will be invited back for a follow-up assessment in two months

At this visit, a selected sub-sample of intervention participants will also be invited to participate in an in-depth interview. A trained member of the research team will consent these participants and conduct the interview. This interview will provide an opportunity for participants to provide feedback on their experience and opinions using the system. Of particular interest will be recommendations for system improvement; if and with whom they shared messages; perceived ease or difficulty in translating knowledge obtained through ARMADILLO into action (if action was desired or necessary); where they obtained contraception (if applicable); and any recommendations for improving the experience of obtaining contraception.

In Peru, this sub-sample of participants to be interviewed will be evenly split between intervention and Arm 3 (push arm) participants (10 participants interviewed from each arm). This will allow a comparison of user experiences between the push arm (the current standard in Peru) and intervention arm (a novelty), the data from which will be important an important complement to survey data in deciding on a format for scale-up in future planned phases.

<u>Follow-up assessment</u>

There will be no contact with participants from any study arm in the eight weeks between the end of the intervention and follow-up. The ARMADILLO system will be offline in this period as well. Two months following the end-line assessment, intervention, control, and Arm 3 participants will be brought back for an in-person, follow-up assessment of all SRH outcomes.

Starting at Week 7 after the completion of ARMADILLO, the research team will contact participants again, to schedule a time - at the participant's convenience – when the participant can return to the study site to complete one final, in-person assessment around the SRH outcomes. Raffles and airtime may also be used to encourage participants to return for the follow-up assessment.

Following the completion of the in-person assessment, returning participants in all arms will be provided with a short code to access the full ARMADILLO system. In the event that the system must undergo substantial revision prior to finalization, study participants will be told that they will be alerted via SMS when the final system is ready for access.

Participants will be able to fill out the baseline survey individually in a private space.

Study Setting

Kenya : The Kenyan site for the ARMADILLO stage 2 will be Kwale County. Kwale County is one of the six counties in the Coastal region of Kenya. The total population of Kwale County was projected to be 713,487 persons in 2012 comprising of 346,898 males and 366,589 females. This is a 9.8% increase from 649,931 in 2009 national census. The County population growth rate is 3.1% and the sex ratio is 95 males per 100 female. Those <15 years of age in 2012 constituted 47.23% of the entire population. 19.0% of the entire population in Kwale County constituted of young people aged 15-24 in the year 2012. The County's total fertility rate is 6 children per woman(24). 49.0% of births are delivered at a health facility with the County having a contraceptive prevalence of 38.2% below the Country's which is at 53.2%(25).

For peer review only - http://bmjopen.bmj.com/site/about/guidelines.xhtml

Adolescent/Youth Reproductive Mobile Access and Delivery Initiative for Love and Life Outcomes (ARMADILLO) protocol for research trial evaluating youth learning and information retention following delivery of SRH information via mobile phones.

Age at first marriage as reported in KDHS 2014 for women in Kwale is 19.1 years while median age at first sexual intercourse for women was reported at 16.6 years and men 18.5 years . The County also has a high percentage rate of teenage pregnancies with 24.2% of women aged 15-19 having begun childbearing which is more than the Country's average of 18%(26). In Kwale County 253,041 people have access to mobile phones of which 62,473 are in Kwale town, 72,865 are in Kinango and 117,703 are in Msambweni(27).

Prior to the start of the study, three geographically distant locations will be selected within Kwale County with the help of the Kenya National Bureau of Statistics (KNBS) as the Intervention, Contact and Control arms. The geographic distance will help reduce contamination.

Peru: The study will take place in Peru, located in Western South America and home to about 31 million people. The country's population is distributed across three geographic regions, the coast, the jungle and the highlands. This stage of ARMADILLO will take place in the capital city of Lima, on the coast of Peru and home to 9.8 million people or 31% of the population. About one-third of Peru's adolescents live in Lima, with the highest proportions of youth living in peripheral areas similar to our study site(28).

The specific ARMADILLO study site will be Pampas de San Juan de Miraflores. Located in the southern cone of Lima, "Pampas" is one of the seven zones in the district of San Juan de Miraflores, which is one of Lima's 43 districts. Pampas' 46 human settlements (pueblos jóvenes) are home to approximately 50,000 residents(29). All participants in ARMADILLO will be recruited from Pampas.

Participants

For the purposes of this trial, where increased interaction with the system is anticipated and use of the phone for outcome measurement and follow-up is necessary, phone ownership is a requirement for participation. The same selection criteria will apply also to youth recruited for participation in the FGDs prior to the start of the effect assessment.

Eligibility criteria for the general ARMADILLO study is as follows:

- Youth between the ages of 13-24 (age range narrowed as needed for each site);
- Literate
- Have their own mobile phone (meaning it is primarily in their possession, and they control when and with whom they share access) and report regular use
- Have a mobile phone with them at the time of recruitment
- Report current use of text messaging

Recruitment to the FGDs preceding the trial will be purposive. Recruitment to the trial itself will be rolling over a one month period (recruitment is described more fully in the following section). Participants will be consented at their homes. Depending on the comfort level of the participant, they will continue either in their homes or be invited to meet a central location to participate in the baseline survey and be enrolled in one of the three ARMADILLO study arms. Randomization into intervention, control or Arm 3 arms will take place following completion of the baseline survey, with their seven-week interaction with the appropriate system commencing the following day. A research team member who is NOT interacting with participants will oversee the randomization of incoming phone numbers to the three arms.

For peer review only - http://bmjopen.bmj.com/site/about/guidelines.xhtml

Adolescent/Youth Reproductive Mobile Access and Delivery Initiative for Love and Life Outcomes (ARMADILLO) protocol for research trial evaluating youth learning and information retention following delivery of SRH information via mobile phones.

In-depth interview (IDI) participants will be randomly sampled from within the pool of participants who, during their consent process, indicated they would be willing to participate in an IDI. Ten youth females and ten youth males from each country site will be selected by computer. At their end-line in-person assessment, members of the research team will invite the selected participants to participate in the IDI. Should any members indicate that they are no longer willing to participate, the computer will randomly select additional participants from the remaining pool.

In Kenya, participants will be between the ages of 18 and 24 years of age. In Peru, participants will be between 13-17 years old. The difference in age in each site is a reflection of formative phase findings and results dissemination (see summary of Stage 1 findings at the beginning of this proposal). In Kenya, the formative phase found that phone ownership dropped precipitously before age 18 (youth 17 years and below still had access to and used phones; however they were likely to be shared phones). For privacy purposes, this stage requires participants to have access to their own mobile phones; as such, the age range was set at 18-24 for Kenya. In Peru, where younger youth have access to their own mobile devices, the lower age range of participants is a direct response to recommendations from health- and education- sector stakeholders in Peru, who requested that the ARMADILLO study be directed to a younger age group – specifically, 13-17 year olds – to be more in line with national government programs to reduce adolescent pregnancy. Other eligibility requirements will not change, meaning that 13-17 year olds in Peru will still need to *have their own phone* (and indicate that they control when and with whom they share access). As indicated by young respondents in the formative phase, health-related text messages are common place in Peru; as such receipt of an SRH-related message would not be viewed with trepidation, so long as it appeared to be coming from a trustworthy source.

Sampling strategy

Purposive, snowball sampling will be used to recruit youth for participation in the FGDs, which will be conducted prior to the start of the effect assessment.

For the effect assessment, we will use household-based surveys and multi-stage random sampling. First, recent satellite images or a recent census will be used to identify and enumerate all of the households in the study zone. A random sample of blocks of households will then be selected. Next, the research team will carry out a census of households to enumerate all eligible (those meeting the selection criteria for this study) youth in each selected block.

Second, a member of the study team will randomly select a list of potential participants to be sampled. To minimize contamination, only one youth from each household will be able to participate in this study. The list will be a random generation of 1) the household to be sampled and 2) one individual, eligible youth to be recruited from within that household. As an equal number of males and females from each age range is desired (e.g. for Peru, 72 females aged 13 split evenly across the three arms, 72 males aged 13 split evenly across the three arms, etc), this list will be stratified by sex and age to ensure that there are adequate numbers of eligible youth for each age-sex cohort (there are 10 groups for Peru, considering an age-sex cohort range of 13-17 year-old males and females; there are 14 for Kenya, considering an age-sex cohort range of 18-24 year-old males and females)

These lists will then be recombined into one master list of eligible participants for recruitment purposes. Data collectors (none of whom will be involved in the randomization process described

For peer review only - http://bmjopen.bmj.com/site/about/guidelines.xhtml

above) will be sent to a specific geographic area with a list of 1) the household 2) the individual within this household selected for recruitment. Upon visiting the household, the individual will be consented on the spot. In the case of Peru, parent/guardian consent will also be obtained at this point. If that individual does not wish to participate, no other eligible member of the household may be substituted. If the youth selected is not at home (and/or, in the case of Peru, the parent/guardian is not at home), we will make an appointment for a second visit.

Following enrolment, the phone number of each consented individual will be randomized into one of three arm using block randomization, with block sizes of 6, applied to the individual age-sex cohort lists. The process will continue until the sample size is achieved.

The allocation of participants into intervention, control, or Arm 3 groups (see Table 1 below) will be randomly determined but designed to achieve balance between the arms. Following enrolment of a participant into the study, an algorithm will determine to which study arm the participant's phone number is subscribed. The subscription will be monitored either by the technology partner or a member of the research team not involved in enrolling participants. If allocated to the intervention arm or Arm 3, the participant will receive their first interaction from the appropriate system the following day. If allocated to the control arm, the participant will receive no interactions. Allocation to each arm based on the following characteristics will be assessed at least weekly to ensure balance: age, sex, and years of education.

Study sample size

Table 1 Study Sample Size and a Method

	**Method**	**No. of Participants (Peru)**	**No. of Participants (Kenya)**	**Participants**
**Stage II**	Focus Group Discussions	48 max (up to 6 FGDs with 6-8 participants)	48 max (up to 6 FGDs with 6-8 participants)	In each site: 3 FGDs for males, 3 for females
	Intervention arm<br>• In-Depth Interview	240<br>10	252<br>20	Equal numbers youth males + females
	Control arm	240	252	
	Arm 3 (Contact/Push)<br>• In-Depth Interview	240<br>10	252<br>0	

For Peru, we propose a maximum of 720 participants, split evenly across intervention, control, and push arms. For Kenya, we propose a maximum of 756 participants, split evenly across intervention, control, and contact arms. A sample size of 705 participants per site will provide 80% power to detect a 10% change in mean number of myths believed from baseline to endline, assuming that baseline level of belief is 55%(30)and accounting for a dropout rate of up to 20% in each site. The sample size is calculated to allow for pairwise comparisons between all three

For peer review only

For peer review only - http://bmjopen.bmj.com/site/about/guidelines.xhtml

Adolescent/Youth Reproductive Mobile Access and Delivery Initiative for Love and Life Outcomes (ARMADILLO) protocol for research trial evaluating youth learning and information retention following delivery of SRH information via mobile phones.

groups. The slight increases in sample sizes in each site are to allow for the same sample size across site-specific age groups as well as for males and females.

Data management

All study results will be kept confidential by the team in either password-protected files for electronic data or locked cabinets for paper data. Only approved team members will have access to study results.

A master list will be maintained that includes ID numbers that are uniquely assigned to each participant. Interview notes, consent forms, and digital files will be labelled only with these ID numbers. These master ID lists and informed consent forms will be stored together in a locked cabinet. Master ID lists and informed consent forms will be kept separately from any printed data related to the study (e.g. interview notes). All data collected (digital or paper copy) will be marked with the ID number of the relevant participant – this will be the only unique identifier for any data. All hard copy documents that contain study results will be stored in a locked file cabinet (separate from the Master ID lists) that is accessible only to key study personnel. Digital data files will be stored securely on a password-protected computer and on password-protected cloud storage such as Dropbox. The original FGD and IDI audio recordings will be destroyed after two years while additional transcriptions and study materials will be destroyed after one additional year (the time anticipated for the remainder of the full ARMADILLO study, including the future Stage 3 coverage assessment). The study coordinator for each site, under guidance of the PI, will be tasked with ensuring that all files (hard and digital) have been deleted at the appropriate time. Only the study coordinator and PI for each site will initially have access to the locked cabinets and password protected storage devices used in this study. If both PI and study coordinator are in agreement, access to data files on cloud storage can be granted to select research staff who will be participating in the data analysis.

Any data related to the ARMADILLO system (intervention, control, and arm 3) will be stored on an instance of RapidPro, hosted by the technology partner, Ona. RapidPro, the open source communication platform of choice for this intervention, has the ability to passively track participants' progression through various domains and message content. Data is available on what content an individual phone number accesses and how often. Tracking phone numbers also offers an unobtrusive mechanism for reducing contamination, allowing the research team to monitor the intervention to ensure that the only phone numbers accessing the content of the intervention and contact arms are those associated participants randomized to those respective arms. It should be noted, however, that within the ARMADILLO system, these phone numbers will not be linked with any identifying information on the participant. Additionally, study teams retain complete ownership of the data and of the account(s) on RapidPro. As agreed with Ona, only the study team will be able to grant access to the account to others, including Ona. Having complete ownership of the account, the study teams will be able to completely delete their own data once the project is finished. For more information, please see Ona's data privacy policy here: https://ona.io/privacy.html.

It should be noted that while the intervention makes use of mobile network operator (MNO) infrastructure (with text messages being relayed from RapidPro, likely through an aggregator, to participants' phones) there are no special privacy or confidentiality concerns to users arising from their respective carrier. MNOs in each country will have no way of knowing the content of the

For peer review only - http://bmjopen.bmj.com/site/about/guidelines.xhtml

messages being sent to participants. MNOs will have no more ability to monitor users who happen to be ARMADILLO participants than they would regular users.

Statistical analysisAll interview data collected as part of the qualitative component of this study will be transcribed in the language it was conducted in and then (if necessary) translated into English verbatim and transferred into an electronic file containing one transcript for each data collection event. The interviewers will use these digital files and interview notes taken during data collection to write summary "topline" reports of study results. Reports will contain information on participants' experiences and opinions using the system; recommendations for system improvement; if and with whom they shared messages; and perceived ease or difficulty in translating knowledge obtained through ARMADILLO into action (if action was desired or necessary).

The primary outcome, dispelling myths and misconceptions about contraception, will be assessed using an index of contraception myths and misconceptions(30) developed from relevant literature and the planned Focus Group Discussions. Those in the intervention arm are hypothesized to believe fewer contraception myths than those in control arm and Arm 3 and should therefore have a significantly lower index score following the intervention than they did at baseline and compared to endline assessments of the other two groups.

After assessment of the randomization of participants, if there are no differences between arms in terms of sociodemographic characteristics, differences between arms in the contraception myths index score at endline will be assessed using standard methods—comparisons of proportions(chi-square tests) and means (t-tests) between arms and difference-in-difference techniques. If the randomization was not successful, statistical adjustment with multivariable regression will be used to assess intervention impact. It is envisioned that analyses will be conducted as intent-to-treat. Separately, similar secondary analyses will be conducted on knowledge gained and attitudes shifted in other SRH domains assessed, as well as the retention of both primary and secondary outcomes over time (through the follow-up period).

Significance to the sexual- and reproductive health and rights research area

Part of the Sustainable Development Goal commitment towards *ensuring health lives and promoting well-being for all at all ages* includes a commitment to '*ensuring universal access to sexual health and reproductive health-care services, including for family planning, information and education…'*. As mentioned above, young populations face added challenges to accessing the SRH information and services to which they are entitled. Interventions like ARMADILLO use digital health innovation to fill the commitment to *universal* access to SRH information and education for an otherwise difficult-to-reach group.

As the global ARMADILLO study is situated in two sites (Peru and Kenya), the results of this phase are applicable to understanding the potential of digital health technologies to positively influence SRH learning in youth. The initial formative phase allowed us to develop a technological tool that will potentially empower youth to be more knowledgeable with regards to their sexual and reproductive health. As previously mentioned, the platform uses SMS (text message) technology, taking advantage of a base channel of mobile communication that is readily available across virtually all settings where mobile phones are available. However, while use of SMS to deliver information to young people is a popular strategy, it has yet to be rigorously evaluated. The proposed study design will contribute needed data in this regard.

For peer review only - http://bmjopen.bmj.com/site/about/guidelines.xhtml

Adolescent/Youth Reproductive Mobile Access and Delivery Initiative for Love and Life Outcomes (ARMADILLO) protocol for research trial evaluating youth learning and information retention following delivery of SRH information via mobile phones.

Additionally, this evaluation is strategically positioned to take place prior to the start of a broad rollout of the full ARMADILLO architecture and content and a corresponding assessment of the system's coverage. Assessing the learning potential of the system before the rollout has a few important results. First, the chance for contamination between arms is reduced (there are no promotional materials or campaigns that could contaminate Arm 3 or control participants). Second (and most important), conducting the impact assessment first allows for a 'proof of concept' approach to the broader ARMADILLO study – prove first that ARMADILLO can make a difference, then roll it out broadly.

END RESEARCH PLAN

Annexes to research plan:
Annex 1: Study tools (generic tools, to be streamlined for each site, based on participant age and country context) – **Table 2** (below) contains full list of study tools
Annex 2: ARMADILLO draft message content (developed with and vetted by youth in Stage I)
Annex 3: Informed consent forms (Kenya and Peru site forms) – **Table 2** (below) contains full list of ICFs)
Annex 4: Budget narratives for ARMADILLO Kenya and Peru sites

Table 2: Study Content and Informed Consent Tools

<table>
<tr><th>Study Tool</th><th>Consent/Assent</th><th>Participant description</th></tr>
<tr><td>Annex 1
Tool 1: FGD Guide</td><td>Annex 3
Youth 18-24 (Kenya):
Consent Form 1
Annex 5
Youth 13-17 (Peru):
Formulario 1
Parental permission Formulario 2</td><td rowspan="4">Youth 13-24 years</td></tr>
<tr><td>Annex 1
Tool 2: Baseline/Endline/Follow-up Survey</td><td rowspan="2">Annex 3
Youth 18-24 (Kenya):
Consent Form 4

Annex 5
Youth 13-17 (Peru):
Formulario 3
Parental Permission Formulario 4</td></tr>
<tr><td>Annex 2
Study Content 1:
Bank of SMS message content, developed in ARMADILLO Stage I</td></tr>
<tr><td>Annex 1
Tool 3:
In-Depth Interview Guide</td><td>Annex 3
Youth 18-24 (Kenya):
Consent Form 7

Annex 5:
Youth 13-17 (Peru):</td></tr>
</table>

For peer review only

For peer review only - http://bmjopen.bmj.com/site/about/guidelines.xhtml

Adolescent/Youth Reproductive Mobile Access and Delivery Initiative for Love and Life Outcomes (ARMADILLO) protocol for research trial evaluating youth learning and information retention following delivery of SRH information via mobile phones.

	Formulario 3<br>Parental Permission Formulario 4	

Project organisation

WHO secretariat will maintain the coordination structure established for ARMADILLO Stage 1 (described again below) for guidance across sites and ensure activities are implemented according to the research plan.

Multi-site research coordination

WHO Department of Reproductive Health and Research (RHR/HRP) will serve as the coordinating body for the research, with the Principal Research Coordinator situated in HRP, and provide oversight to alignment and coordination of goals and activities across study sites. A primary responsibility will be to ensure integrity of study research implementation, research questions, and study instruments and coordinate analyses across sites.

Research partners

The in-country research lead in Kenya is the **International Centre for Reproductive Health – Kenya (ICRH-K)**, a local non-governmental organization registered in Kenya in 2000. ICRH-K has been involved in numerous operations and clinical-based research, in addition to health project implementation, in the areas of reproductive health and gender-based rights for Kenya. ICRH-K seeks to improve the acceptability, accessibility and quality of sexual and reproductive health services, and integrate a human right based and gender sensitive approach in its analysis. As the in-country research lead, ICRH-K will participate in protocol development, lead the training of the field research teams, and lead implementation of this research protocol in Kwale County. ICRH-K will also lead the analysis of Kenya-site data from the research study. ICRHK will also be responsible for building and maintaining relationships with relevant community groups throughout the duration of the study, as well as engaging with relevant stakeholders (including local and county health officials, in the case of Kenya) for dissemination of the study's results.

The in-country research lead in Peru is the **Universidad Peruana Cayetano Heredia (UPCH)**, a local non-profit university founded in Peru in 1961. UPCH has led and been involved in numerous public health research projects on numerous topics, including sexual and reproductive health and the prevention of HIV and other sexually transmitted infections. As the in-country research lead, UPCH will participate in protocol development, lead the training of the field research teams, lead implementation of this research protocol in Lima, and lead the analysis of Peru-site data from the research study. UPCH will also be responsible for building and maintaining relationships with relevant community groups throughout the duration of the study, as well as engaging with relevant stakeholders (including Ministry of Health officials, in the case of Peru) for dissemination of the study's results.

For both research implementing partners, the principal investigator will be the head of the research team, providing technical assistance and ensuring protocol fidelity form the start to the end of the study. The PI will also lead site-specific data analysis for study findings. The study coordinator, under the guidance of the PI, will lead the recruitment process for research assistants and data collectors.

For peer review only - http://bmjopen.bmj.com/site/about/guidelines.xhtml

Adolescent/Youth Reproductive Mobile Access and Delivery Initiative for Love and Life Outcomes (ARMADILLO) protocol for research trial evaluating youth learning and information retention following delivery of SRH information via mobile phones.

Data collector recruitment will follow each organization's policy of advertising, shortlisting and interviewing the shortlisted candidates through a rigorous process to ensure they meet the required standards. Data collectors will assist with the recruitment of study participants; they will conduct FGDs; IDIs; key informant interviews; and administer study questionnaires. Research assistants will be university students or recent graduates (to maintain age-similarity with participants).

All data collectors will undergo training in each of the study methods required for this study as well as the ethics of human subjects research. Data collectors will also be trained on how to recruit and consent participants. All data collectors will be familiarized with the content of the messages (domains and sub-domains, tone of messaging) and - as mentioned elsewhere in this protocol – will be informed as to the supportive services that are available for particular areas (gender-based violence, for example). On a day-to-day basis, data collectors will report to the study coordinator, who will in turn keep the PI informed of study progress. Form III (budget) specifies additional members of the study team who – while not involved in the data collection – will be involved in the overall administration and monitoring and evaluation of the study (roles are specified in Form III).

Technology partner

Ona has been identified to serve as the technology and implementation partner. Ona is a technology company based in Nairobi, Kenya and New York City, NY, USA. Ona makes tools that allow organizations to make better use of their data to help address some of the world's great challenges, including: child and maternal health, education, governance, agriculture, access to infrastructure and government accountability. Their team has been at the forefront of information and communications technology (ICT) solutions in the global development space for the past 10 years. This includes projects implemented on the ground in: Kenya, Senegal, Mali, Ghana, Libya, Nigeria, Malawi, Uganda, Tanzania, Rwanda, Ethiopia, Indonesia, Pakistan, Bangladesh, Vietnam and India.

Ona's role in this project will be to set up the SMS communication platform and to develop the randomization algorithm and flows for segmenting research participants into topical domain sequences. Ona will also provide technical support throughout the project. The ARMADILLO system will be built off of the RapidPro platform. Launched by UNICEF, RapidPro is an open-source platform of applications that can help governments deliver rapid and vital real-time information and connect communities to lifesaving services.

Timeline:

ARMADILLO Stage 2 is estimated to take approximately 12 months. The initial three months will involve preparatory activities, including:

- obtaining ERC approval and local IRB approval;
- recruiting and training data collectors;
- developing the technology architecture of the Stage 2 intervention/contact/control (inputting message content, developing message flows, obtaining short codes from mobile network operators

FGDs prior to the start of the intervention are expected to take a maximum of two months, which includes:

- recruitment, scheduling, and conducting the FGDs

For peer review only - http://bmjopen.bmj.com/site/about/guidelines.xhtml

- rapid analysis of results to incorporate any unexpected findings into messages/study instrument as necessary

The intervention itself is anticipated to last an additional five months, which includes:

- ~1 month for full recruitment
- Two months for the intervention itself
- An additional two months until the last-enrolled participant completes their two-month follow-up visit

Finally, an additional two months will be spent on reporting and dissemination of the Stage 2 research results.

Please see Form IV for the detailed Gantt chart.

Budget justification

Form III contains budgets for ARMADILLO's two research partners as well as the technology partner. A budget narrative for research implementing partners UPCH and ICRHK is included as Annex 4.

References:

1. UNFPA. ADOLESCENT AND YOUTH DEMOGRAPHICS : A BRIEF OVERVIEW. 2010:4-5.
2. Oringanje C, Meremikwu MM, Eko H, Esu E, Meremikwu A, Ehiri JE. Interventions for preventing unintended pregnancies among adolescents. The Cochrane database of systematic reviews 2009:CD005215.
3. World Health Organization. Early marriages, adolescent and young pregnancies: Report by the Secretariat. Geneva: Sixty-Fifth World Health Assembly: provisional agenda item 13.4 A65/13. In; 2012; Geneva; 2012. p. 16-19.
4. Department of Child and Adolescent Health and Development. Global consultation on adolescent friendly health services: A consensus statement. Geneva; 7-9 March 2001.
5. Svanemyr J, Amin A, Robles OJ, Greene ME. Creating an Enabling Environment for Adolescent Sexual and Reproductive Health: A Framework and Promising Approaches. Journal of Adolescent Health;56(1):S7-S14.
6. Interagency Youth Working Group. Mobile Technology for Health. In; 2013; 2013.
7. Statistics KNBo. Population and Housing Census 2009. In; 2009; Nairobi; 2009.
8. Kalamar AMT, Özge; Hindin, Michelle J. . Developing Strategies to Address Contraceptive Needs of Adolescents: Exploring Patterns of Use Among Sexually Active Adolescents in 45 Low- and Middle-Income Countries. In: 14th Congress - 2nd Global Conference - of the European Society of Contraception and Reproductive Health; 2016; Basel, Switzerland; 2016.
9. Fjeldsoe BS, Marshall AL, Miller YD. Behavior change interventions delivered by mobile telephone short-message service. American journal of preventive medicine 2009;36:165-73.
10. Krishna S, Boren SA, Balas EA. Healthcare via cell phones: a systematic review. Telemedicine journal and e-health : the official journal of the American Telemedicine Association 2009;15:231-40.
11. Labrique AB, Vasudevan L, Kochi E, Fabricant R, Mehl G. mHealth innovations as health system strengthening tools: 12 common applications and a visual framework. Global Health: Science and Practice 2013;1:160-171.

For peer review only - http://bmjopen.bmj.com/site/about/guidelines.xhtml

Adolescent/Youth Reproductive Mobile Access and Delivery Initiative for Love and Life Outcomes (ARMADILLO) protocol for research trial evaluating youth learning and information retention following delivery of SRH information via mobile phones.

12. Lim MSC, Hocking JS, Hellard ME, Aitken CK. SMS STI: a review of the uses of mobile phone text messaging in sexual health. International journal of STD & AIDS 2008;19:287-90.
13. Kay M, Santos J, Takane M. mHealth: New horizons for health through mobile technologies. In: World Health Organization; 2011; 2011.
14. Free C, Phillips G, Galli L, Watson L, Felix L, Edwards P, et al. The effectiveness of mobile-health technology-based health behaviour change or disease management interventions for health care consumers: a systematic review. PLoS medicine 2013;10:e1001362.
15. Cole-Lewis H, Kershaw T. Text messaging as a tool for behavior change in disease prevention and management. Epidemiologic reviews 2010;32:56-69.
16. Gurman TA, Rubin SE, Roess AA. Effectiveness of mHealth behavior change communication interventions in developing countries: a systematic review of the literature. Journal of health communication 2012;17 Suppl 1:82-104.
17. Head KJ, Noar SM, Iannarino NT, Grant Harrington N. Efficacy of text messaging-based interventions for health promotion: a meta-analysis. Social science & medicine (1982) 2013;97:41-8.
18. de Tolly K, Skinner D, Nembaware V, Benjamin P. Investigation into the use of short message services to expand uptake of human immunodeficiency virus testing, and whether content and dosage have impact. Telemed J E Health 2012;18(1):18-23.
19. Levine D, McCright J, Dobkin L, Woodruff AJ, Klausner JD. SEXINFO: a sexual health text messaging service for San Francisco youth. American journal of public health 2008;98:393-5.
20. Wireless Quick Facts. The International Association for the Wireless Telecommunications Industry (CTIA) 2013.
21. In U.S., SMS Text Messaging Tops Mobile Phone Calling. The Nielsen Company 2008.
22. Al-Shorbaji N, Geissbuhler A. Establishing an evidence base for e-health: the proof is in the pudding. Bulletin of the World Health Organization 2012;90:322-322A.
23. Kumar S, Nilsen WJ, Abernethy A, Atienza A, Patrick K, Pavel M, et al. Mobile health technology evaluation: the mHealth evidence workshop. American journal of preventive medicine 2013;45:228-36.
24. County Government of Kwale. First county integrated development plan 2013. In; 2013.
25. Kenya Ministry of Health. Kwale county: Health at a glance; 2015.
26. Kenya National Bureau of Statistics. Kenya Demographic and Health Survey 2014. Rockville, Maryland: The DHS Program, ICF International; 2015.
27. Kenya National Bureau of Statistics. Access to Mobile Phone Service by County and District; 2009.
28. Premium Youth Research — mobileYouth® - youth marketing and mobile culture.
29. Municipalidad de San Juan de Miraflores. Geografía In.
30. Gueye A, Speizer IS, Corroon M, Okigbo CC. Belief in Family Planning Myths at the Individual and Community Levels and Modern Contraceptive Use in Urban Africa. International Perspectives on Sexual and Reproductive Health 2015;41(4):191-9.

Ethical considerations and core values

Those who voluntarily consent to participate in the study will be fully informed, as part of the consent process, that the study concerns SRH topics that they may consider sensitive and that it is possible that questions in the survey or message content could make the respondents feel uncomfortable. Participants will be informed as part of the consent process that they can choose

For peer review only - http://bmjopen.bmj.com/site/about/guidelines.xhtml

Adolescent/Youth Reproductive Mobile Access and Delivery Initiative for Love and Life Outcomes (ARMADILLO) protocol for research trial evaluating youth learning and information retention following delivery of SRH information via mobile phones.

to ignore questions or leave the study altogether at any point if they feel uncomfortable, without repercussion. In cases where participants under the age of 18 are recruited, parental consent will also be obtained prior to study enrolment.

Data collectors for (administering surveys and the in-depth interviews) will be the same sex of participants, in order to minimize any discomfort participants may have commenting on the subject matter. Participants will be encouraged to identify an environment in which they are comfortable speaking with the research team, and at a time that suits them.

Ethical challenges

The ARMADILLO text messaging service is an innovative, private, and easily accessible method for providing sexual and reproductive health information to young people. There are several limitations to this program, however. First, to use the service, users must own or have access to a mobile phone and be familiar with text messaging. Second, users must have a basic level of literacy to read the text messages; however, literacy rates in Kenya and Peru are relatively high so it is expected that most people will be able to read and understand the message content. Third, the service provides cursory information on the included topics and the user will need to access a different source for in-depth information and/or to take action.

One notable exception are the messages on gender-based violence, which will contain direct linkages to site-specific resources from which to seek immediate support. Both Peru and Kenya have short codes linking to 24-hour hotlines available to victims of violence. Additionally, both study sites have GBV-services in the catchment area. As such, messages in the violence module will provide the relevant hotline phone number and information on where participants can seek services if needed/desired. Prior to the start of the study, each research team will liaise with GBV support and counselling centres in their respective catchment areas to be sensitized to the services available. Then, at the point of consenting, all participants will be provided with a discrete (e.g. credit-card-sized) card that also contains the list of relevant hotlines and GBV-services in the catchment area.

For the purposes of data collection, in this stage, users must also receive occasional messages pushed to their phone (quizzes and topic/unlocked domain alerts), thereby sacrificing a small modicum of privacy that a purely on-demand system would offer. In order to compensate, eligibility criteria require that participants in this phase have their own mobile devices, addressing a concern of an SMS appearing on a shared phone when the study participant is not using it. Learnings from similar programs have indicated that mobile phone users are adept at managing their text messages to maintain sufficient privacy in their communications.

A common concern in these types of interventions is whether there is a privacy-related risk to participants, in that an outside person (parent/caregiver, partner, friend) may view the messages on the participant's phone against their wishes. This was a concern first flagged during the formative stage of research (see summary of Stage 1 findings at the beginning of this proposal) and so was thoroughly explored over the course of data collection.

In both sites, formative data indicates that even if partners, peers and/or parents were to come across information from the study, there would be minimal – if any – repercussions. In Kenya, rather, parents, caregivers, and stakeholders engaged with the formative phase confirmed that their communities would view mobile phones as a valuable means of providing SRH information to young people. Meanwhile, in Peru, respondents indicated that health-oriented message

For peer review only - http://bmjopen.bmj.com/site/about/guidelines.xhtml

campaigns were common place and would be acceptable so long as they appeared as 'public service messages'. While privacy concerns are anticipated to be a non-issue, should we find otherwise over the course of Stage 2 data collection, the study will not continue.

Incentives
For all in-person interactions (initial recruitment, completion of end-line survey, and follow-up activities), participants will be reimbursed for travel costs as is appropriate at the time research activities are conducted. For in-person data collection, participants may also be provided with beverage and/or food during the data collection.

Participants will be reminded at many points throughout the intervention that their participation will not incur any SMS charges. Additionally, in order to combat traditional loss-to-follow-up and engagement-drop-off that digital health interventions face, as well as to reinforce the concept that study participation is free, all participants in the intervention arm and Arm 3 in both sites will be given token amounts of phone credit for responding to the study's quiz questions delivered via SMS. Any response (not just correct responses) will receive phone credit.

Small phone credit disbursements such as those proposed above are typical in this research context and appropriate in these settings considering what is being asked of the participants. Travel reimbursement mentioned above is intended to make study participation affordable, but not coercive, to all eligible participants.

Benefits to participation in this study are the opportunity to learn about youth sexual and reproductive health. Intervention participants will have access to ARMADILLO message content while control participants will be encouraged to explore and research SRH domains they may not have traditionally had information on. At the end of the full intervention and follow-up period, the full ARMADILLO system will be made available to all study participants (intervention, contact, and control).

With regards to the study methods themselves, all information collected will remain confidential with the study team.

Gender and social equity aspects
With regards to study participants and research questions, digital health communication interventions such as ARMADILLO are designed to be accessible to both young males and females. The channel of SMS was also selected because of its universality in mobile phones, creating no socio-economic barrier to participating in this study (between those with the money and ability to purchase more costly smartphones and those with older, feature phones). The developed content was vetted with approximately equal numbers of males and females in each site, so that these messages might resonate with and be relatable to both groups. As for the research team both research partners will be encouraged to recruit equal numbers of male and female researchers to their study team.

Community Participation
Mechanisms to ensure community participation were established during the first stage of research in order to ensure buy-in with both the concept of the study as well as the messages themselves. **Kenya:** Community participation comes from the work of the ARMADILLO Community Advisory

For peer review only - http://bmjopen.bmj.com/site/about/guidelines.xhtml

Adolescent/Youth Reproductive Mobile Access and Delivery Initiative for Love and Life Outcomes (ARMADILLO) protocol for research trial evaluating youth learning and information retention following delivery of SRH information via mobile phones.

Board (CAB), the role of which is to act as a liaison between the community and the research team. The members of the CAB collaborate with the research team, on behalf of the community in achieving relevant, acceptable, and ethical research that focuses on the lives and issues of the community where the study is conducted. The ARMADILLO CAB in Kenya comprised of the representatives from youth groups, youth led NGOs, representative from county department of health, representative from county department of education, youth affairs, village chief and ARMADILLO study coordinator.

Peru: During Stage II (and building off of community sensitization efforts established in Stage I as well as UPCH's standard community-engagement practices) UPCH will hold a community meeting in the study site area (Pampas) and, similarly to Kenya, establish a Community Advisory Board whose composition will reflect key youth-related stakeholder groups. For the Pampas community, - in addition to youth themselves – this will also include representatives from secondary schools, health centres with adolescent-targeted services, and churches.

Research Capacity Strengthening

We are working with two research partners from academic settings who have some, but relatively limited experiences in trial methodology for social science interventions and outcomes. We envision joint analyses and collaborative research papers as well as shared learning opportunities between the sites and WHO-HQ.

For peer review only

For peer review only - http://bmjopen.bmj.com/site/about/guidelines.xhtml

CONSORT 2010 checklist of information to include when reporting a randomised trial*

Section/Topic	Item No	Checklist item	Reported on page No
**Title and abstract**			
	1a	Identification as a randomised trial in the title	1
	1b	Structured summary of trial design, methods, results, and conclusions (for specific guidance see CONSORT for abstracts)	1-2
**Introduction**			
Background and objectives	2a	Scientific background and explanation of rationale	3-4
	2b	Specific objectives or hypotheses	4
**Methods**			
Trial design	3a	Description of trial design (such as parallel, factorial) including allocation ratio	4
	3b	Important changes to methods after trial commencement (such as eligibility criteria), with reasons	N/A
Participants	4a	Eligibility criteria for participants	5
	4b	Settings and locations where the data were collected	5
Interventions	5	The interventions for each group with sufficient details to allow replication, including how and when they were actually administered	5-6
Outcomes	6a	Completely defined pre-specified primary and secondary outcome measures, including how and when they were assessed	6-7
	6b	Any changes to trial outcomes after the trial commenced, with reasons	N/A
Sample size	7a	How sample size was determined	7
	7b	When applicable, explanation of any interim analyses and stopping guidelines	N/A
Randomisation:			
Sequence generation	8a	Method used to generate the random allocation sequence	7
	8b	Type of randomisation; details of any restriction (such as blocking and block size)	7
Allocation concealment mechanism	9	Mechanism used to implement the random allocation sequence (such as sequentially numbered containers), describing any steps taken to conceal the sequence until interventions were assigned	7
Implementation	10	Who generated the random allocation sequence, who enrolled participants, and who assigned participants to interventions	7
Blinding	11a	If done, who was blinded after assignment to interventions (for example, participants, care providers, those	N/A

For peer review only

		assessing outcomes) and how	
	11b	If relevant, description of the similarity of interventions	6
Statistical methods	12a	Statistical methods used to compare groups for primary and secondary outcomes	7-9
	12b	Methods for additional analyses, such as subgroup analyses and adjusted analyses	9
**Results**			
Participant flow (a diagram is strongly recommended)	13a	For each group, the numbers of participants who were randomly assigned, received intended treatment, and were analysed for the primary outcome	10-11(Figure 1)
	13b	For each group, losses and exclusions after randomisation, together with reasons	(Figure 1)
Recruitment	14a	Dates defining the periods of recruitment and follow-up	(Figure 1)
	14b	Why the trial ended or was stopped	N/A
Baseline data	15	A table showing baseline demographic and clinical characteristics for each group	(Table 1)
Numbers analysed	16	For each group, number of participants (denominator) included in each analysis and whether the analysis was by original assigned groups	(Table 1)
Outcomes and estimation	17a	For each primary and secondary outcome, results for each group, and the estimated effect size and its precision (such as 95% confidence interval)	13-15 (Table 2 and 3)
	17b	For binary outcomes, presentation of both absolute and relative effect sizes is recommended	N/A
Ancillary analyses	18	Results of any other analyses performed, including subgroup analyses and adjusted analyses, distinguishing pre-specified from exploratory	12-13 (Table 2 and 3)
Harms	19	All important harms or unintended effects in each group (for specific guidance see CONSORT for harms)	N/A
**Discussion**			
Limitations	20	Trial limitations, addressing sources of potential bias, imprecision, and, if relevant, multiplicity of analyses	18-19
Generalisability	21	Generalisability (external validity, applicability) of the trial findings	17-18
Interpretation	22	Interpretation consistent with results, balancing benefits and harms, and considering other relevant evidence	19
**Other information**			
Registration	23	Registration number and name of trial registry	10
Protocol	24	Where the full trial protocol can be accessed, if available	Cited (ref: 26)
Funding	25	Sources of funding and other support (such as supply of drugs), role of funders	19

*We strongly recommend reading this statement in conjunction with the CONSORT 2010 Explanation and Elaboration for important clarifications on all the items. If relevant, we also recommend reading CONSORT extensions for cluster randomised trials, non-inferiority and equivalence trials, non-pharmacological treatments, herbal interventions, and pragmatic trials. Additional extensions are forthcoming: for those and for up to date references relevant to this checklist, see www.consort-statement.org.

For peer review only - http://bmjopen.bmj.com/site/about/guidelines.xhtml

BMJ Open

Busting contraception myths and misconceptions among youth in Kwale County, Kenya: Results of a digital health randomized control trial

Journal:	*BMJ Open*
Manuscript ID	bmjopen-2020-047426.R1
Article Type:	Original research
Date Submitted by the Author:	21-Sep-2021
Complete List of Authors:	Gichangi, Peter; Technical University of Mombasa, Administration; International Centre for Reproductive Health Kenya<br>Gonsalves, Lianne; World Health Organization, Department of Sexual and Reproductive Health and Research including UNDP/UNFPA/UNICEF/WHO/World Bank Special Programme of Research, Development and Research Training in Human Reproduction (HRP)<br>Mwaisaka, Jefferson; International Centre for Reproductive Health Kenya<br>Thiongo, Mary; International Centre for Reproductive Health Kenya<br>Habib, Ndema; World Health Organization, Department of Sexual and Reproductive Health and Research including UNDP/UNFPA/UNICEF/WHO/World Bank Special Programme of Research, Development and Research Training in Human Reproduction (HRP)<br>Waithaka, Michael; International Centre for Reproductive Health Kenya,<br>Tamrat, Tigest ; World Health Organization, Department of Sexual and Reproductive Health and Research including UNDP/UNFPA/UNICEF/WHO/World Bank Special Programme of Research, Development and Research Training in Human Reproduction (HRP)<br>Agwanda, Alfred ; University of Nairobi<br>Sidha, Hellen; National Council for Population and Development<br>Temmerman, Marleen; Aga Khan University - Kenya; International Centre for Reproductive Health<br>Say, Lale; World Health Organization, Department of Sexual and Reproductive Health and Research including UNDP/UNFPA/UNICEF/WHO/World Bank Special Programme of Research, Development and Research Training in Human Reproduction (HRP)
**Primary Subject Heading**:	Public health
Secondary Subject Heading:	Sexual health, Research methods
Keywords:	Reproductive medicine < GYNAECOLOGY, PUBLIC HEALTH, SEXUAL MEDICINE, Clinical trials < THERAPEUTICS

SCHOLARONE™
Manuscripts

I, the Submitting Author has the right to grant and does grant on behalf of all authors of the Work (as defined in the below author licence), an exclusive licence and/or a non-exclusive licence for contributions from authors who are: i) UK Crown employees; ii) where BMJ has agreed a CC-BY licence shall apply, and/or iii) in accordance with the terms applicable for US Federal Government officers or employees acting as part of their official duties; on a worldwide, perpetual, irrevocable, royalty-free basis to BMJ Publishing Group Ltd ("BMJ") its licensees and where the relevant Journal is co-owned by BMJ to the co-owners of the Journal, to publish the Work in this journal and any other BMJ products and to exploit all rights, as set out in our licence.

The Submitting Author accepts and understands that any supply made under these terms is made by BMJ to the Submitting Author unless you are acting as an employee on behalf of your employer or a postgraduate student of an affiliated institution which is paying any applicable article publishing charge ("APC") for Open Access articles. Where the Submitting Author wishes to make the Work available on an Open Access basis (and intends to pay the relevant APC), the terms of reuse of such Open Access shall be governed by a Creative Commons licence – details of these licences and which Creative Commons licence will apply to this Work are set out in our licence referred to above.

Other than as permitted in any relevant BMJ Author's Self Archiving Policies, I confirm this Work has not been accepted for publication elsewhere, is not being considered for publication elsewhere and does not duplicate material already published. I confirm all authors consent to publication of this Work and authorise the granting of this licence.

For peer review only

Busting contraception myths and misconceptions among youth in Kwale County, Kenya: Results of a digital health randomized control trial

Gichangi Peter, PhD*[1,2,3], Gonsalves Lianne, PhD*[4,5,6], Mwaisaka Jefferson M. A[1,7], Thiongo Mary, MSC[1], Habib Ndema, PhD[4], Waithaka Michael, MSC[1], Tamrat Tigest, MPH[4], Agwanda Alfred, PhD[8], Sidha Hellen, MPH[9], Temmerman Marleen, PhD[1,3,10], Say Lale, MD[4].

***These authors have equal contribution**

[1]International Centre for Reproductive Health, Kenya; [2]Technical University of Mombasa; [3]Department of Public Health and Primary Care, Faculty of Medicine and Health Sciences, Ghent University, Belgium; [4]Department of Sexual and Reproductive Health and Research including UNDP/UNFPA/UNICEF/WHO/World Bank Special Programme of Research, Development and Research Training in Human Reproduction (HRP), World Health Organization, Geneva, Switzerland, [5]Swiss Tropical and Public Health Institute (Swiss TPH), Basel, Switzerland; [6]University of Basel, Basel, Switzerland [7]University of Ghana, College of Health Sciences, Accra Ghana; [8]University of Nairobi; [9]National Council for Population and Development; [10]Aga Khan University, Kenya

Corresponding author:

Prof Peter Gichangi, PhD
Address: P.O Box 2631-00202, Nairobi, Kenya,
email: gichangip@yahoo.com.
Phone: +254 722 521 946

Word Count (excluding abstract, tables, references)

3695

Abstract:

Objectives

The objective of this randomized controlled trial in Kenya was to assess the effect of delivering sexual and reproductive health (SRH) information via text message to young people on their ability to reject contraception-related myths and misconceptions.

Design and Setting

A three-arm, unblinded randomized controlled trial with a ratio of 1:1:1 in Kwale County, Kenya.

For peer review only - http://bmjopen.bmj.com/site/about/guidelines.xhtml

Participants and interventions

A total of 740 youth aged 18-24 years were randomized. Intervention arm participants could access informational SRH text messages on-demand. Contact arm participants received once weekly texts instructing them to study on an SRH topic on their own. Control arm participants received standard care. The intervention period was 7 weeks.

Primary outcome

We assessed change myths believed at baseline and endline using an index of 10 contraception-related myths. We assessed change across arms using difference of difference analysis.

Results

Across arms, <5% of participants did not have any formal education, <10% were living alone, about 50% were single and >80% had never given birth. Between baseline and endline, there was a statistically significant drop in the average absolute number of myths and misconceptions believed by intervention arm (11.1%, 95% CI [17.1%, 5.2%]), contact arm (14.4%, 95% CI [20.5%, 8.4%]) and control arm (11.3%, 95% CI [17.4%, 5.2%]) participants. However, we observed no statistically significant difference in the magnitude of change across arms.

Conclusions

We are unable to conclusively state that the text message intervention was better than text message ‘contact’ or no intervention at all. Digital health likely has potential for improving SRH-related outcomes when used as part of multi-faceted interventions. Additional studies with physical and geographical separation of different arms is warranted.

Trial Registration

ISRCTN85156148

Strengths and limitations

- This study included two digital intervention arms, meaning that it would be possible to determine whether changes in outcomes were due to sexual and reproductive health content delivered by phone, or participants being ‘nudged’ by phone to think about (and learn about) their sexual and reproductive health (SRH).
- The study intentionally did not power sample size around SRH behavioural outcomes, building on previous research that light-touch digital interventions alone may not be enough to see behaviour change in such a complex area of health – instead the primary outcome focuses on SRH knowledge.
- A key limitation is that the study’s individual-level randomization of young people living near each other is likely to have resulted in contamination between arms.

For peer review only - http://bmjopen.bmj.com/site/about/guidelines.xhtml

For peer review only

For peer review only - http://bmjopen.bmj.com/site/about/guidelines.xhtml

Introduction

There is a high unmet need for sexual and reproductive health (SRH) information and services, for both married and unmarried youth worldwide. Data from 61 low and middle-income countries (LMICs) show that 33 million women aged 15–24 have an unmet need for contraception. [1] In Kenya, the 2014 Demographic and Health Survey found that modern contraceptive use among all adolescents age 15-19 years is low (9.3%) compared to all women aged 15-49 years (39.1%). [2] Partly as a result, the number of adolescents aged 15-19 years who were pregnant or mothers has stagnated at 18% between 2008 and 2014. [2, 3]

Sexually active young people face a variety of obstacles to access and use modern contraceptives. They may encounter financial, cultural, social, legal barriers, fear of side effects (e.g. infertility and adverse reactions) or cultural norms that restrict their access to contraception services in health facilities. [4-7] Additionally, contraception myths and misconceptions can negatively affect access to and use of SRH services. [8, 9] Misinformation and myths/misconceptions are often learnt from social networks. [10, 11] In this paper we describe myths and misconceptions as those being communal or widespread beliefs about effects of contraceptives, which are distinct from individuals' experiences with contraception-related side effects.[12]

The proliferation of mobile phone technology, and its popularity and ownership with young people in particular, [13, 14] provides an innovative way to educate young people on contraception and their health more broadly. There are indications that health promotion campaigns among adolescents and young people through text messaging may contribute to improved sexual and reproductive health knowledge, behaviours and outcomes. [15] However, there is less rigorous research and documentation of SRH mobile phone interventions for adolescents and young people in developing

For peer review only - http://bmjopen.bmj.com/site/about/guidelines.xhtml

countries. [16] In Kenya in particular, an estimated 93% of households already owned a mobile phone by 2011. [17]

Mobile phone-based digital health interventions have been successfully used in HIV programs [18, 19], post-abortion care [20], and to address chronic disease conditions. [21] Providing broader SRH content, including contraception information, via mobile phones to young people would appear to be a natural strategy to reach them [22], increase their contraception knowledge [23], and improve correct contraception use.[24] After all, when it comes to 'sensitive' SRH content, mobile phones can privately deliver information without stigma or judgment. However, the evidence that digital health interventions can improve youth SRH-related outcomes, including contraception knowledge and uptake, is yet to be significantly established. [25-27]

To address this gap, the World Health Organization's Department of Sexual and Reproductive Health and Research partnered with research partners in Peru and Kenya to develop the Adolescent/Youth Reproductive Mobile Access and Delivery Initiative for Love and Life Outcomes (ARMADILLO) Study. The ARMADILLO intervention used short message service (SMS, also known as 'text message') to deliver SRH information on-demand via a numbers-based menu. The content was developed in the study's formative stage around several SRH 'domains' of interest to policymakers and young people alike. [28] The intervention was evaluated using a three-arm randomized controlled trial (RCT). This paper reports on the Kenya study's primary outcome: are young people with access to the ARMADILLO intervention better able to reject contraception myths and misconceptions as compared with periodic SMS encouraging self-learning or usual care (no intervention).

For peer review only

For peer review only - http://bmjopen.bmj.com/site/about/guidelines.xhtml

Methods

Study Design

This was a three-arm randomized controlled trial (1:1:1 allocation) involving youth age 18-24 years. The trial ran for seven weeks, with assessments at baseline and endline. The study methods have been described elsewhere in full, [29] but are described briefly below.

Participants and Setting

The study was conducted in Kwale County, one of the six counties in the Coastal region of Kenya. The study area consisted of select enumeration areas (EAs) in six Kwale County sub-locations which border each other: Ngombeni, Kitivo, Simkumbe, Mkoyo, Gombato and Ukunda. Eligibility criteria was as follows: youth (male and female) aged 18-24; literate; had their own mobile phone at the time of recruitment and reported regular use; reported current use of text messaging.

The Kenya National Bureau of Statistics provided a list of EAs for the six sub-locations. From this list, we randomly selected 21 EAs to be mapped. During the mapping (October 2017), data collectors visited all households and enumerated anyone in the home who was age-eligible to participate. Then, we randomly selected one eligible participant from each household. Starting in February 2018, trained data collectors returned to households and attempted to recruit the selected youth. After consenting to participate, participants completed a baseline survey. Both baseline and endline surveys were implemented by trained data collectors, who entered participants' responses into a webform on a tablet. For a few sensitive questions relating to previous contraception use and sexual behaviour, participants entered their responses onto the tablet themselves. Participants were then remotely randomized into one of three arms. Intervention and contact arm participants received their first message the following day.

For peer review only - http://bmjopen.bmj.com/site/about/guidelines.xhtml

Interventions

The interventions were categorized as per WHO classification of digital health interventions. [30] Arm 1 (Intervention arm) was an *on-demand information service to clients (WHO Classification 1.6)*. Participants received access to one domain of ARMADILLO content (e.g. 'puberty and anatomy' or 'pregnancy prevention') each week and could request any 'sub-domain' that interested them from the menu (e.g. 'menstruation' or 'physical changes' for the puberty and anatomy domain or 'implants' or 'male condoms' for the pregnancy prevention domain) for free for the entire week (see Suppl Figure 1). Arm 2, (Contact arm) employed *targeted client communication (WHO Classification 1.1)*. Participants received the same system-initiated contacts as Arm 1 participants but without access to the ARMADILLO content itself. Instead, a once-weekly SMS alerted them to an SRH domain for that week (e.g. relationships, pregnancy, HIV) and encouraged them to learn on their own (see Suppl Figure 2). At the end of the week, participants in both arms were provided with a single SMS-based quiz question on that domain's content. If the participant answered (correctly or not), they received a small amount of airtime (1USD). Intervention and contact messages were available in either Swahili or English, per the participant's preference. All SMS costs were reverse-billed to the study, so intervention and contact arm participants incurred no costs from their participation. Those randomized to Arm 3 (Control arm) received standard of care (no messages).

Assessments and Outcome

The primary outcome was assessed using an index developed by the research team of 10 contraception myths and misconceptions (Box 1). These were identified based on literature review and a series of focus group discussions with young people prior to the start of the RCT. In these sessions, young people used individual activities and group discussion centred around short vignettes of young couples thinking about starting contraception to describe local concerns around

For peer review only - http://bmjopen.bmj.com/site/about/guidelines.xhtml

contraception use.[31] At baseline and endline, RCT participants were asked to state how much they agreed or disagreed with a given statement based on a four-point Likert scale.

Box 1. Myths and misconceptions

Health - People who use contraceptives end up with health problems;

Body shape - Hormonal contraceptives are fattening;

Infertility – (1) After a woman uses contraceptive methods, it is difficult to get pregnant, and (2) use of a contraceptive injection can make a woman permanently infertile;

Harm - Contraceptives can harm a woman's womb;

Sex drive - Contraceptives reduce women's sexual urges;

Cancer - Contraceptives can cause cancer;

Malformations - Contraceptives can give you deformed babies; and

Social constructs – (1) Birth control should be a female concern; and (2) women who use family planning/birth-spacing may become promiscuous.

Sample Size

The sample size was calculated such that it provided 80% power to detect a 10% change in mean number of myths believed from baseline to endline, assuming that baseline level of belief was 55%, Type 1 error at 5% using two-sided Z test with continuity correction and unpooled variance and accounting for a dropout rate of up to 20%. The sample size accounted for Bonferroni correction due to 3-arm pairwise comparisons. Based on the aforementioned, a minimum number of participants to be sampled was 705, split evenly across intervention, contact, and control arms (1:1:1).

Randomization procedures

Participants were individually randomized to either intervention, contact or control group using a ratio of 1:1:1 as per computer-generated randomization schedule developed using Node.js and docker. All the study participants had equal probability of being assigned to either arm. Allocation took place after the participant had completed the baseline survey. ARMADILLO was an open-label trial;

For peer review only - http://bmjopen.bmj.com/site/about/guidelines.xhtml

however, neither the technological partner nor the research team had any control of arm assignments.

Data analysis

The 10 items of the primary outcome were dichotomized from the original Likert scale (Strongly agree, Agree, Disagree and Strongly disagree) as follows: 1) Agree and Strongly Agree [participant believed the myth – bad] were recoded as Agree and coded as 1; 2) Disagree and Strongly Disagree [participant rejected the myth – good] were recoded as Disagree and coded as 0. A participant score for the 10 questions was generated with a total maximum score of 10, corresponding to the number of myths/misconceptions that the participant believed. The average number of myths/misconceptions believed by participants in each arm was computed. There were no missing values for the 10 items across all arms. The study participants responded to all the 10 primary outcome questions in the assessment.

The baseline factors were described using proportions. To ensure that oversampling in certain arms had no effect on the randomization, we performed χ^2 tests on demographic characteristics to confirm that there were no baseline differences between arms. To assess attrition bias (a systematic error caused by unequal loss of participants from the trial between the baseline and the endline), we used Fisher's exact χ^2 tests for the socio-demographic variables to test whether participants lost to follow up differed across the trial arms (Suppl Table 1) as well as if they differed from those who responded as a function of study group (Suppl Table 2).

First, we present proportions of those who believed in the myths at baseline and endline for all arms and the percentage change in the myths believed between the two periods. To obtain the average number of myths believed per participant, we computed *(using the sum of the dichotomized 10-item response)* the number of myths believed for each participant at baseline and at endline. Then for each

For peer review only - http://bmjopen.bmj.com/site/about/guidelines.xhtml

participant, we computed the average number of myths believed (expressed as a percentage) at the baseline and at endline (by dividing the sum of myths believed by 10 and multiplying by 100). Next, for each participant, his or her absolute change in the average myths believed between the endline and baseline was computed (endline minus baseline).

Normality of the absolute changes in the myths believed was tested using the quantile-quantile plots. As the distribution of the absolute changes in the myths believed was normally distributed, Analysis of Variance (ANOVA) test of equality of group means was used to test the between group differences in the means of the absolute myths change. We estimated the difference-in-differences (DID) of the average number of myths believed by participants in a given arm to evaluate the effect of the ARMADILLO intervention to dispel myths and misconceptions about contraception. DID tells us whether the expected mean change in the number of myths and misconceptions believed from baseline to endline was different in the groups compared. DID is calculated by subtracting the average of the outcome in the control or contact arm from the average of the outcome in the intervention arm ($\hat{d}_1$), where the outcome is the change in percentage number of myths believed by each individual between the endline and baseline. DID was also used to assess changes in the average proportion of myths believed per participant between the control and the contact arm ($\hat{d}_2$).

For peer review only

$$\hat{d}_1 = \{[Mean(Y_i(Endline)) - Mean(Y_i(Baseline))]_{Intervention}\}\{[Mean(Y_i(Endline)) - Mean(Y_i(Baseline))]_j\}$$

$$\hat{d}_2 = \{[Mean(Y_i(Endline)) - Mean(Y_i(Baseline))]_{Contact}\} - \{[Mean(Y_i(Endline)) - Mean(Y_i(Baseline))]_{Control}\}$$

$Where\ \boldsymbol{i}\ refer\ to\ the\ ith\ individual\ in\ the\ trial\ arm;\ while\ \boldsymbol{j} = Contact\ or\ control$

For peer review only - http://bmjopen.bmj.com/site/about/guidelines.xhtml

All analyses were based on Complete Case (CC) dataset while analyses based on per protocol (PP) dataset were used for sensitivity analysis. Participants were included in the analysis provided that they had completed both baseline and endline surveys. In this case, the ITT analysis was equivalent to the CC.

Participants were included in the per protocol analysis provided that they had completed baseline and endline surveys, *and* that the intervention system could confirm that they had 1) received the ARMADILLO message domain associated with the primary outcome (Pregnancy Prevention); and 2) requested at least one message from this domain. Results with a Type I error of $p<0.05$ in two-sided tests were considered statistically significant. Where Bonferroni correction was applied for the pairwise comparisons of the three study arms, $p<0.017$ were considered statistically significant. Analyses were performed using Stata version 15, and all were conducted in accordance with a prespecified Statistical Analysis Plan.

Patients and public involvement statement

ARMADILLO's population of 'young people' were involved in the study from its initial, formative stage[28, 32], which included message content development. They and the broader community continued engagement through this trial through the ARMADILLO Community Advisory Board (CAB) consisting of representatives from the Ministries of Health, Education, and Social Services; youth-led organizations; area chiefs; health care workers providing SRH services to adolescents; and young people themselves. CAB members provided technical and field support throughout the data collection period. Additionally, young people identified from the study area were trained as data collectors and hired to enumerate young people in the area as well as recruit and implement baseline and endline surveys with study participants. Young data collectors' input also shaped recruitment hours and strategies. A

For peer review only - http://bmjopen.bmj.com/site/about/guidelines.xhtml

dissemination involving local and national stakeholders took place in July 2019 – selected young data collectors participated in the dissemination meeting and shared their feedback.

Ethical considerations

The study was approved by the ethics committees at the Kenyatta National Hospital (KNH ERC P550/09/2014) and the World Health Organization (Protocol WHO A65892). This trial is registered with the ISRCTN Registry (ISRCTN85156148).

Results

A total of 740 men and women aged 18-24 years were randomized into intervention, contact and control arms (Figure 1).

Figure 1 ARMADILLO Peru's Consolidated Standards of Reporting Trials (CONSORT) diagram

In the intervention period which lasted 7 weeks, 116 of the 740 (16%) study participants dropped out over the course of the seven-week intervention period (Intervention arm – 49 (19%); Contact arm – 42 (17%); Control arm – 25 (11%)). Among participants in the intervention arm, 206 (81%) completed both the baseline and the endline assessments (making them eligible for CC analysis) while 103 (40%) were eligible for per protocol (PP) analysis. Among the contact group, 207 (83%) received the push messages and were included in both the CC and PP analysis. In the control group, 211 (89.4%) completed both the baseline and the endline assessments. Baseline characteristics for the study sample are shown in Table 1. Across all arms, 53% of the participants were male, 48% had a secondary education or higher, 65% were from Ukunda sub-location, 91% lived with others, and 85% did not have children. There were no significant baseline differences between the intervention, contact and control groups.

For peer review only

Table 1: Baseline characteristics of the participants, by study arm (N = 740)

Characteristic	Intervention (I)	Contact (P)	Control (C)	Total
	N=255, n (%)	N=249, n (%)	N=236, n (%)	N=740, n (%)
**Age of the participant**				
18 – 19 years	62 (24.3)	55 (22.09)	53 (22.5)	170 (23.0)
20 – 24 years	193 (75.7)	194 (77.91)	183 (77.5)	570 (77.0)
**Sex**				
Male	134 (52.6)	133 (53.41)	126 (53.4)	393 (53.1)
Female	121 (47.5)	116 (46.59)	110 (46.6)	347 (46.9)
**Education level**				
Never gone to school	9 (3.5)	7 (2.81)	12 (5.1)	28 (3.8)
Primary school	92 (36.1)	97 (38.96)	80 (33.9)	269 (36.4)
Secondary school	117 (45.9)	118 (47.39)	119 (50.4)	354 (47.8)
Post-secondary education	37 (14.5)	27 (10.84)	25 (10.6)	89 (12.1)
**Sub-location**				
Ngombeni	34 (13.3)	38 (15.26)	43 (18.2)	115 (15.5)
Kitivo	9 (3.5)	9 (3.61)	9 (3.8)	27 (3.7)
Simkumbe	20 (7.8)	17 (6.83)	21 (8.9)	58 (7.8)
Mkoyo	8 (3.1)	8 (3.21)	8 (3.4)	24 (3.2)
Gombato	17 (6.7)	11 (4.42)	9 (3.8)	37 (5.0)
Ukunda	167 (65.5)	166 (66.67)	146 (61.9)	479 (64.7)
**Person currently living with**				
Living alone	24 (9.4)	21 (8.43)	21 (8.9)	66 (8.9)
Living with others	231 (90.6)	228 (91.57)	215 (91.1)	674 (91.1)
**Current relationship status**				
Single	128 (50.2)	121 (48.59)	118 (50.0)	367 (49.6)
Friends with benefits/ Dating/Cohabiting/ Engaged	109 (42.8)	104 (41.77)	92 (39.0)	305 (41.2)
Married	18 (7.1)	24 (9.64)	26 (11.0)	68 (9.2)
**Parity**				
None	224 (87.8)	211 (84.7)	191 (80.9)	626 (84.6)
1 child	24 (9.4)	28 (11.2)	35 (14.8)	87 (11.8)
2+ children	7 (2.8)	10 (4.0)	10 (4.2)	27 (3.7)
**First birth age**				
Never given birth	224 (87.8)	211 (84.7)	191 (80.9)	626 (84.6)
<=19 years (Adolescents)	18 (7.1)	22 (8.8)	23 (9.8)	63 (8.5)
>=20 years (Young women)	13 (5.1)	16 (6.4)	18 (7.6)	47 (6.4)

For peer review only - http://bmjopen.bmj.com/site/about/guidelines.xhtml

Concerning attrition bias, the analysis revealed that participants who dropped out in each arm were similar to each other (Suppl Table 1). However, there was a significant association between dropping out of the study and the number of children the participant had at the time of the study in the control and the contact groups' participants, Suppl. table1. The analysis assessing attrition bias also revealed that there was no significant difference in the socio-demographics between the participants who were lost to follow up and those who finished the seven weeks of the intervention and took the endline survey, Suppl. table 2.

The results of the CC analysis examining the myths and misconceptions believed are displayed in Table 2. The myths are ordered by those which were believed by the most number of participants, across groups, with the most salient myth at the top. The results show that at baseline, study participants in all arms believed around half of the myths related to contraception on average. At the end of the seven-week intervention period, the average number of myths and misconceptions believed per participant had significantly decreased for all the three groups ($p<0.0001$). The average absolute decrease in the myths believed was 11.1% among the intervention group (mean -11.1%; 95% CI [-17.1%,-5.2%]); 14.4% among the contact group (mean -14.4%; 95% CI [-20.5%,-8.4%]) and 11.3% among the control group (mean -11.3%; 95% CI [-17.4%,-5.2%]). From the normality test shown in Figure 2, the absolute change in all the three groups were normally distributed. The ANOVA test of equality-of-populations means showed that there was no significant difference in the group medians ($p=0.5181$).

Figure 2: Checking the normality of the absolute changes in the myths believed

For peer review only - http://bmjopen.bmj.com/site/about/guidelines.xhtml
For peer review only

Table 2: Intervention Effects for Dichotomous Outcomes – Complete Case Analysis

	Control (n=211)			Intervention (n=206)			Contact (n=207)		
**Myth**	**Baseline**	**Endline**	**Diff.**	**Baseline**	**Endline**	**Diff.**	**Baseline**	**Endline**	**Diff.**
**Population based analysis**									
Hormonal contraceptives are fattening	139 (65.9%)	117 (55.5%)	-10.4%	144 (69.9%)	135 (65.5%)	-4.4%	142 (68.6%)	127 (61.4%)	-7.2%
Contraceptives can harm a woman's womb	135 (64%)	114 (54%)	-10%	125 (60.7%)	106 (51.5%)	-9.2%	144 (69.6%)	112 (54.1%)	-15.5%
People who use contraceptives end up with health problems	133 (63%)	103 (48.8%)	-14.2%	120 (58.3%)	105 (51%)	-7.3%	135 (65.2%)	100 (48.3%)	-16.9%
Contraceptives can cause cancer	117 (55.5%)	88 (41.7%)	-13.8%	112 (54.4%)	90 (43.7%)	-10.7%	123 (59.4%)	86 (41.6%)	-17.8%
Use of a contraceptive injection can make a woman permanently infertile	108 (51.2%)	82 (38.9%)	-12.3%	102 (49.5%)	73 (35.4%)	-14.1%	123 (59.4%)	77 (37.2%)	-22.2%
Contraceptives reduce women's sexual urges	101 (47.9%)	65 (30.8%)	-17.1%	83 (40.3%)	57 (27.7%)	-12.6%	107 (51.7%)	76 (36.7%)	-15%
Contraceptives can give you deformed babies	98 (46.5%)	69 (32.7%)	-13.8%	92 (44.7%)	53 (25.7%)	-19%	106 (51.2%)	71 (34.3%)	-16.9%
After a woman uses contraceptive methods, it is difficult to get pregnant	92 (43.6%)	81 (38.4%)	-5.2%	101 (49%)	69 (33.5%)	-15.5%	102 (49.3%)	80 (38.7%)	-10.6%
Birth control should be a female concern	59 (28%)	41 (19.4%)	-8.6%	46 (22.3%)	32 (15.5%)	-6.8%	59 (28.5%)	35 (16.9%)	-11.6%
Women who use family planning / birth-spacing may become promiscuous	109 (51.7%)	92 (43.6%)	-8.1%	108 (52.4%)	84 (40.8%)	-11.6%	111 (53.6%)	89 (43%)	-10.6%
**Subject specific analysis**									
Average # myths believed, per participant (Std. Error)	***5.17***	***4.04***		***5.01***	***3.90***		***5.57***	***4.12***	
Average absolute change in myths believed	***-1.13***			***-1.11***			***-1.44***		
Conf. interval of the diff.	***[-1.59, -0.67]***			***[-1.54, -0.68]***			***[-1.91, -0.98]***		
Percentage absolute change in myths believed	**-21.9%**			**-22.2%**			**-25.9%**		
Test of diff. in the mean of the absolute change in myths believed	Control (n=211)			Intervention (n=206)			Contact (n=207)		
ANOVA test F statistics	***0.66***								
P-value	***0.5181***								

n* – Those who believed the myth (had wrong answer);

For peer review only

As presented in the difference-in-difference analysis in Table 3, there was no statistically significant differences between the baseline to endline decrease observed across the 3-arms (p>0.017-Bonferroni corrected significance level).

Table 3: Difference in difference analysis

Outcome	Percentage point differences (95%CI)	p-value
Contraception myths and misconceptions index score (endline – baseline assessment)		
Arm 1: Intervention (MeanΔ, 95%CI)	-11.1% (-17.1%,-5.2%)	<0.001
Arm 2: Contact (MeanΔ, 95%CI)	-14.4% (-20.5%,-8.4%)	<0.001
Arm 3: Control (MeanΔ, 95%CI)	-11.3% (-17.4%,-5.2%)	<0.001
Mean (Δ Intervention) - Mean (Δ Control)	0.2% (-8.3%, 8.7%)	0.961
Mean (Δ Contact) - Mean (Δ Control)	-3.1% (-11.7%, 5.4%)	0.475
Mean (Δ Intervention) - Mean (Δ Contact)	3.3% (-5.1%, 11.8%)	0.440

Notes: Δ refers to the subject-specific change in the outcome from baseline to endline. 95%CI refers to the 95% confidence interval. A generalized linear model (GLM) using a normal distribution and identity link was used to compare scores. Effective sample size used for the analysis was 206 participants for the intervention group; 207 participants for the contact group and 211 participants for the control group.

Sensitivity analysis including only participants who met PP inclusion criteria did not alter the findings from CC analysis. As the results of the CC analysis, and PP analysis did not differ with respect to statistical significance within groups or between groups, only those for the CC analysis are reported (PP analysis is attached as supplementary materials – Suppl. table 3).

Finally, Table 4 shows estimates of a possible source of contamination between study arms. Of the 23% of the participants in the intervention group who shared the messages with others, 13%, 5%, and 4% shared the messages with friends, partners, and multiple contacts, respectively. Among the 27% contact group participants who shared the messages with others, 15%, 8%, 1% and 3% shared the messages with friends, partners, siblings and multiple contacts, respectively.

For peer review only

Table 4: Study contamination

		Shared the messages	Shared the messages with: Siblings	Friends	Partner	Parents	multiple contacts
Intervention (N=206)	n	47	0	26	10	2	9
	%	22.8%	0.0%	12.6%	4.9%	1.0%	4.4%
Contact (N=207)	n	55	2	30	16	0	7
	%	26.6%	1.0%	14.5%	7.7%	0.0%	3.4%
Total (N=413)	n	102	2	56	26	2	16
	%	24.7%	0.5%	13.6%	6.3%	0.5%	3.9%

Discussion

Our findings suggest that provision of sexual and reproductive health content via SMS is potentially useful in dealing with contraception-related myths and misconceptions among youth. Across arms, the study demonstrated between a 11% and 14% reduction in the average number of myths/misconception statements believed over the study period. However, we did not observe a significant difference in the magnitude of reduction between the arms. Therefore, despite the significant decrease in myths-believed that we observed between baseline and endline, we are unable to conclusively state that the ARMADILLO intervention was better than SMS 'contact' or no intervention at all.

One possible reason for not seeing a significant effect of the ARMADILLO intervention versus no intervention is that correcting false information is difficult. Studies aimed at correcting misinformation about vaccines, for example, have shown that even when attempts to correct invalid information do not entrench the original misinformation, they can frequently fail because people cannot successfully update their memories, and still fall back on information they know is not correct. [33-36] A seven-week digital health intervention, dedicated to sexual and reproductive health broadly, may not have been enough to dispel deeply-entrenched concerns about contraception. Myths and misconceptions

For peer review only

around contraception are also particularly tricky given that misconception about contraception generally (e.g. that contraception use can lead to infertility) may be partially rooted in individual experiences of real side effects (for example, the possible delay of a return to fertility following use of injectable contraception).[12]

Alternatively, we may have seen no difference between the arms because the intervention was truly not better than SMS-prompted self-learning and/or no intervention at all. Several other RCTs that have attempted to tie adolescent-targeted digital health interventions to sexual and reproductive health (SRH) knowledge, acceptability, or behavioural outcomes have resulted in similarly inconclusive findings, [26, 37-40] indicating that digital interventions *on their own* may not be enough to encourage behaviour change. However, while the above reasons would explain the lack of difference between arms, they do not explain why participants in all arms believed significantly fewer myths at endline than they did at baseline.

Here, contamination between arms may be to blame. Randomized controlled trials of digital health client communication interventions are difficult, especially when the intervention content can be easily shared between neighbours and across communities. To the best of our knowledge, there were no other health campaigns or interventions aimed at dispelling the myths and misconceptions in the area during the study period. However, about a quarter of the intervention and contact arm participants reported sharing information with study participants and other members of the community. Any effect of participants sharing information was amplified by our participants being randomized at the individual level. This resulted in participants in different arms living in close proximity, often in neighbouring households. ARMADILLO intervention arm participants may therefore have received the messages and shared them with their friends/neighbours, some of whom were ARMADILLO contact and control arm participants.

For peer review only - http://bmjopen.bmj.com/site/about/guidelines.xhtml

Addressing/dispelling myths and misconceptions among youth is particularly important for future contraception use. By nature of originating in social networks as well as their likelihood to "stick" indefinitely, myths and misconceptions among youth should be dispelled early to prevent their becoming engrained. [6, 41] Indeed, our study found that two of the three most commonly-believed myths and misconceptions among youth age 18-24 years (people who use contraceptives end up with health problems, and contraceptives can harm a woman's womb) were also the top myths for youth and adult women aged 15-49 years in Kenya, Nigeria and Senegal. [9]

Other studies have reported successfully addressing myths and misconceptions with dedicated community and communication interventions, [42] involving a variety of opinion leaders and channels. [9, 42] Following a four year intervention using radio, religious leaders, and community health volunteers, for example, Kenya's Tupange study reported a 15% decrease in the number of women who believed myths and misconceptions statements between baseline and endline. [42]

Digital interventions are an additional channel which can be included in this mix. There is a wealth of opportunity to engage with young people en masse not only through SMS and voice channels, but also by widely-used messaging and social media platforms like WhatsApp, Facebook, and Facebook Messenger, the latter of which don't have the bulk telecom-related costs of SMS and voice interventions. The popularity of the ARMADILLO interventions among its users – one silver lining of the contamination between arms - indicate that such interventions can be considered as one tool *in a multi-pronged approach* targeting young people with correct SRH information. [25, 43-45] However, evidence continues to be needed on adolescent-targeted client communication interventions in general.

This study is not without limitations. The lack of differences between the intervention and the other arms could very well be due to our adoption of individually-randomized rather than cluster-

For peer review only - http://bmjopen.bmj.com/site/about/guidelines.xhtml

randomized study design. A cluster design was considered; however, the accessibility and make-up of the study area did not allow for homogenous clusters to be created (Ukunda, for example, is unique in Kwale County for its population density). While unfortunate for the results of the trial, it demonstrates that the messages were as popular as the research team hoped and provides some positive insight as to the dissemination which might take place if a similar intervention is implemented outside of a research setting. The study is strengthened by its choice of primary outcome: we intentionally avoided powering the study around SRH behavioural outcomes, building on learnings from previous studies that light-touch digital interventions alone may not be enough to see behaviour change in such a complex area of health.

In conclusion, creative and consistent interventions are needed to address deeply-rooted myths and misconceptions young people have about contraception and mitigate one important driver of anxiety around contraception use in Kenya. These can include digital interventions. However, while the ARMADILLO study saw some promising results, we cannot conclusively say that digital interventions *alone* are sufficient to affect change in SRH-related outcomes. Additional research (using alternative designs) will be required to identify the specific value of digital targeted client communication programs in addressing young people's contraception myths/misconceptions and improving sexual and reproductive health knowledge overall.

Acknowledgments

The authors thank the youth in Kwale who participated in the study, the research assistants who collected data, and Winnie Wangari who coordinated and supervised data collection. The authors are grateful for feedback given by Carol Mukira and Nelly Ibeere.

For peer review only

Funding Statement

This work was funded by the UNDP/UNFPA/UNICEF/WHO/World Bank Special Programme of Research, Development and Research Training in Human Reproduction (HRP).

Competing interests

None declared.

Data Availability Statement

Deidentified data related to this article can be obtained by request to the corresponding author.

Author Contributorship

P.G. and L.G. contributed equally to this paper (in study design, interpretation of data and writing of the manuscript). M.J, M.T, N.H, M.T and L.S. contributed to the design of the ARMADILLO study. M.J. contributed to data collection; M.W. and M.T. contributed to the analysis; P.G, L.G, M.J, M.T, N.H, H.S, T.T, A.A, M.T and L.S contributed to interpretation of data. P.G and L.G wrote the first draft of the paper, and all authors contributed to its revision; all authors approve of the submitted article. The manuscript represents the views of the named authors only.

References

1. MacQuarrie KLD. Unmet Need for Family Planning among Young Women: Levels and Trends. Rockville, Maryland, USA: ICF International; 2014.
2. Kenya National Bureau of Statistics. Kenya Demographic and Health Survey 2014. Rockville, Maryland: The DHS Program, ICF International; 2015.
3. Kenya National Bureau of Statistics (KNBS). Kenya Demographic and Health Survey 2008-09. Calverton, Maryland: KNBS and ICF Macro; 2010.
4. Chandra-Mouli VMM, D.R. ; Phillips, S.J.; Williamson, N.E.; Hainsworth, G. . Contraception for adolescents in low and middle income countries: needs, barriers, and access. Reproductive Health. 2014;11(1).
5. Denno DMH, Andrea J; Chandra-Mouli, Venkatraman. Effective Strategies to Provide Adolescent Sexual and Reproductive Health Services and to Increase Demand and Community Support. Journal of Adolescent Health. 2015;56(1):S22-S41.
6. Speizer IS, Calhoun LM, Guilkey DK. Reaching Urban Female Adolescents at Key Points of Sexual and Reproductive Health Transitions: Evidence from a Longitudinal Study from Kenya. African journal of reproductive health. 2018;22(1):47-59.
7. Parker JJ, Veldhuis CB, Hughes TL, Haider S. Barriers to contraceptive use among adolescents in two semi-rural Nicaraguan communities. International journal of adolescent medicine and health. 2019;32(5).
8. M AAAJO. Myths, misinformation, and communication about family planning and contraceptive use in Nigeria. Open Access Journal of Contraception. 2011;2:95-105.
9. Gueye A, Speizer IS, Corroon M, Okigbo CC. Belief in Family Planning Myths at the Individual and Community Levels and Modern Contraceptive Use in Urban Africa. International perspectives on sexual and reproductive health. 2015;41(4):191-9.
10. Paz Soldan VA. How family planning ideas are spread within social groups in rural Malawi. Studies in family planning. 2004;35(4):275-90.

For peer review only

11. Yee L, Simon M. The role of the social network in contraceptive decision-making among young, African American and Latina women. The Journal of adolescent health : official publication of the Society for Adolescent Medicine. 2010;47(4):374-80.
12. PATH. Countering myths and misperceptions about contraceptives. Seattle, United States: PATH; 2015.
13. Hightow-Weidman LB, Muessig KE, Bauermeister J, Zhang C, LeGrand S. Youth, Technology, and HIV: Recent Advances and Future Directions. Curr HIV/AIDS Rep. 2015;12(4):500-15.
14. World Bank. The Little Data Book on Information and Communication Technology 2014. Washington DC: World Bank; 2014.
15. L'Engle KL, Mangone ER, Parcesepe AM, Agarwal S, Ippoliti NB. Mobile Phone Interventions for Adolescent Sexual and Reproductive Health: A Systematic Review. Pediatrics. 2016;138(3).
16. Ippoliti NB, L'Engle K. Meet us on the phone: mobile phone programs for adolescent sexual and reproductive health in low-to-middle income countries. Reprod Health. 2017;14(1):11.
17. Demombynes GT, Aaron. Kenya's Mobile Revolution and the Promise of Mobile Savings. World Bank Group; 2012.
18. Kalichman SC, Cherry C, Kalichman MO, Eaton LA, Kohler JJ, Montero C, et al. Mobile Health Intervention to Reduce HIV Transmission: A Randomized Trial of Behaviorally Enhanced HIV Treatment as Prevention (B-TasP). J Acquir Immune Defic Syndr. 2018;78(1):34-42.
19. Widman L, Nesi J, Kamke K, Choukas-Bradley S, Stewart JL. Technology-Based Interventions to Reduce Sexually Transmitted Infections and Unintended Pregnancy Among Youth. The Journal of adolescent health : official publication of the Society for Adolescent Medicine. 2018;62(6):651-60.
20. Smith C, Ngo TD, Gold J, Edwards P, Vannak U, Sokhey L, et al. Effect of a mobile phone-based intervention on post-abortion contraception: a randomized controlled trial in Cambodia. Bull World Health Organ. 2015;93(12):842-50A.
21. Anderberg P, Barnestein-Fonseca P, Guzman-Parra J, Garolera M, Quintana M, Mayoral-Cleries F, et al. The Effects of the Digital Platform Support Monitoring and Reminder Technology for Mild Dementia (SMART4MD) for People With Mild Cognitive Impairment and Their Informal Carers: Protocol for a Pilot Randomized Controlled Trial. JMIR research protocols. 2019;8(6):e13711.
22. Vahdat HL, L'Engle KL, Plourde KF, Magaria L, Olawo A. There are some questions you may not ask in a clinic: Providing contraception information to young people in Kenya using SMS. International Journal of Gynecology & Obstetrics. 2013;123(S1):e2-e6.
23. Rokicki S, Fink G. Assessing the reach and effectiveness of mHealth: evidence from a reproductive health program for adolescent girls in Ghana. BMC Public Health. 2017;17(1):969.
24. Castaño PM, Bynum JY, Andrés R, Lara M, Westhoff C. Effect of daily text messages on oral contraceptive continuation: a randomized controlled trial. Obstetrics and gynecology. 2012;119(1):14-20.
25. Garofalo R, Kuhns LM, Hotton A, Johnson A, Muldoon A, Rice D. A Randomized Controlled Trial of Personalized Text Message Reminders to Promote Medication Adherence Among HIV-Positive Adolescents and Young Adults. AIDS and behavior. 2016;20(5):1049-59.
26. Lim MSC, Hocking JS, Aitken CK, Fairley CK, Jordan L, Lewis JA, et al. Impact of text and email messaging on the sexual health of young people: a randomised controlled trial. J Epidemiol Community Health. 2012;66(1):69-74.
27. World Health Organization. WHO guideline: recommendations on digital interventions for health system strengthening Web Supplement 2: Summary of findings and GRADE tables. Geneva: WHO; 2019 2019. Report No.: WHO/RHR/19.7.
28. Gonsalves LLE, KL; Tamrat, T; Plourde, K.F.; Mangone, E.R.; Agarwal, S.; Say, L.; and Hindin, M.J. Adolescent/Youth Reproductive Mobile Access and Delivery Initiative for Love and Life Outcomes (ARMADILLO) Study: formative protocol for mHealth platform development and piloting. Reproductive Health. 2015;12(67).
29. Gonsalves LH, MJ; Bayer, A; Carcamo, CP; Gichangi, P; Habib, N; Mwaisaka, J; Say, L. Protocol of an open, three-arm, individually randomized trial assessing the effect of delivering sexual and reproductive health information to young people (aged 13–24) in Kenya and Peru via mobile phones: adolescent/youth reproductive mobile access and delivery initiative for love and life outcomes (ARMADILLO) study stage 2. Reproductive Health. 2018;15(126).

For peer review only - http://bmjopen.bmj.com/site/about/guidelines.xhtml

30. World Health Organization. Classification of digital health interventions v1.0. Geneva, Switzerland: World Health Organization; 2018.
31. Mwaisaka J, Gonsalves L, Thiongo M, Waithaka M, Sidha H, Agwanda A, et al. Exploring contraception myths and misconceptions among young men and women in Kwale County, Kenya. BMC Public Health. 2020;20(1):1694.
32. Mwaisaka JG, Lianne; Say, Lale; Gichangi, Peter. “What’s that on your phone?” The aftermath of parents finding sexual and reproductive health messages on their children’s phone in coastal Kenya. Journal of Health and Social Sciences. 2018;3(2):147-56.
33. Chan M-pS, Jones CR, Hall Jamieson K, Albarracín D. Debunking: A Meta-Analysis of the Psychological Efficacy of Messages Countering Misinformation. Psychological Science. 2017;28(11):1531-46.
34. Lewandowsky S, Ecker UKH, Seifert CM, Schwarz N, Cook J. Misinformation and Its Correction: Continued Influence and Successful Debiasing. Psychological Science in the Public Interest. 2012;13(3):106-31.
35. Pluviano S, Watt C, Della Sala S. Misinformation lingers in memory: Failure of three pro-vaccination strategies. PloS one. 2017;12(7):e0181640.
36. Seifert CM. The continued influence of misinformation in memory: What makes a correction effective? Psychology of Learning and Motivation. 41: Academic Press; 2002. p. 265-92.
37. Free C, McCarthy O, French RS, Wellings K, Michie S, Roberts I, et al. Can text messages increase safer sex behaviours in young people? Intervention development and pilot randomised controlled trial. Health technology assessment (Winchester, England). 2016;20(57):1-82.
38. Perry RC, Kayekjian KC, Braun RA, Cantu M, Sheoran B, Chung PJ. Adolescents' perspectives on the use of a text messaging service for preventive sexual health promotion. The Journal of adolescent health : official publication of the Society for Adolescent Medicine. 2012;51(3):220-5.
39. McCarthy O, Ahamed I, Kulaeva F, Tokhirov R, Saibov S, Vandewiele M, et al. A randomized controlled trial of an intervention delivered by mobile phone app instant messaging to increase the acceptability of effective contraception among young people in Tajikistan. Reproductive Health. 2018;15(1):28.
40. McCarthy OL, Aliaga C, Torrico Palacios ME, López Gallardo J, Huaynoca S, Leurent B, et al. An Intervention Delivered by Mobile Phone Instant Messaging to Increase Acceptability and Use of Effective Contraception Among Young Women in Bolivia: Randomized Controlled Trial. Journal of medical Internet research. 2020;22(6):e14073-e.
41. da Silva-Filho AL, Lira J, Rocha ALL, Carneiro MM. Barriers and myths that limit the use of intrauterine contraception in nulliparous women: a survey of Brazilian gynaecologists. Postgraduate medical journal. 2017;93(1101):376-81.
42. Muthamia M, Owino K, Nyachae P, Kilonzo M, Kamau M, Otai J, et al. The Tupange Project in Kenya: A Multifaceted Approach to Increasing Use of Long-Acting Reversible Contraceptives. Glob Health Sci Pract. 2016;4 Suppl 2(Suppl 2):S44-S59.
43. L'Engle K, Plourde KF, Zan T. Evidence-based adaptation and scale-up of a mobile phone health information service. mHealth. 2017;3:11.
44. Lee S, Begley CE, Morgan R, Chan W, Kim S-Y. Addition of mHealth (mobile health) for family planning support in Kenya: disparities in access to mobile phones and associations with contraceptive knowledge and use. International Health. 2019;11(6):463-71.
45. McCarthy OL, Zghayyer H, Stavridis A, Adada S, Ahamed I, Leurent B, et al. A randomized controlled trial of an intervention delivered by mobile phone text message to increase the acceptability of effective contraception among young women in Palestine. Trials. 2019;20(1):228.

For peer review only

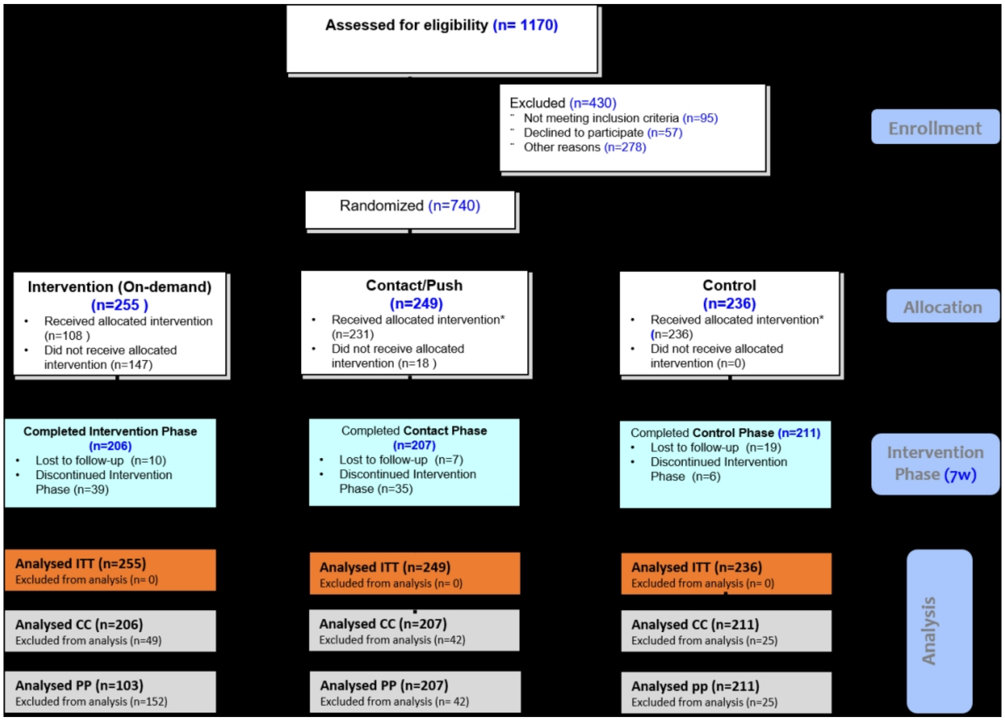

Figure 1. Consolidated Standards of Reporting Trials (CONSORT) diagram

233x166mm (150 x 150 DPI)

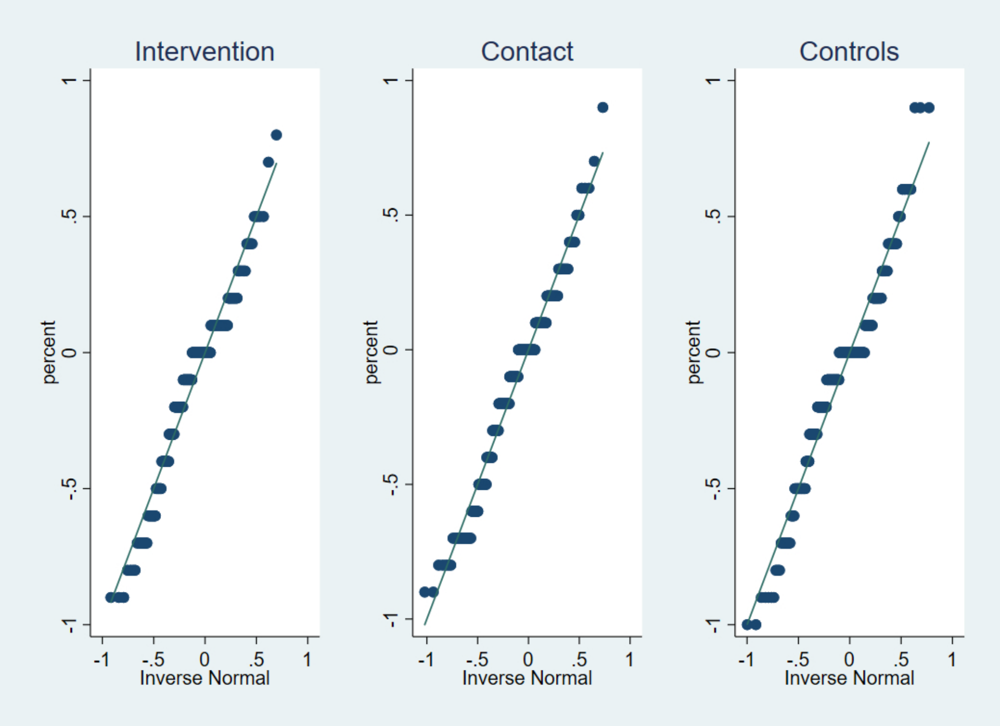

Figure 2: Checking the normality of the absolute changes in the myths believed

274x199mm (96 x 96 DPI)

Supplementary materials

Suppl. table 1: Assessing attrition bias across the study arms

Characteristic	Control (C)	Intervention (I)	Contact (P)	Assessing the baseline differences for drop-outs		
	N=25, n (%)	N=49, n (%)	N=42, n (%)	C vs I p-value	I vs P p-value	C vs P p-value
**Age of the participant**						
18 – 19	5 (20.0)	14 (28.57)	13 (30.95)	0.58	0.82	0.24
20 – 24	20 (80.0)	35 (71.43)	29 (69.05)			
**Sex**						
Male	12 (48.0)	24 (48.98)	21 (50.0)	1.00	1.00	1.00
Female	13 (52.0)	25 (51.02)	21 (50.0)			
**Highest level of school attended**						
Never gone to school	2 (8.0)	1 (2.04)	0 (0)	0.41	0.68	0.42
Primary school	8 (32.0)	21 (42.86)	16 (38.1)			
Secondary school	12 (48.0)	24 (48.98)	21 (50)			
Post-secondary education	3 (12.0)	3 (6.12)	5 (11.9)			
**Sub-location**						
Ngombeni	2 (8.0)	9 (18.37)	10 (23.81)	0.70	0.69	0.31
Kitivo	1 (4.0)	0 (0)	0 (0)			
Simkumbe	1 (4.0)	2 (4.08)	3 (7.14)			
Mkoyo	1 (4.0)	3 (6.12)	2 (4.76)			
Gombato	1 (4.0)	2 (4.08)	4 (9.52)			
Ukunda	19 (76.0)	33 (67.35)	23 (54.76)			
**Person currently living with**						
Living alone	1 (4.0)	4 (8.16)	3 (7.14)	0.66	1.00	1.00
Living with others	24 (96.0)	45 (91.84)	39 (92.86)			

For peer review only

**Current relationship status**						
Single	12 (48.0)	28 (57.14)	23 (54.76)	0.36	0.69	0.83
Friends with benefits / Dating / Cohabiting / Engaged	9 (36.0)	18 (36.73)	14 (33.33)			
Married	4 (16.0)	3 (6.12)	5 (11.9)			
**Number of children the participant have**						
None	16 (64.0)	41 (83.67)	37 (88.1)	0.14	0.87	**0.03****
1 child	7 (28.0)	7 (14.29)	5 (11.9)			
2+ children	2 (8.0)	1 (2.04)	0 (0)			
**First birth age**						
Never given birth	16 (64.0)	41 (83.67)	37 (88.1)	0.42	0.90	0.15
<=19 years (Adolescents)	5 (20.0)	5 (10.2)	3 (7.14)			
>=20 years (Young women)	2 (8.0)	3 (6.12)	2 (4.76)			
Missing	2 (8.0)	0 (0)	0 (0)			

Different rates of loss to follow up in the exposure groups, or losses of different types of participants, whether at similar or different frequencies may change the characteristics of the groups, irrespective of the exposure or intervention. This analysis was done to confirm if there were systematic differences among the people who were lost to follow up across the different study arms.

For peer review only - http://bmjopen.bmj.com/site/about/guidelines.xhtml

Suppl. table 2: Assessing attrition bias (drop-outs vs those who took the endline assessment)

Characteristic	Intervention (I)			Contact (P)			Control (C)		
	Drop-outs	Endline participants		Drop-outs	Endline participants		Drop-outs	Endline participants	
	N=49, n (%)	N=206, n (%)	Fisher's test p-value	N=42, n (%)	N=207, n (%)	Fisher's test p-value	N=25, n (%)	N=211, n (%)	Fisher's test p-value
**Age of the participant**									
18 – 19 years (Adolescents)	14 (28.57)	48 (23.3)	0.46	13 (30.95)	42 (20.3)	0.15	5 (20.0)	48 (22.8)	0.49
20 – 24 years (Young women)	35 (71.43)	158 (76.7)		29 (69.05)	165 (79.7)		20 (80.0)	163 (77.2)	
**Sex**									
Male	24 (48.98)	110 (53.4)	0.63	21 (50.0)	112 (54.1)	0.74	12 (48.0)	114 (54.0)	0.67
Female	25 (51.02)	96 (46.6)		21 (50.0)	95 (45.90		13 (52.0)	97 (46.0)	
**Highest level of school attended**									
Never gone to school	1 (2.04)	8 (3.9)	0.23	0 (0)	7 (3.4)	0.83	2 (8.0)	10 (4.7)	0.85
Primary school	21 (42.86)	71 (34.5)		16 (38.1)	81 (39.1)		8 (32.0)	72 (34.1)	
Secondary school	24 (48.98)	93 (45.2)		21 (50)	97 (46.9)		12 (48.0)	107 (50.7)	
Post-secondary education	3 (6.12)	34 (16.5)		5 (11.9)	22 (10.6)		3 (12.0)	22 (10.4)	
**Sublocation**									
Ngombeni	9 (18.37)	25 (12.1)	0.26	10 (23.81)	28 (13.5)	0.1	2 (8.0)	41 (19.4)	0.55
Kitivo	0 (0)	9 (4.4)		0 (0)	9 (4.4)		1 (4.0)	8 (3.8)	
Simkumbe	2 (4.08)	18 (8.7)		3 (7.14)	14 (6.8)		1 (4.0)	20 (9.5)	
Mkoyo	3 (6.12)	5 (2.4)		2 (4.76)	6 (2.9)		1 (4.0)	7 (3.3)	
Gombato	2 (4.08)	15 (7.3)		4 (9.52)	7 (3.4)		1 (4.0)	8 (3.8)	
Ukunda	33 (67.35)	134 (65.1)		23 (54.76)	143 (69.1)		19 (76.0)	127 (60.2)	
**Person currently living with**									
Living alone	4 (8.16)	20 (9.7)	1	3 (7.14)	18 (8.7)	1	1 (4.0)	20 (9.5)	0.71
Living with others	45 (91.84)	186 (90.3)		39 (92.86)	189 (91.3)		24 (96.0)	191 (90.5)	
Missing									
**Current relationship status**									
Single	28 (57.14)	100 (48.5)	0.58	23 (54.76)	98 (47.3)	0.41	12 (48.0)	106 (50.2)	0.68

For peer review only - http://bmjopen.bmj.com/site/about/guidelines.xhtml

Friends with benefits / Dating / Cohabiting / Engaged	18 (36.73)	15 (7.3)		14 (33.33)	19 (9.2)		9 (36.0)	22 (10.4)	
Married	3 (6.12)	91 (44.20		5 (11.9)	90 (43.5)		4 (16.0)	83 (39.3)	
**Number of children the participant have**									
None	41 (83.7)	183 (88.8)	0.4	37 (88.1)	174 (84.1)	0.46	16 (64.0)	175 (82.9)	0.06
1 child	7 (14.29)	17 (8.3)		5 (11.9)	23 (11.1)		7 (28.0)	28 (13.3)	
2+ children	1 (2.04)	6 (2.9)		0 (0)	10 (4.8)		2 (8.0)	8 (3.8)	
**First birth age**									
Never given birth	41 (83.67)	183 (88.8)	0.5	37 (88.1)	174 (84.1)	0.94	16 (64.0)	175 (82.9)	0.08
<=19 years (Adolescents)	5 (10.2)	13 (6.3)		3 (7.14)	19 (9.2)		5 (20.0)	18 (8.5)	
>=20 years (Young women)	3 (6.12)	10 (4.9)		2 (4.76)	14 (6.8)		2 (8.0)	16 (7.6)	
Missing	0 (0)	0		0 (0)	0		2 (8.0)	2 (1.0)	

Systematic difference between people who leave the study and those who continue can introduce bias in the study results, this is attrition bias. Suppl. table 2 shows the assessment of attrition bias which was achieved using fishers chi-square tests for categorical variables. This assessment was done to test whether participants lost to follow up differed from those who responded as a function of study group.

For peer review only

For peer review only - http://bmjopen.bmj.com/site/about/guidelines.xhtml

Suppl. table 3: Per Protocol analysis

Myth	Intervention (I) (n=103)			Contact (P) (n=207)			Control (C) (n=211)		
	Baseline	Endline	Diff.	Baseline	Endline	Diff.	Baseline	Endline	Diff.
**Population based analysis**									
Hormonal contraceptives are fattening	72 (69.9%)	71 (68.9%)	-1.00%	142 (68.6%)	127 (61.4%)	-7.20%	139 (65.9%)	117 (55.5%)	-10.40%
Contraceptives can harm a woman's womb	62 (60.2%)	49 (47.6%)	-12.60%	144 (69.6%)	112 (54.1%)	-15.50%	135 (64%)	114 (54%)	-10%
People who use contraceptives end up with health problems	60 (58.3%)	52 (50.5%)	-7.80%	135 (65.2%)	100 (48.3%)	-16.90%	133 (63%)	103 (48.8%)	-14.20%
Contraceptives can cause cancer	59 (57.3%)	47 (45.6%)	-11.70%	123 (59.4%)	86 (41.6%)	-17.80%	117 (55.5%)	88 (41.7%)	-13.80%
Use of a contraceptive injection can make a woman permanently infertile	47 (45.6%)	35 (34%)	-11.60%	123 (59.4%)	77 (37.2%)	-22.20%	108 (51.2%)	82 (38.9%)	-12.30%
Contraceptives reduce women's sexual urges	36 (35%)	28 (27.2%)	-7.80%	107 (51.7%)	76 (36.7%)	-15%	101 (47.9%)	65 (30.8%)	-17.10%
Contraceptives can give you deformed babies	44 (42.7%)	26 (25.2%)	-17.50%	106 (51.2%)	71 (34.3%)	-16.90%	98 (46.5%)	69 (32.7%)	-13.80%
After a woman uses contraceptive methods, it is difficult to get pregnant	47 (45.6%)	32 (31.1%)	-14.50%	102 (49.3%)	80 (38.7%)	-10.60%	92 (43.6%)	81 (38.4%)	-5.20%
Birth control should be a female concern	20 (19.4%)	16 (15.5%)	-3.90%	59 (28.5%)	35 (16.9%)	-11.60%	59 (28%)	41 (19.4%)	-8.60%
Women who use family planning / birth-spacing may become promiscuous	52 (50.5%)	42 (40.8%)	-9.70%	111 (53.6%)	89 (43%)	-10.60%	109 (51.7%)	92 (43.6%)	-8.10%
**Subject specific analysis**									
Average # myths believed, per participant (Std. Error)	***5.17***	***4.04***		***5.01***	***3.90***		***5.57***	***4.12***	
Average absolute change in myths believed	***-1.13***			***-0.98***			***-1.44***		
Conf. interval of the diff.	***[-1.59, -0.67]***			***[-1.57, -0.39]***			***[-1.91, -0.98]***		

For peer review only - http://bmjopen.bmj.com/site/about/guidelines.xhtml
For peer review only

Percentage absolute change in myths believed	**-21.9%**	**-20.2%**	**-25.9%**
Test of diff. in the mean of the absolute change in myths believed	Control (n=211)	Intervention (n=103)	Contact (n=207)
ANOVA test F statistics	***0.81***		
P-value	***0.4458***		

For peer review only

Suppl. Figure 1. The interventions each arm received

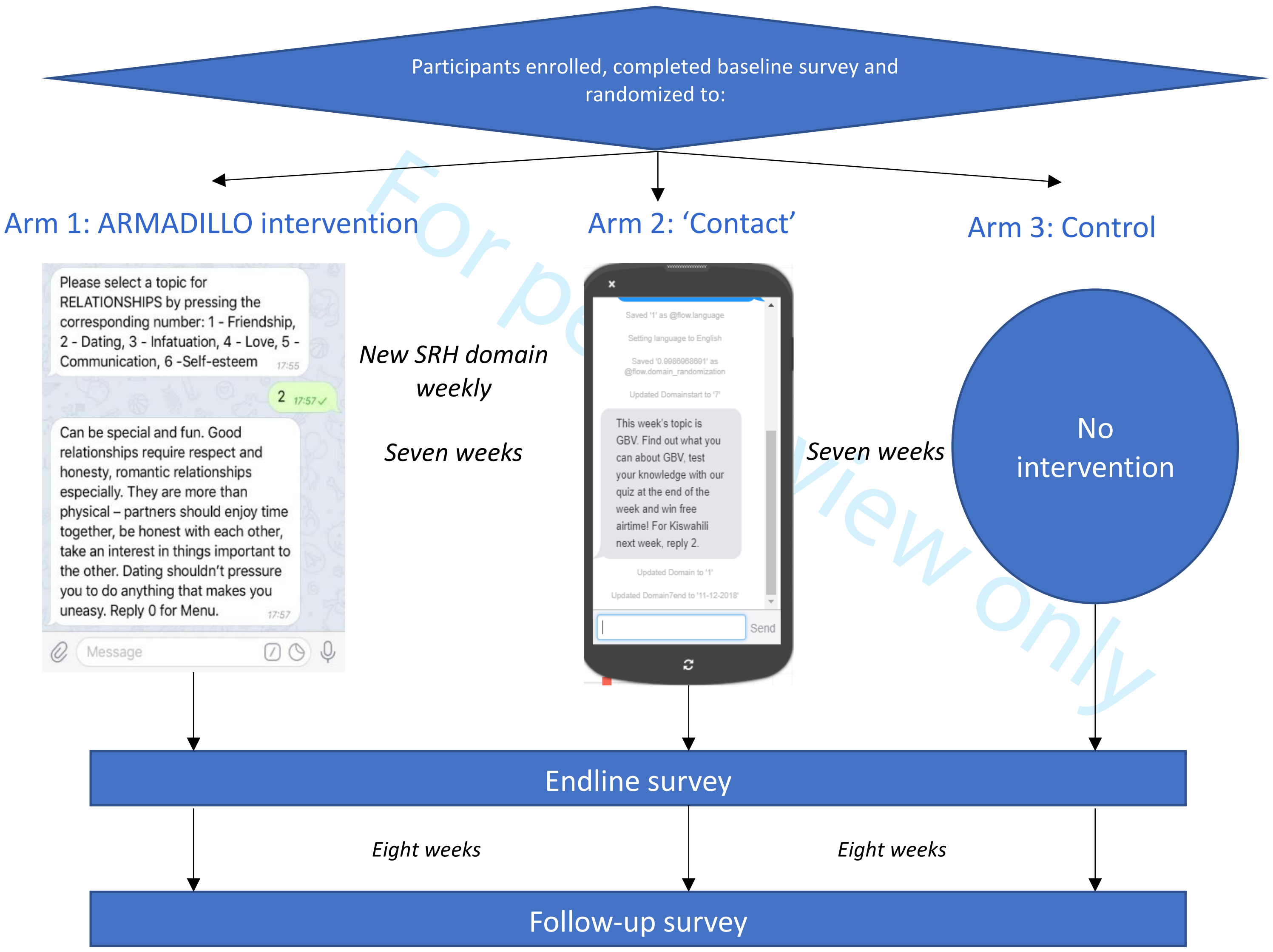

For peer review only

Suppl. Figure 2. The message domains which the intervention arms participants could request for more information and contacts arms participants studied on their own

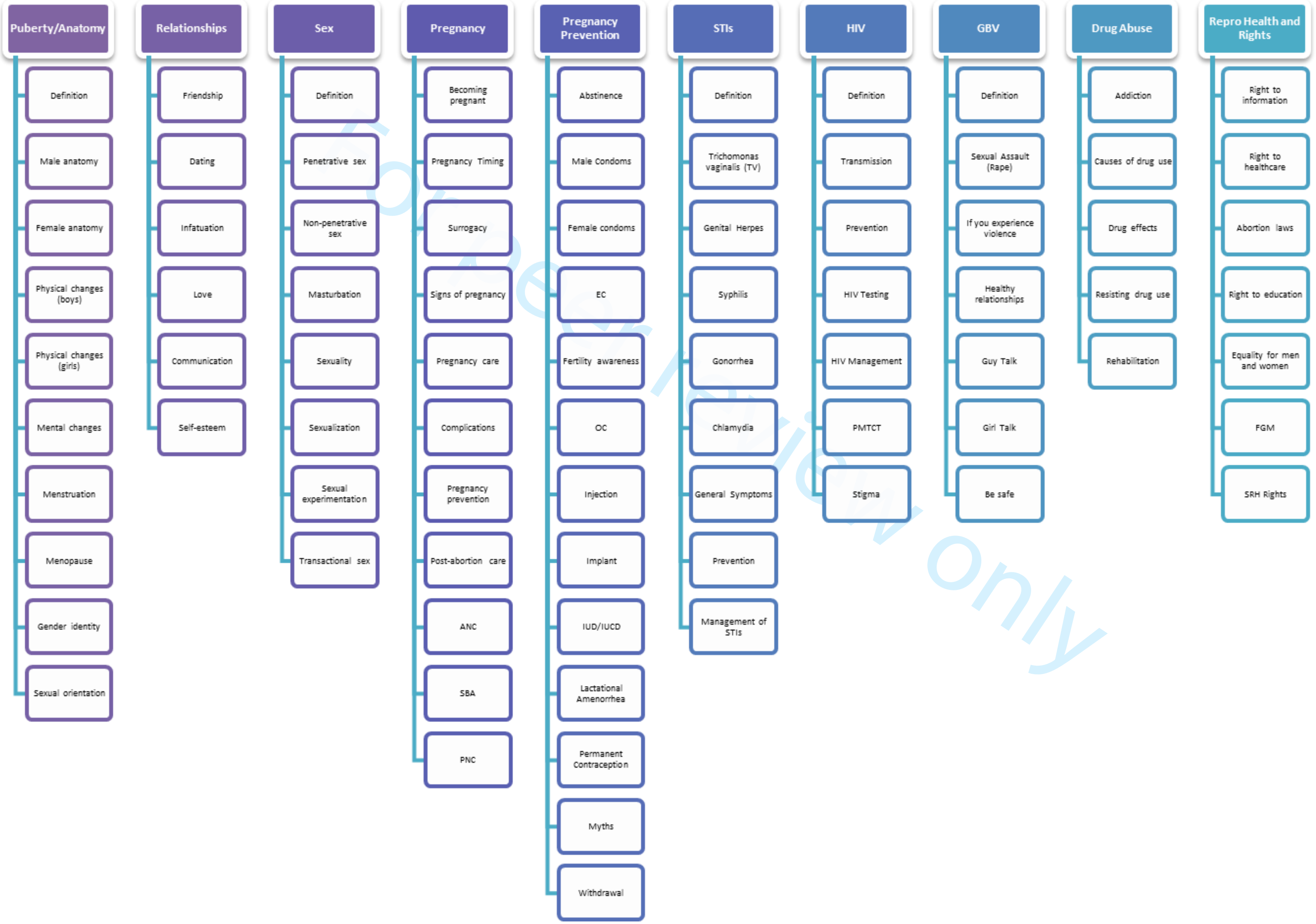

For peer review only - http://bmjopen.bmj.com/site/about/guidelines.xhtml

CONSORT 2010 checklist of information to include when reporting a randomised trial*

Section/Topic	Item No	Checklist item	Reported on page No
**Title and abstract**			
	1a	Identification as a randomised trial in the title	1
	1b	Structured summary of trial design, methods, results, and conclusions (for specific guidance see CONSORT for abstracts)	1-2
**Introduction**			
Background and objectives	2a	Scientific background and explanation of rationale	3-4
	2b	Specific objectives or hypotheses	4
**Methods**			
Trial design	3a	Description of trial design (such as parallel, factorial) including allocation ratio	4
	3b	Important changes to methods after trial commencement (such as eligibility criteria), with reasons	N/A
Participants	4a	Eligibility criteria for participants	5
	4b	Settings and locations where the data were collected	5
Interventions	5	The interventions for each group with sufficient details to allow replication, including how and when they were actually administered	5-6
Outcomes	6a	Completely defined pre-specified primary and secondary outcome measures, including how and when they were assessed	6-7
	6b	Any changes to trial outcomes after the trial commenced, with reasons	N/A
Sample size	7a	How sample size was determined	7
	7b	When applicable, explanation of any interim analyses and stopping guidelines	N/A
Randomisation:			
Sequence generation	8a	Method used to generate the random allocation sequence	7
	8b	Type of randomisation; details of any restriction (such as blocking and block size)	7
Allocation concealment mechanism	9	Mechanism used to implement the random allocation sequence (such as sequentially numbered containers), describing any steps taken to conceal the sequence until interventions were assigned	7
Implementation	10	Who generated the random allocation sequence, who enrolled participants, and who assigned participants to interventions	7
Blinding	11a	If done, who was blinded after assignment to interventions (for example, participants, care providers, those	N/A

For peer review only - http://bmjopen.bmj.com/site/about/guidelines.xhtml

		assessing outcomes) and how	
	11b	If relevant, description of the similarity of interventions	6
Statistical methods	12a	Statistical methods used to compare groups for primary and secondary outcomes	7-9
	12b	Methods for additional analyses, such as subgroup analyses and adjusted analyses	9
**Results**			
Participant flow (a diagram is strongly recommended)	13a	For each group, the numbers of participants who were randomly assigned, received intended treatment, and were analysed for the primary outcome	10-11(Figure 1)
	13b	For each group, losses and exclusions after randomisation, together with reasons	(Figure 1)
Recruitment	14a	Dates defining the periods of recruitment and follow-up	(Figure 1)
	14b	Why the trial ended or was stopped	N/A
Baseline data	15	A table showing baseline demographic and clinical characteristics for each group	(Table 1)
Numbers analysed	16	For each group, number of participants (denominator) included in each analysis and whether the analysis was by original assigned groups	(Table 1)
Outcomes and estimation	17a	For each primary and secondary outcome, results for each group, and the estimated effect size and its precision (such as 95% confidence interval)	13-15 (Table 2 and 3)
	17b	For binary outcomes, presentation of both absolute and relative effect sizes is recommended	N/A
Ancillary analyses	18	Results of any other analyses performed, including subgroup analyses and adjusted analyses, distinguishing pre-specified from exploratory	12-13 (Table 2 and 3)
Harms	19	All important harms or unintended effects in each group (for specific guidance see CONSORT for harms)	N/A
**Discussion**			
Limitations	20	Trial limitations, addressing sources of potential bias, imprecision, and, if relevant, multiplicity of analyses	18-19
Generalisability	21	Generalisability (external validity, applicability) of the trial findings	17-18
Interpretation	22	Interpretation consistent with results, balancing benefits and harms, and considering other relevant evidence	19
**Other information**			
Registration	23	Registration number and name of trial registry	10
Protocol	24	Where the full trial protocol can be accessed, if available	Cited (ref: 26)
Funding	25	Sources of funding and other support (such as supply of drugs), role of funders	19

*We strongly recommend reading this statement in conjunction with the CONSORT 2010 Explanation and Elaboration for important clarifications on all the items. If relevant, we also recommend reading CONSORT extensions for cluster randomised trials, non-inferiority and equivalence trials, non-pharmacological treatments, herbal interventions, and pragmatic trials. Additional extensions are forthcoming: for those and for up to date references relevant to this checklist, see www.consort-statement.org.

For peer review only - http://bmjopen.bmj.com/site/about/guidelines.xhtml
